# Candidate genes for mastitis resistance in dairy cattle: a data integration approach

**DOI:** 10.1186/s40104-022-00821-0

**Published:** 2023-02-10

**Authors:** Zala Brajnik, Jernej Ogorevc

**Affiliations:** Biotechnical Faculty, Department of Animal Science, University of Ljubljana, Groblje 3, Domzale, SI-1230 Slovenia

**Keywords:** Association study, Candidate genes, Epigenetics, Mammary gland, Mastitis, Quantitative trait loci

## Abstract

**Background:**

Inflammation of the mammary tissue (mastitis) is one of the most detrimental health conditions in dairy ruminants and is considered the most economically important infectious disease of the dairy sector. Improving mastitis resistance is becoming an important goal in dairy ruminant breeding programmes. However, mastitis resistance is a complex trait and identification of mastitis-associated alleles in livestock is difficult. Currently, the only applicable approach to identify candidate loci for complex traits in large farm animals is to combine different information that supports the functionality of the identified genomic regions with respect to a complex trait.

**Methods:**

To identify the most promising candidate loci for mastitis resistance we integrated heterogeneous data from multiple sources and compiled the information into a comprehensive database of mastitis-associated candidate loci. Mastitis-associated candidate genes reported in association, expression, and mouse model studies were collected by searching the relevant literature and databases. The collected data were integrated into a single database, screened for overlaps, and used for gene set enrichment analysis.

**Results:**

The database contains candidate genes from association and expression studies and relevant transgenic mouse models. The 2448 collected candidate loci are evenly distributed across bovine chromosomes. Data integration and analysis revealed overlaps between different studies and/or with mastitis-associated QTL, revealing promising candidate genes for mastitis resistance.

**Conclusion:**

Mastitis resistance is a complex trait influenced by numerous alleles. Based on the number of independent studies, we were able to prioritise candidate genes and propose a list of the 22 most promising. To our knowledge this is the most comprehensive database of mastitis associated candidate genes and could be helpful in selecting genes for functional validation studies.

**Supplementary Information:**

The online version contains supplementary material available at 10.1186/s40104-022-00821-0.

## Introduction

Inflammation of the mammary tissue is one of the most common harmful health issues in dairy ruminants and is considered the most economically important infectious disease of the dairy sector. The major consequences of mastitis are reduced milk production and veterinary costs [1], animal welfare concerns [2, 3], extensive use of antibiotics contributing to increasing antimicrobial resistance [4], and impact on the safety and quality of milk and dairy products [5, 6]. The improvement in milk yield has been tremendous in the last decades, doubling milk production in the last fifty years [7]. However, intensive selection focused on milk yield led to deterioration of animal fertility and mammary health traits [8, 9]. Selection for high milk flow, preferred due to machine milking, resulted in weakening of the mammary streak canal sphincter that represents a physical barrier for pathogen entry [10]. Characteristics of milk flow were clearly associated with mammary health status and correlated indicators (e.g., somatic cell score – SCC) [11–13]. Unfavourable correlations between milk production traits and mastitis can be offset to some extent by appropriate herd management [14], but mammary infections and fertility problems are generally more common in high producing herds [15–17]. The situation is somewhat different in the Nordic countries, where direct and indirect mastitis indicators (such as clinical mastitis and somatic cell count) are routinely measured and have been used in breeding programmes to select for mastitis resistance since the 1990s [18]. Despite the low heritability of the traits, such phenotype-based selection has been shown to be beneficial in improving mastitis resistance [19], but causal genetic mechanisms behind the traits remain unknown.

The conventional method of treating mastitis is intramammary administration of antibiotics, which is not always effective [20], and has other adverse effects (e.g., antimicrobial resistance, contamination of dairy products). In addition, the relative efficacy of intramammary therapies using different antimicrobials to treat mastitis is difficult to assess and remains inconclusive [21]. Improving disease resistance is therefore considered a priority goal in modern breeding programmes [22, 23]. There is an increasing trend towards monitoring the physiological status and welfare of farm animals in real-time by recording parameters directly on the animal using electronic devices (so called “indicator traits”) [24], which could also be used for early detection of mammary infections.

The discovery of candidate (causative) genes for complex traits in large livestock is difficult because of the high breeding costs of experimental herds, long generation intervals, inefficient genetic engineering techniques, and the heterogeneous genetic background of outbred ruminant populations that exhibit population-specific genetic interactions between loci. The situation is quite different in model organisms (e.g., mice) where inbred lines and well-established genetic manipulation techniques (targeted gene disruptions/alterations) are available that can directly reveal phenotypes associated loci. Genetically modified mouse models are therefore a valuable source of data on traits of interest in all mammalian species. The mouse model has been used extensively as a tool to identify gene functions. Despite some anatomical and physiological differences between mice and ruminants, mouse models provide a cost-effective alternative for studying intramammary infections [25]. On the other hand, in large farm animals, as suggested by Mackay [26], the only applicable approach for identifying and prioritising quantitative trait loci (QTL) and candidate genes is to integrate diverse information that all together demonstrate the involvement of identified candidate genomic regions in a complex phenotype. However, despite the efforts invested in candidate gene discovery in large farm animals over the past decades, the success of the classical QTL-to-candidate gene approach has been limited. Successful examples of quantitative trait nucleotide (QTN) identification in dairy cattle include single nucleotide polymorphisms (SNPs) associated with milk composition and yield in *DGAT1* [27], *GHR* [28] and *ABCG2* [29]. To our knowledge, no causative gene/QTN for mastitis resistance has yet been validated due to the complexity of the trait, specific host-pathogen interactions, and other factors that complicate identification and validation of candidate genes.

In this study, we performed an extensive literature data mining and collected heterogeneous mastitis-associated data from various sources, including different ruminant species (sheep, goat, and cattle) and mastitis associated mouse models. The relevant information has been integrated into a database of bovine candidate genes for mastitis resistance, which we hope will be useful to researchers investigating the genetic background of mastitis resistance. The database represents a collection of known candidate genes for mastitis resistance, which can be prioritised according to different criteria and validated in functional studies for possible detection of mastitis-associated QTN.

## Materials and methods

### Data collection

Mastitis-associated loci were collected by manual review of the relevant literature and extraction of data from various sources. Mastitis-associated candidate genes reported in association and expression studies were collected by searching for relevant publications in PubMed [30] (using combinations of keywords: “mastitis”, “somatic cells”, “mammary”, “infection”, “QTL”, “SNP”, “association”, “expression”, and “candidate genes”). For association studies, all relevant articles on ruminants were manually reviewed and candidate genes with reported associations to mastitis were included in the database. Regarding expression studies in ruminants, it is impossible to extract all relevant information from the literature because of the large amount of experimental data available. Therefore, we have attempted to provide a representative sample of the available transcriptomic data indicative of expression changes during mastitis with the most common mastitis-causing pathogens (e.g., *Escherichia coli* and *Staphylococcus aureus*). In the present database, we included mastitis associated expression data from a database published in 2009 [31] and updated the collection of candidate genes with more recent expression data obtained from studies combining heterogeneous transcriptomic information (meta-analyses) and studies selected as most relevant, based on our review and meeting specific criteria (published after 2009, reported differentially expressed in controlled infections, performed on ruminant in vitro or in vivo challenge systems, studying immune response against the most common mastitis pathogens – including *E. coli* or *S. aureus* or their virulence factors, and using microarrays or RNA sequencing methods) (e.g., [32–39]). The genes reported differentially expressed in the selected studies were included to the database.

Mouse knockout and transgene model data were retrieved from the Mouse Genome Informatics (MGI) database 6.13 [40] using the phenotype ontology term “mastitis”. The availability of well-annotated mouse and bovine genomes allows identification of orthologs between the species, enabling use of a comparative approach. Therefore, for candidate genes associated with mastitis phenotype in mouse models, cattle orthologs were included in the database.

QTL in cattle were examined at Animal QTL Database [41], specifically in the Cattle QTL database [42] using traits view “Cattle QTLdb trait hierarchies”, selecting “mastitis” in “health traits” category and considering all mastitis-associated traits; that is “somatic cell score” (SCS), “somatic cell count” (SCC), and “clinical mastitis” (CM).

### Data analysis

Candidate genes reported by multiple independent studies, regardless of study approach, were considered the most promising. The most promising candidates were screened for positional overlaps with mastitis associated QTL (CM, SCS, and SCC). Physical positions of mastitis associated QTL were extracted from the Cattle QTLdb using “QTL/associations analysis tool” that enables data download and QTL locations compared with the genomic positions of candidate genes extracted from GenBank [43], using annotations from bovine genome assembly ARS-UCD1.2 [44]. In case candidate gene shared at least part of its sequence with QTL of interest we considered the QTL as overlapping.

Pathway enrichment analysis of all the collected candidate genes was performed using g:Profiler [45]. The standard analysis implemented in g:Profiler searched for pathways, biological processes (BP), molecular functions (MF), and cellular components (CC) in which genes from the collected candidate gene list were significantly enriched compared to all genes in the genome. For the most promising genes pathway redundancy was addressed with EnrichmentMap [46] and visualised in Cytoscape [47]. The list of the most promising candidate genes was analysed for protein-protein interactions using STRING database [48]. Lists of candidate genes obtained by different approaches in ruminants were compared using an interactive tool for comparing lists with Venn’s diagrams [49].

## Results

**Fig. 1:**
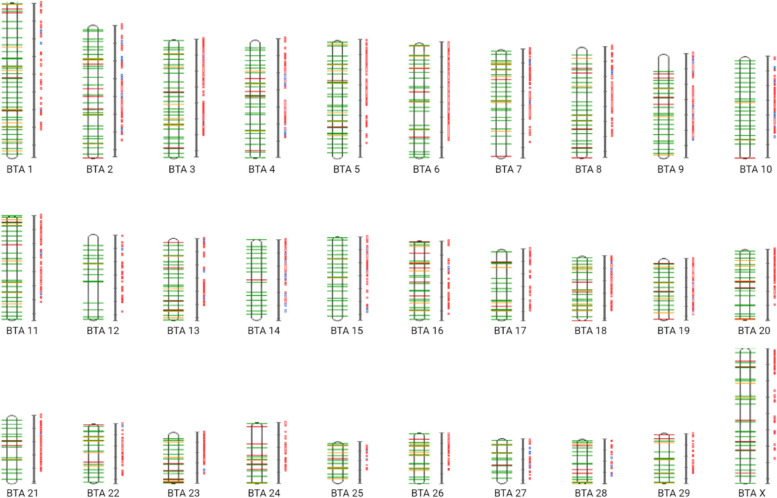
Schematic overview of the chromosomal arrangement of the collected mastitis-associated candidate genes (left – physical map) and QTL (right – linkage map) from Cattle QTLdb. Candidate genes involved in the immune response to *Escherichia coli* infection are highlighted in green, those involved in *Staphylococcus aureus* infection are highlighted in orange, whereas red indicates candidate genes associated with other pathogens. Red colour in linkage maps represents significant QTL and blue represents suggestive

We collected 157 candidate genes from association studies, 2300 candidate genes from expression studies, and six genes from mouse models yielding a total of 2448 candidate genes that are likely to represent a genetic background for mastitis resistance. In addition, there are currently 668 QTL directly associated with mastitis or correlated traits (i.e., clinical mastitis, somatic cell score, and somatic cell count) reported in Cattle QTLdb. The identified candidate genes and QTL show an even distribution across all bovine autosomes and chromosome X (Fig. 1).

### Transgenic and knockout models in mice

In the MGI database, we found one gene disruption (*Mfge8*) and five transgenic mouse models for *Lao1, Enpp2, Lpar1, Lpar2*, and *Lpar3* that resulted in abnormal mammary gland physiology and were also associated with mastitis (Additional file 1: Table S1). Bovine orthologs of the *Mfge8* [50] and *Lpar3* [51] associated with mastitis phenotypes in knockout and transgenic mouse models were also reported differentially expressed during mammary infection in cattle.

### Association studies

Associations between genotypes (mainly SNPs) and mastitis-associated phenotypes (mainly SCS) have been demonstrated for 157 candidate genes in ruminants (Additional file 1: Table S2), several of which have been reported in multiple independent association studies (e.g., *CXCL8*, *CXCR1*, *TLR4*). Polymorphisms in genes associated with mastitis resistance represent potential causative mutations (QTNs).

### Transcriptomic studies

We found 2300 candidate genes differentially expressed during mastitis in transcriptomic studies (Additional file 1: Table S3). When considering the pathogen-specific response [52], there were 1825 genes reported differentially expressed during *Escherichia coli* (*E.coli*) infection, 480 during *Staphylococcus aureus* (*S. aureus*) infection, of which 231 were differentially regulated in response to both pathogens, while the rest are associated with other pathogens. Some of the genes differentially regulated during mastitis have been reported in multiple independent expression studies (e.g., *CXCL8*, *CXCR1*, *LTF*, *TLR4*).

### QTL

The list of QTL associated with mastitis-related traits (CM, SCC, and SCS) currently includes 668 QTL in Cattle QTLdb. The number of mastitis associated QTL per different bovine chromosomes ranged from 4 (BTA17 and 24) to 49 (BTA9). The highest density of mastitis-associated QTL is located on chromosomes 9 (49 QTL), 14 (45), 5 (43), and 10 (42).

### The most promising candidate genes

Candidate genes associated with mastitis in multiple independent studies represent candidate genes of particular interest (Table 1). Candidates found by a high number of independent studies and/or in studies using different research approaches (e.g., *CXCL8*, *CXCR1*) could be considered of the highest priority for validation studies.

**Table 1:** List of the most promising (associated with mastitis in multiple independent studies, regardless of study approach) candidate genes for mastitis resistance

Gene	Gene name	Chr.	Ensembl ID (*Bos Taurus*)	Association studies (*n*)	Expression studies (*n*)	Mouse models	Overlapping QTL^a^	References
*BoLA-DRB3*	Major histocompatibility complex, class II, DRB3	23	ENSBTAG00000013919	13	/		SCS	[31, 53–64]
*C6*	Complement C6	20	ENSBTAG00000014177	3	2		/	[65–69]
*C9*	Complement C9	20	ENSBTAG00000016149	4	1		/	[66–68, 70, 71]
*CACNA2D1*	Calcium voltage-gated channel auxiliary subunit alpha2delta 1	4	ENSBTAG00000020569	4	2		/	[60, 72–76]
*CARD6*	Caspase recruitment domain family member 6	20	ENSBTAG00000014374	3	/		/	[66, 68, 77]
*CD14*	CD14 molecule	7	ENSBTAG00000015032	4	3		/	[31, 34, 37, 78–81]
*CXCL8*	C-X-C motif chemokine ligand 8 (interleukin 8)	6	ENSBTAG00000019716	18	10		CM	[31, 32, 35, 82–105]
*CXCR1*	Chemokine (C-X-C motif) receptor 1	2	ENSBTAG00000026753	21	4		/	[31, 82, 83, 106–126]
*CXCR2*	C-X-C motif chemokine receptor 2	2	ENSBTAG00000038042	3	/		/	[127–129]
*DCK*	Deoxycytidine kinase	6	ENSBTAG00000012397	4	1		/	[70, 118, 130, 131]
*DGAT1*	Diacylglycerol O-acyltransferase 1	14	ENSBTAG00000026356	5	1		/	[70, 130, 132–135]
*GC*	GC vitamin D binding protein	6	ENSBTAG00000013718	7	/		SCS	[71, 131, 132, 134, 136–138]
*LTF*	Lactotransferrin	22	ENSBTAG00000001292	11	7		/	[31, 33, 35, 37, 38, 66, 124, 139–149]
*LPAR3*	Lysophosphatidic acid receptor 3	3	ENSBTAG00000003791	/	1	Tg	/	[32, 51]
*MBL1*	Mannose binding lectin, liver (A)	28	ENSBTAG00000054761	6	/		/	[118, 150–154]
*MBL2*	Mannose binding lectin 2	26	ENSBTAG00000007049	3	/		/	[118, 153, 155]
*MFGE8*	Milk fat globule EGF and factor V/VIII domain containing	21	ENSBTAG00000003300	/	1	KO	/	[37, 50]
*NPFFR2*	Neuropeptide FF receptor 2	6	ENSBTAG00000009070	4	/		SCS	[130, 132, 136, 156]
*SOCS2*	Suppressor of cytokine signaling 2	5	ENSBTAG00000012007	4	4		/	[35, 68, 157–162]
*STAT5A*	Signal transducer and activator of transcription 5 A	19	ENSBTAG00000009496	3	/		CM	[163–165]
*TLR2*	Toll like receptor 2	17	ENSBTAG00000008008	7	10		/	[31, 34, 35, 37, 97, 105, 120, 166–175]
*TLR4*	Toll like receptor 4	8	ENSBTAG00000006240	11	17		/	[31, 34, 38, 76, 104, 105, 140, 147, 149, 166, 168, 170, 173, 175–188]

*Chr.* Chromosome, *QTL* Quantitative trait locus, *SCS* Somatic cell score, *CM* Clinical mastitis, *Tg* Transgenic, *KO* Knockout

^a^QTL genomic location that overlaps with candidate gene genomic location

### Enrichment analysis

**Fig. 2:**
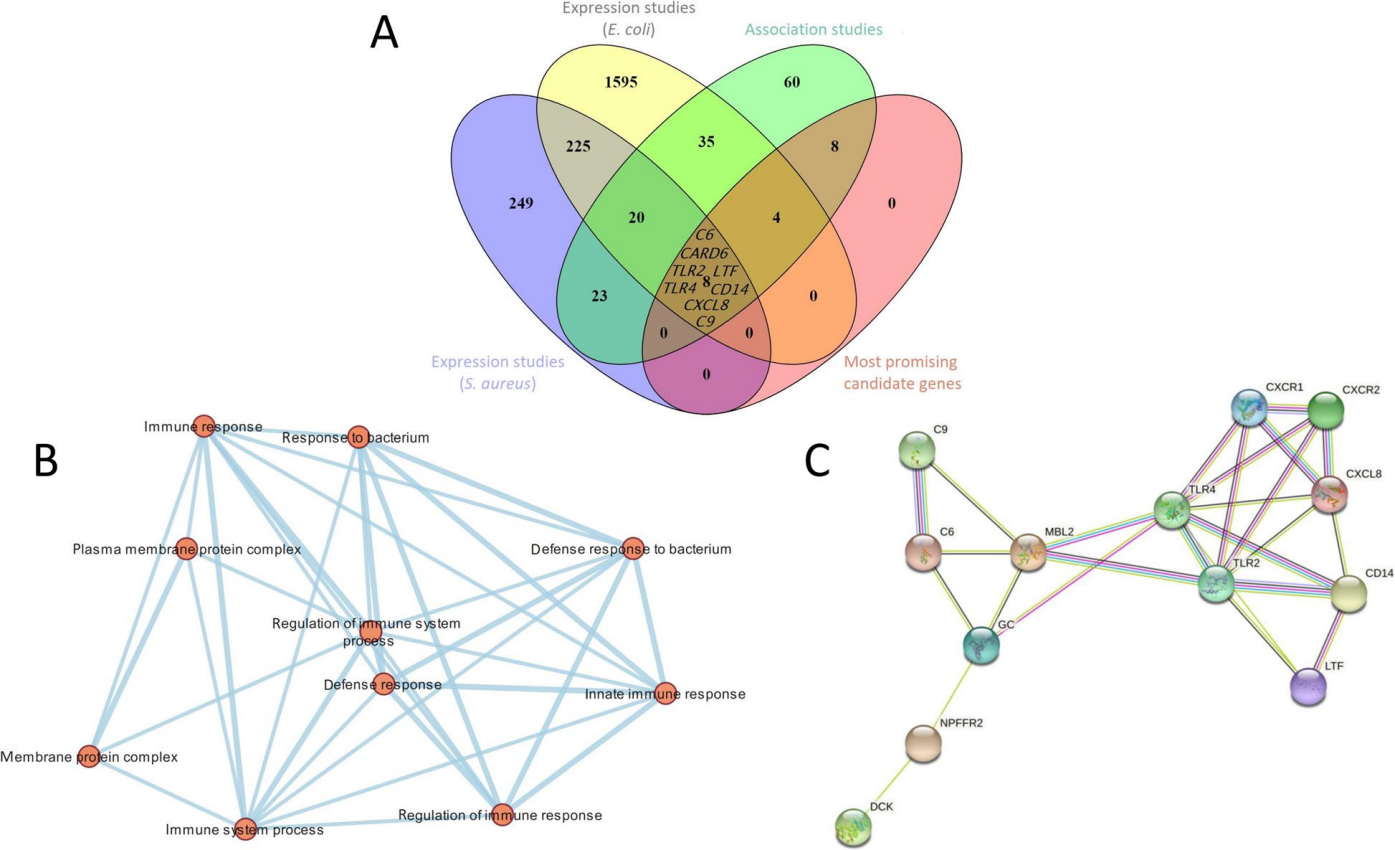
Results of the candidate gene analysis. **A** Overlaps between the collected candidate genes found in different studies on ruminants compared to the list of candidate genes reported in multiple studies (considered the most promising candidate genes), showing eight genes in the cross section with reported sequence polymorphisms associated with mastitis resistance and differentially expressed during *E. coli* and *S. aureus* intramammary infections. **B** Pathway enrichment analysis using g:Profiler and Cytoscape showing major biological pathways within the analysed gene set of the most promising candidates (node cutoff: *P* value = 0.01; *Q* value = 0.01. **C** Protein-protein interactions of the 22 most promising candidate genes as suggested by STRING database of which 13 are connected in a network. Different edge colours represent different types of associations (turquoise: known interactions – from curated databases; magenta: known interactions – experimentally determined; green: predicted interactions – gene neighbourhood; red: predicted interactions – gene fusions; blue: predicted interactions – gene co-occurrence; lime: others – text mining; black: others – co-expression; lavender: others – protein homology)

The g:Profiler search for the collected candidate genes, revealed the most enriched pathways from KEGG and REACTOME databases (Table 2). Functional enrichment analysis mapped our gene list to known sources of functional information and identified statistically significant enriched biological processes (BP), molecular functions (MF) and cellular components (CC), which clearly show the association of the candidate genes to immune response related activities (Table 3; Fig. 2).

**Table 2:** Significantly enriched pathways for the input list of the collected candidate genes obtained from KEGG and REACTOME

**KEGG: Term name**	**Term ID**	***P***_**adj**_	**Genes in pathway**
ABC transporters	KEGG:02010	6.115 × 10^−12^	*ABCA4*, *ABCA7*, *ABCB1*, *ABCC2*, *ABCD4*, *ABCG2*, *ABCG5*
Viral protein interaction with cytokine and cytokine receptor	KEGG:04061	3.263 × 10^−8^	*ACKR3*, *CCL11*, *CCL16*, *CCL19*, *CCL2*, *CCL20*, *CCL28*, *CCL3*, *CCL4*, *CCL5*, *CCR1*, *CCR2*, *CCR5*, *CCR7*, *CXCL1*, *CXCL10*, *CXCL11*, *CXCL2*, *CXCL5*, *CXCL8*, *CXCR1*, *CXCR2*, *CXCR4*, *GRO1*, *IL10RA*, *IL10RB*, *IL18*, *IL18RAP*, *IL2*, *IL20RA*, *IL20RB*, *IL2RG*, *IL34*, *IL6*
Bile secretion	KEGG:04976	3.718 × 10^−4^	*ABCB1*, *ABCC2*, *ABCG2*, *ABCG5*
Cytokine-cytokine receptor interaction	KEGG:04060	6.366 × 10^−4^	*ACKR3*, *AMH*, *BMP7*, *CCL11*, *CCL16*, *CCL19*, *CCL2*, *CCL20*, *CCL28*, *CCL3*, *CCL4*, *CCL5*, *CCR1*, *CCR2*, *CCR5*, *CCR7*, *CD4*, *CD40*, *CSF2*, *CSF2RB*, *CSF3*, *CX3CL1*, *CXCL10*, *CXCL11*, *CXCL16*, *CXCL2*, *CXCL5*, *CXCL8*, *CXCR1*, *CXCR2*, *CXCR4*, *FAS*, *GDF10*, *GDF9*, *GHR*, *GRO1*, *IFNAR2*, *IFNB*, *IFNB2*, *IL10RA*, *IL10RB*, *IL11*, *IL17A*, *IL17F*, *IL18*, *IL18RAP*, *IL1A*, *IL1B*, *IL1RAP*, *IL1RN*, *IL2*, *IL20RA*, *IL20RB*, *IL21*, *IL23A*, *IL2RG*, *IL31RA*, *IL34*, *IL36A*, *IL4R*, *IL6*, *INHBB*
Hematopoietic cell lineage	KEGG:04640	2.008 × 10^−3^	*BOLA-DOB*, *BOLA-DQA2*, *BOLA-DQA5*, *BOLA-DRB3*, *CD14*, *CD1E*, *CD34*, *CD36*, *CD37*, *CD38*, *CD3E*, *CD4*, *CD44*, *CD55*, *CD8A*, *CD8B*
**REACTOME: Term name**	**Term ID**	***P***_**adj**_	**Genes in pathway**
Chemokine receptors bind chemokines	REAC:R-BTA-380,108	3.344 × 10^−5^	*ACKR3*, *CCL11*, *CCL16*, *CCL19*, *CCL2*, *CCL20*, *CCL28*, *CCL3*, *CCL4*, *CCL5*, *CCR1*, *CCR2*, *CCR5*, *CCRL2*, *CXCL13*, *CXCL16*, *CXCR4*
Senescence-Associated Secretory Phenotype (SASP)	REAC:R-BTA-2,559,582	4.729 × 10^−2^	*CDK6*, *CDKN2B*, *CDKN2C*, *CEBPB*

*KEGG* Kyoto encyclopedia of genes and genomes, *Padj* adjusted enrichment *P*-values

**Table 3:** Functional enrichment analysis for the input list of the collected candidate genes

GO:BP			GO:MF			GO:CC		
Term name	Term ID	*P*_adj_	Term name	Term ID	*P*_adj_	Term name	Term ID	*P*_adj_
Response to stress	GO:0006950	2.229 × 10^−48^	Chemokine activity	GO:0008009	2.078 × 10^−13^	Extracellular region	GO:0005576	8.251 × 10^−18^
Defense response	GO:0006952	1.292 × 10^−46^	Signaling receptor binding	GO:0005102	4.433 × 10^−12^	Extracellular space	GO:0005615	5.248 × 10^−16^
Immune system process	GO:0002376	5.366 × 10^− 43^	Protein binding	GO:0005515	7.952 × 10^−12^	Cytoplasm	GO:0005737	3.469 × 10^−15^
Response to external stimulus	GO:0009605	1.122 × 10^−37^	Chemokine receptor binding	GO:0042379	3.461 × 10^−11^	Plasma membrane protein complex	GO:0098797	1.408 × 10^−10^
Immune response	GO:0006955	2.454 × 10^−37^	Cytokine activity	GO:0005125	3.795 × 10^−11^	Cell surface	GO:0009986	6.243 × 10^−10^
Response to organic substance	GO:0010033	3.460 × 10^−33^	ABC-type transporter activity	GO:0140359	8.961 × 10^−11^	External side of plasma membrane	GO:0009897	2.348 × 10^−9^
Inflammatory response	GO:0006954	1.027 × 10^−32^	Immune receptor activity	GO:0140375	1.822 × 10^−10^	Cell periphery	GO:0071944	2.209 × 10^−8^
Response to biotic stimulus	GO:0009607	1.247 × 10^−32^	Cytokine receptor activity	GO:0004896	9.780 × 10^−10^	Endomembrane system	GO:0012505	7.691 × 10^−8^
Response to other organism	GO:0051707	2.191 × 10^−32^	Cytokine receptor binding	GO:0005126	3.058 × 10^−9^	Extracellular matrix	GO:0031012	8.426 × 10^−8^
Regulation of immune system process	GO:0002682	3.557 × 10^−32^	Enzyme binding	GO:0019899	5.853 × 10^−9^	External encapsulating structure	GO:0030312	9.481 × 10^−8^

*GO* Gene ontology, *BP* Biological process, *MF* Molecular function, *CC* Cellular component, *P*_adj_ adjusted enrichment *P*-values

## Discussion

The database created provides a comprehensive overview of the current knowledge on the genetic background of mastitis resistance in dairy cattle and could be extrapolated to other dairy ruminant species. The database is a practical example of genetic dissection of a complex trait that could serve as an example for other quantitative phenotypes in different animal species. A similar database for milk- and mastitis-related traits has already been created [31]. This study focuses exclusively on mastitis-related traits and is updated with the newly acquired knowledge.

Over the past decades, numerous experiments (in vivo and in vitro) have been performed to search for mastitis resistance candidate genes at the DNA/RNA level using high-throughput technologies (e.g., RNA-Seq, microarrays). Whole genome/transcriptome studies have generated a tremendous amount of data. Consequently, many loci have been proposed as candidates for mastitis resistance. Large lists of candidate genes present a challenge for researchers in analysing and interpreting data from expression and association experiments. Integration of data and enrichment analyses are needed to gain better mechanistic insight into the genetic background of mastitis resistance. However, the problem with these studies is that they are difficult to integrate and combine because they use different methodological approaches, experimental designs, incubation periods, and pathogen/virulence factors for immune challenges that elicit pathogen-specific immune responses [105]. As a result, studies often show conflicting results, making it difficult to combine data into a meaningful meta-analysis to extract relevant information. We believe that standardisation of such experiments would represent an important advance in the field. In addition, it should be kept in mind that existing reports may lead new studies to focus on already identified candidate genes. Therefore, whole-genome/transcriptome-level studies should be used to collect relevant data and identify potential candidate genes more broadly, followed by targeted functional studies of the most promising candidates.

Genes influencing complex traits are assumed to be additive in their contribution to phenotype and to contribute only to a minor extent. The number (2448 candidate genes and 668 QTL) and distribution of candidate loci across all bovine chromosomes illustrate the complexity of the trait and support the theory of a large number of candidate loci with a small contribution of a single locus to the phenotype. Given the “infinitesimal model” paradigm [189], identification of causative genes for mastitis resistance would be difficult and probably not reasonable in the context of marker-assisted selection (MAS). However, if alleles with moderate to large influence on mastitis resistance were identified, it would be feasible to implement such alleles in breeding strategies via MAS. Combining MAS and genomic selection would likely be an optimal trait improvement strategy in such a case. In addition, there are suggestions that to better understand the genetic architecture of complex traits, the current additive effects-based quantitative genetic framework needs to be improved and refined to include the effects of a range of multi-allelic markers, epigenetic inheritance, and genetic interactions, or even that an entirely new paradigm in quantitative genetic analysis should be proposed [190]. Epigenetic processes have been shown to influence multiple traits, including disease resistance [191–193] and consideration of non-Mendelian inheritance may be a next step toward better understanding the genetic background of complex traits [194]. The effect of clinical mastitis on methylation patterns has been demonstrated in the case of DNA remethylation around the STAT5-binding enhancer in the *CSN1S1* promoter leading to disruption of αS1-casein synthesis during intramammary infection [195]. In addition, microRNAs (miRNAs) [196] and large non-coding RNAs (lncRNAs) [197] play a role in regulating gene expression and may serve as biomarkers for various pathophysiological conditions [198], including mastitis [199].

Integration of the collected candidate genes from multiple sources into a single database, overlaid with known QTL, allows identification of regions densely populated with candidate loci, revealing potential genomic hotspots (positional candidates) for mastitis resistance. Five of the genes considered the most promising candidates (Table 1) (i.e., *BoLA-DRB3*, *CXCL8*, *GC*, *NPFFR2*, and *STAT5A*) overlap with mastitis-associated QTL (CM or SCS). Eight of the most promising candidates (*C6*, *C9*, *CARD6*, *CD14*, *CXCL8*, *LTF*, *TLR2*, *TLR4*) were associated with mastitis in multiple studies and with different study approaches (expression and association studies) and were differentially expressed during infections with *E. coli* and *S. aureus*, respectively (Fig. 2A). Pathway enrichment clearly shows the most promising candidate genes are involved in immune response activation (Fig. 2B). A protein-protein network as suggested by the STRING database shows *C6*, *C9*, *CD14*, *CXCL8*, *LTF*, *TLR2*, *TLR4* (Fig. 2C) are connected in a dense network. This (over)simplified diagram illuminates signal transduction pattern for activation of mammary innate immunity beginning at recognition receptors (TLR2/TLR4) and co-receptors (CD14), which induce chemokines (CXCL8) through G protein-coupled receptors (CXCR1/2), activate complement system (MBL2, C6, C9) and induce expression of antimicrobial peptides (LTF).

An example of a top-priority candidate from our list is *CXCL8* (*IL8*), a known proinflammatory cytokine with a described function in neutrophil chemotaxis [200]. *CXCL8* has been shown to be upregulated in mammary infections and variability in its sequence has been associated with mastitis related traits in several independent studies (Table 1). In addition, the gene overlaps with QTL for clinical mastitis. *CXCL8* could therefore be considered a positional and functional candidate for mastitis resistance.

In 2020 a comprehensive study using a two-step QTL-GWAS approach has been conducted on French dairy cattle breeds that validated a SNP variant (rs436532576) of the *GC* (vitamin D binding protein) as a probable causative variant for mastitis resistance in Holstein breed [201]. *GC* was also identified as one of the most promising genes in our study. Interestingly, it is located in the vicinity of *CXCL8* (around 88.8 Mb on BTA6) and the region around *GC* and *CXCL8* is densely populated with mastitis associated QTL (Cattle QTLdb). *DCK* and *NPFFR2* suggested among the most promising candidates in our study are also located on BTA6, around 86.3 Mb and 87.3 Mb, respectively. Moreover, Cai et al. [202] prioritized mastitis resistance candidate genes using multiple data sources by combining genome-wide association statistics with expression data, and also suggested *GC* as a putative causative gene for clinical mastitis QTL on BTA6, while *DCK* and *NPFFR2* were suggested to be among top-five markers showing significant signals in the QTL region. This identifies BTA6 region between 86 and 89 Mb especially interesting for locating QTN for mastitis resistance. However, in cases where multiple candidate genes are located in proximity to each other it is difficult to determine the causative mutation as the haplotypes may be linked. Despite the fragmentation of mastitis-associated data, the demonstrated consistency between the results of studies, which combine information from multiple sources, supports the validity of the integrative approach we used.

Novel gene editing techniques (e.g., CRISPR, TALEN, ZFN) have dramatically changed and simplified gene modifications, making it possible to generate candidate mutations, edit genomes, and even regulate gene expression of endogenous genes (e.g., transfection of transcription elements fused with CRISPR/dCas9). Combined with protocols for establishing primary ruminant cell cultures [203, 204] and the availability of immortalized ruminant cell lines (e.g., MAC-T [205], BME-UV [206]), a methodological platform is available for generating and validating the suggested candidate mutations in an in vitro setting, which may eventually be followed by (ethically more controversial) gene editing experiments in vivo.

## Conclusion

The compiled database contains 2448 candidate genes associated with mastitis resistance in cattle, which are evenly distributed across bovine chromosomes, illustrating the complexity of the trait. The database provides a comprehensive source of mastitis associated candidate genes available to researchers interested in genetic background of mastitis resistance. The list of the most promising candidate genes represents a priority list for validation in functional studies, which may eventually lead to discovery of QTN and improved mastitis resistance in the future, employing selection or gene editing approaches.

## Supplementary Information

**Additional file 1: Table S1.** Genetically modified mouse models associated with mastitis (MGI). **Table S2.** Associations between genotypes and mastitis related traits (mostly SCS) in dairy ruminants. **Table S3.** Candidate genes differentially expressed during mastitis.

## Data Availability

All data generated or analysed during this study are included in this published article and its supplementary information files.

## Abbreviations

BP: Biological process
CC: Cellular component
CM: Clinical mastitis
CRISPR: Clustered regularly interspaced short palindromic repeats
DNA: Deoxyribonucleic acid
GO: Gene Ontology
KEGG: Kyoto Encyclopaedia of Genes and Genomes
MAS: Marker-assisted selection
MF: Molecular function
MGI: Mouse Genome Informatics
QTL: Quantitative trait loci
QTN: Quantitative trait nucleotide
RNA: Ribonucleic acid
SCC: Somatic cell count
SCS: Somatic cell score
SNPs: Single nucleotide polymorphisms
TALEN: Transcription activator-like effector nucleases
ZFN: Zinc-finger nuclease.
