# Supplementary material for: Candidate genes for mastitis resistance in dairy cattle: a data integration approach
Source: J Anim Sci Biotechnol. 2023 Feb 10;14:10. doi: 10.1186/s40104-022-00821-0 (PMC9912691; doi:10.1186/s40104-022-00821-0)
Supplement: Supplementary file 1 — Additional file 1: Table S1. Genetically modified mouse models associated with mastitis (MGI). Table S2. Associations between genotypes and mastitis related traits (mostly SCS) in dairy ruminants. Table S3. Candidate genes differentially expressed during mastitis. [file 40104_2022_821_MOESM1_ESM.docx]

**Supplementary information**

**Additional file 1:** Table S1: Genetically modified mouse models associated with mastitis (MGI). Table S2: Associations between genotypes and mastitis related traits (mostly SCS) in dairy ruminants. Table S3: Candidate genes differentially expressed during mastitis.

**Table S1** Genetically modified mouse models associated with mastitis (MGI)

| **Associated gene name** | **ENSEMBL ID**  **(*Bos taurus*)** | **MGI ID** | **Description** | **References** |
| --- | --- | --- | --- | --- |
| *Mfge8* | ENSBTAG00000003300 | 3578644 | Milk fat globule EGF and factor V/VIII domain containing | [1] |
| *Enpp2* | ENSBTAG00000013165 | 1321390 | Ectonucleotide pyrophosphatase/phosphodiesterase 2 | [2] |
| *Lao1* | ENSBTAG00000012414 | 6152753 | L-amino acid oxidase 1 | [3] |
| *Lpar1* | ENSBTAG00000009654 | 108429 | Lysophosphatidic acid receptor 1 | [2] |
| *Lpar2* | ENSBTAG00000004652 | 4835234 | Lysophosphatidic acid receptor 2 | [2] |
| *Lpar3* | ENSBTAG00000003791 | 4835238 | Lysophosphatidic acid receptor 3 | [2] |

**Table S2** Associations between genotypes and mastitis related traits (mostly SCS) in dairy ruminants

| **Associated gene name** | **ENSEMBL ID**  **(*Bos taurus*)** | **Description** | **References** |
| --- | --- | --- | --- |
| [*A2M*](http://www.ensembl.org/Bos_taurus/Gene/Summary?db=core;g=ENSBTAG00000018137) | ENSBTAG00000018137 | Alpha-2-macroglobulin | [4, 5] |
| *ACOT2* | ENSBTAG00000046814 | Acyl-CoA thioesterase 2 | [6] |
| *ACOT4* | ENSBTAG00000002843 | Acyl-CoA thioesterase 4 | [6] |
| *ADAMTS3* | ENSBTAG00000006507 | ADAM metallopeptidase with thrombospondin type 1 motif 3 | [7] |
| *ADGRB1* | ENSBTAG00000006385 | Adhesion G protein-coupled receptor B1 | [8, 9] |
| *ADORA2A* | ENSBTAG00000016944 | Adenosine A2a receptor | [10] |
| *ALCAM* | ENSBTAG00000000088 | Activated leukocyte cell adhesion molecule | [11] |
| *APP* | ENSBTAG00000017753 | Amyloid beta precursor protein | [10] |
| *ARHGAP39* | ENSBTAG00000007186 | Rho GTPase activating protein 39 | [12] |
| *ATF7* | ENSBTAG00000005863 | Activating transcription factor 7 | [13] |
| *ATG16L2* | ENSBTAG00000019059 | Autophagy related 16 like 2 | [14] |
| [*ATP1A1*](http://www.ncbi.nlm.nih.gov/sites/entrez?cmd=search&db=gene&term=282144%5buid%5d) | ENSBTAG00000001246 | Sodium/potassium-transporting ATPase subunit alpha-1 | [15] |
| *ATXN7L3B* | ENSBTAG00000035083 | Ataxin 7 like 3B | [14] |
| [*BoLA-DQA2*](https://www.ncbi.nlm.nih.gov/gene/282535) | ENSBTAG00000009656 | Major histocompatibility complex, class II, DQ alpha 2 | [16] |
| *BoLA-DRB3* | ENSBTAG00000013919 | Major histocompatibility complex, class II, DRB3 | [17–30] |
| [*BRCA1*](http://www.ncbi.nlm.nih.gov/sites/entrez?cmd=search&db=gene&term=497014%5buid%5d) | ENSBTAG00000022520 | Breast cancer type 1 susceptibility protein homolog | [31, 32] |
| *C1QTNF3* | ENSBTAG00000017071 | C1q and TNF related 3 | [9, 11] |
| *C3* | ENSBTAG00000017280 | Complement C3 | [33] |
| *C6* | ENSBTAG00000014177 | Complement C6 | [6, 9, 11, 34, 35] |
| *C7* | ENSBTAG00000011766 | Complement C7 | [9, 34] |
| *C9* | ENSBTAG00000016149 | Complement C9 | [9, 11, 34, 36, 37] |
| [*CACNA2D1*](https://www.ncbi.nlm.nih.gov/gene/282608) | ENSBTAG00000020569 | Calcium voltage-gated channel auxiliary subunit alpha2delta 1 | [24, 38–42] |
| *CARD6* | ENSBTAG00000014374 | Caspase recruitment domain family member 6 | [9, 34, 43] |
| *CCL5* | ENSBTAG00000053649 | C-C motif chemokine ligand 5 | [44] |
| *CCNB1* | ENSBTAG00000014239 | Cyclin B1 | [45] |
| *CCR2* | ENSBTAG00000013586 | C-C motif chemokine receptor 2 | [46] |
| *CD14* | ENSBTAG00000015032 | CD14 molecule | [47–52] |
| [*CD4*](http://www.ncbi.nlm.nih.gov/sites/entrez?cmd=search&db=gene&term=281534%5buid%5d) | ENSBTAG00000003255 | T-cell surface glycoprotein CD4 precursor | [53] |
| [*CD46*](https://www.ncbi.nlm.nih.gov/gene/280851) | ENSBTAG00000005397 | CD46 molecule | [54] |
| *CHD1* | ENSBTAG00000020860 | Chromodomain-helicase-DNA-binding protein 1 | [34] |
| *CLU* | ENSBTAG00000005574 | Clusterin Clusterin beta chain Clusterin alpha chain | [55] |
| *CSN1S2* | ENSBTAG00000005005 | Casein alpha-S2 | [56] |
| *CSNK1G3* | ENSBTAG00000017550 | Casein kinase 1 gamma 3 | [14] |
| *CTLA4* | ENSBTAG00000013170 | Cytotoxic T-lymphocyte associated protein 4 | [34] |
| *CXCL8* | ENSBTAG00000019716 | C-X-C motif chemokine ligand 8 (interleukin 8) | [46, 57–81] |
| [*CXCR1*](https://www.ncbi.nlm.nih.gov/gene/100125580) | ENSBTAG00000026753 | Chemokine (C-X-C motif) receptor 1 | [28, 46, 57, 82–102] |
| *CXCR2* | ENSBTAG00000038042 | C-X-C motif chemokine receptor 2 | [103–105] |
| *CYP11B1* | ENSBTAG00000054126 | Cytochrome P450, subfamily XI B, polypeptide 1 | [36] |
| *CYP2R1* | ENSBTAG00000010419 | Cytochrome P450, family 2, subfamily R, polypeptide 1 | [106] |
| *DAB2* | ENSBTAG00000016152 | DAB adaptor protein 2 | [9, 34] |
| *DAPP1* | ENSBTAG00000007921 | Dual adaptor of phosphotyrosine and 3-phosphoinositides 1 | [36] |
| *DCK* | ENSBTAG00000012397 | Deoxycytidine kinase | [36, 94, 107, 108] |
| *DEFB119* | ENSBTAG00000003364 | Defensin beta 119 | [11] |
| *DEFB122* | ENSBTAG00000027383 | Beta-defensin 122 | [11] |
| *DEFB122A* | ENSBTAG00000027384 | Beta-defensin 122a | [11] |
| *DEFB129* | ENSBTAG00000048288 | Defensin beta 129 | [11] |
| *DEFB4A* | ENSBTAG00000053557 | Defensin beta 4A | [109] |
| *DGAT1* | ENSBTAG00000026356 | Diacylglycerol O-acyltransferase 1 | [7, 36, 107, 110–113] |
| *DPP4* | ENSBTAG00000048246 | Dipeptidyl peptidase 4 | [9] |
| *EDN3* | ENSBTAG00000012109 | Endothelin 3 | [36, 107] |
| *EPS15L1* | ENSBTAG00000009990 | Epidermal growth factor receptor pathway substrate 15 like 1 | [14] |
| *FADS2* | ENSBTAG00000015505 | Fatty acid desaturase 2 | [114] |
| *FBXL7* | ENSBTAG00000003219 | F-box and leucine rich repeat protein 7 | [7] |
| [*FEZF2*](https://www.ncbi.nlm.nih.gov/gene/613683) | ENSBTAG00000020302 | FEZ family zinc finger 2 | [28] |
| [*FGF2*](https://www.ncbi.nlm.nih.gov/gene/281161) | ENSBTAG00000005691 | Fibroblast growth factor 2 | [115] |
| *FOS* | ENSBTAG00000004322 | Fos proto-oncogene, AP-1 transcription factor subunit | [45] |
| *FOXK2* | ENSBTAG00000003687 | Forkhead box K2 | [108] |
| *FOXL2* | ENSBTAG00000031277 | Forkhead box L2 | [10] |
| *FREM2* | ENSBTAG00000017032 | FRAS1 related extracellular matrix 2 | [14] |
| *FYB1* | ENSBTAG00000001492 | FYN binding protein 1 | [34, 116] |
| *GAS1* | ENSBTAG00000046803 | Growth arrest specific 1 | [106] |
| *GC* | ENSBTAG00000013718 | GC vitamin D binding protein | [7, 37, 108, 112, 117–119] |
| *GHR* | ENSBTAG00000001335 | Growth hormone receptor | [9] |
| *GPAT3* | ENSBTAG00000017592 | Glycerol-3-phosphate acyltransferase 3 | [106] |
| *GRIK3* | ENSBTAG00000009269 | Glutamate ionotropic receptor kainate type subunit 3 | [14] |
| [*HMGB1*](http://www.ensembl.org/Bos_taurus/Gene/Summary?db=core;g=ENSBTAG00000018103) | ENSBTAG00000018103 | High mobility group protein B1 | [120] |
| *HSPA1A* | ENSBTAG00000025441 | Heat shock protein family A (Hsp70) member 1A | [121, 122] |
| *ICOS* | ENSBTAG00000021596 | Inducible T cell costimulator | [34] |
| *IDH1* | ENSBTAG00000020527 | Isocitrate dehydrogenase (NADP(+)) 1 | [34] |
| *IFI30* | ENSBTAG00000018349 | IFI30 lysosomal thiol reductase | [14] |
| *IFIH1* | ENSBTAG00000008142 | Interferon induced with helicase C domain 1 | [9] |
| *IFNGR1* | ENSBTAG00000008142 | Interferon gamma receptor 1 | [123] |
| *IL10RA* | ENSBTAG00000005215 | Interleukin-10 receptor subunit alpha precursor | [124] |
| *IL1RN* | ENSBTAG00000019665 | Interleukin 1 receptor antagonist | [125] |
| *IL17A* | ENSBTAG00000002150 | Interleukin 17A | [126] |
| *IL17F* | ENSBTAG00000016835 | Interleukin 17F | [126] |
| *IL31RA* | ENSBTAG00000004351 | Interleukin-31 receptor subunit alpha precursor | [11] |
| *IPO11* | ENSBTAG00000018616 | Importin 11 | [14] |
| *ITGB2* | ENSBTAG00000017060 | Integrin subunit beta 2 | [17] |
| *ITGB6* | ENSBTAG00000009080 | Integrin subunit beta 6 | [9] |
| *ITPRID2* | ENSBTAG00000000937 | ITPR interacting domain containing 2 | [10] |
| *JAK2* | ENSBTAG00000012047 | Janus kinase 2 | [127, 128] |
| *JCHAIN* | ENSBTAG00000018531 | Joining chain of multimeric IgA and IgM | [36, 108] |
| *KLHL29* | ENSBTAG00000021969 | Kelch like family member 29 | [129] |
| *LIFR* | ENSBTAG00000010423 | LIF receptor subunit alpha | [43, 107] |
| *LRIT3* | ENSBTAG00000007019 | Leucine rich repeat, Ig-like and transmembrane domains 3 | [14] |
| [*LTF*](https://www.ncbi.nlm.nih.gov/gene/280846) | ENSBTAG00000001292 | Lactotransferrin | [9, 28, 52, 100, 130–143] |
| *LY6D* | ENSBTAG00000034498 | Lymphocyte antigen 6 family member D | [9] |
| *LY6K* | ENSBTAG00000050161 | Lymphocyte antigen 6 family member K | [7, 9] |
| *LY75* | ENSBTAG00000017797 | Lymphocyte antigen 75 | [9] |
| *LYNX1* | ENSBTAG00000005762 | Ly6/neurotoxin 1 | [9] |
| *LYPD2* | ENSBTAG00000016210 | LY6/PLAUR domain containing 2 | [9] |
| *MAP4K4* | ENSBTAG00000013023 | Mitogen-activated protein kinase kinase kinase kinase 4 | [144] |
| *MAPK1* | ENSBTAG00000010312 | Mitogen-activated protein kinase 1 | [11] |
| *MARCHF3* | ENSBTAG00000006797 | Membrane associated ring-CH-type finger 3 | [14] |
| *MAST3* | ENSBTAG00000002347 | Microtubule associated serine/threonine kinase 3 | [14] |
| *MBL1* | ENSBTAG00000054761 | Mannose binding lectin, liver (A) | [94, 145–149] |
| *MBL2* | ENSBTAG00000007049 | Mannose binding lectin 2 | [94, 148, 150] |
| *MTHFR* | ENSBTAG00000020698 | Methylenetetrahydrofolate reductase | [151] |
| *MX2* | ENSBTAG00000008471 | MX dynamin like GTPase 2 | [152] |
| [*NCF1*](https://www.ncbi.nlm.nih.gov/gene/281345) | ENSBTAG00000003305 | Neutrophil cytosolic factor 1 | [153] |
| [*NCF4*](https://www.ncbi.nlm.nih.gov/gene/507859) | ENSBTAG00000007531 | Neutrophil cytosolic factor 4 | [154, 155] |
| *NDUFS6* | ENSBTAG00000009914 | NADH:ubiquinone oxidoreductase subunit S6 | [10] |
| *NFATC2* | ENSBTAG00000018270 | Nuclear factor of activated T-cells 2 | [108, 156] |
| [*NOD2*](https://www.ncbi.nlm.nih.gov/gene/444867) | ENSBTAG00000020936 | Nucleotide binding oligomerization domain containing 2 | [94] |
| *NPFFR2* | ENSBTAG00000009070 | Neuropeptide FF receptor 2 | [7, 107, 117, 118] |
| *NR4A2* | ENSBTAG00000003650 | Nuclear receptor subfamily 4 group A member 2 | [9] |
| *NUDT2* | ENSBTAG00000031793 | Nudix hydrolase 2 | [129] |
| *OSMR* | ENSBTAG00000033107 | Oncostatin M receptor | [9, 34] |
| *OTUD3* | ENSBTAG00000017108 | OTU deubiquitinase 3 | [10] |
| *OXCT1* | ENSBTAG00000033186 | 3-oxoacid CoA-transferase 1 | [9] |
| *P2RY6* | ENSBTAG00000038737 | Pyrimidinergic receptor P2Y6 | [14] |
| *PACRG* | ENSBTAG00000011106 | Parkin coregulated | [13] |
| *PCDH7* | ENSBTAG00000000061 | Protocadherin 7 | [14] |
| *PDGFD* | ENSBTAG00000034827 | Platelet derived growth factor D | [14] |
| [*PGLYRP1*](https://www.ncbi.nlm.nih.gov/gene/282305) | ENSBTAG00000002635 | Peptidoglycan recognition protein 1 | [157] |
| [*PGLYRP2*](https://www.ncbi.nlm.nih.gov/gene/?term=pglyrp2+cattle) | ENSBTAG00000012280 | Peptidoglycan recognition protein 2 | [158] |
| *PHACTR3* | ENSBTAG00000015080 | Phosphatase and actin regulator 3 | [36] |
| *PLOD2* | ENSBTAG00000007331 | Procollagen-lysine,2-oxoglutarate 5-dioxygenase 2 | [14] |
| *PLXNC1* | ENSBTAG00000011245 | Plexin C1 | [34] |
| *PREX1* | ENSBTAG00000015609 | Phosphatidylinositol-3,4,5-trisphosphate dependent Rac exchange factor 1 | [14] |
| *PRLR* | ENSBTAG00000025035 | Prolactin receptor | [9, 129] |
| *PSCA* | ENSBTAG00000047022 | Prostate stem cell antigen | [9] |
| *PTGER4* | ENSBTAG00000014972 | Prostaglandin E receptor 4 | [34] |
| *PTK2B* | ENSBTAG00000005958 | Protein tyrosine kinase 2 beta | [159] |
| *PTX3* | ENSBTAG00000009012 | Pentraxin 3 | [14] |
| *RAD17* | ENSBTAG00000002073 | RAD17 checkpoint clamp loader component | [45] |
| *RARA* | ENSBTAG00000012500 | Retinoic acid receptor alpha | [160] |
| *RASGRP1* | ENSBTAG00000021872 | RAS guanyl releasing protein 1 | [161] |
| *RICTOR* | ENSBTAG00000006697 | RPTOR independent companion of MTOR complex 2 | [36] |
| *SCARA3* | ENSBTAG00000007657 | Scavenger receptor class A member 3 | [14] |
| *SGK1* | ENSBTAG00000004269 | Serum/glucocorticoid regulated kinase 1 | [13] |
| [*SLC11A1*](https://www.ncbi.nlm.nih.gov/gene/282470) | ENSBTAG00000015520 | Solute carrier family 11 member 1 | [90] |
| *SLC4A4* | ENSBTAG00000002348 | Solute carrier family 4 member 4 | [107, 118] |
| *SLC9A8* | ENSBTAG00000008969 | Solute carrier family 9 member A8 | [108] |
| *SLURP1* | ENSBTAG00000016209 | Secreted LY6/PLAUR domain containing 1 | [9] |
| *SLX4IP* | ENSBTAG00000051621 | SLX4 interacting protein | [14] |
| *SOCS2* | ENSBTAG00000012007 | Suppressor of cytokine signaling 2 | [34, 139, 162–167] |
| *SPI1* | ENSBTAG00000021709 | Spi-1 proto-oncogene | [168] |
| *SPP1* | ENSBTAG00000005260 | Osteopontin precursor | [58, 169] |
| *STAB2* | ENSBTAG00000006010 | Stabilin 2 | [14] |
| *STAT3* | ENSBTAG00000021523 | Signal transducer and activator of transcription 3 | [160] |
| *STAT5A* | ENSBTAG00000009496 | Signal transducer and activator of transcription 5A | [111, 127, 160, 170–175] |
| *STAT5B* | ENSBTAG00000010125 | Signal transducer and activator of transcription 5B | [53, 128, 160] |
| *SYK* | ENSBTAG00000004767 | Spleen associated tyrosine kinase | [159] |
| *THEM6* | ENSBTAG00000005063 | Thioesterase superfamily member 6 | [9] |
| *TLR2* | ENSBTAG00000008008 | Toll like receptor 2 | [51, 52, 71, 73, 81, 96, 139, 176-187] |
| [*TLR4*](https://www.ncbi.nlm.nih.gov/gene/281536) | ENSBTAG00000006240 | Toll like receptor 4 | [5, 42, 51, 71, 72, 80, 81, 131, 140, 141, 143, 176, 177, 179, 181, 182, 185, 187-200] |
| *TLR6* | ENSBTAG00000014031 | Toll like receptor 6 | [179] |
| *TNF* | ENSBTAG00000025471 | Tumor necrosis factor | [132, 201, 202] |
| *TNFRSF21* | ENSBTAG00000020054 | TNF receptor superfamily member 21 | [159] |
| *TRAPPC9* | ENSBTAG00000013955 | Trafficking protein particle complex 9 | [12] |
| *TSNARE1* | ENSBTAG00000009974 | T-SNARE domain containing 1 | [9] |
| *TXNRD2* | ENSBTAG00000043581 | Thioredoxin reductase 2 | [10] |
| *VEPH1* | ENSBTAG00000024643 | Ventricular zone expressed PH domain containing 1 | [14] |
| *VNN1* | ENSBTAG00000015094 | Vanin 1 | [123] |
| *VNN2* | ENSBTAG00000016357 | Vanin 2 | [123] |
| *WDR11* | ENSBTAG00000021540 | WD repeat domain 11 | [14] |

**Table S3**: Candidate genes differentially expressed during mastitis.

| **Associated Gene Name** | **ENSEMBL ID (*Bos taurus*)** | **Description** | **References** |
| --- | --- | --- | --- |
| *ABAT* | ENSBTAG00000004038 | 4-aminobutyrate aminotransferase | [203] |
| *ABCA7* | ENSBTAG00000020766 | ATP binding cassette subfamily A member 7 | [203] |
| *ABCB1* | ENSBTAG00000005997 | Multidrug resistance protein 1 | [203] |
| *ABCC2* | ENSBTAG00000008936 | ATP binding cassette subfamily C member 2 | [203] |
| *ABCD4* | ENSBTAG00000014633 | ATP binding cassette subfamily D member 4 | [203] |
| *ABCE1* | ENSBTAG00000019854 | ATP binding cassette subfamily E member 1 | [140] |
| *ABCG2* | ENSBTAG00000017704 | ATP binding cassette subfamily G member 2 | [138] |
| *ABCG5* | ENSBTAG00000016365 | ATP binding cassette subfamily G member 5 | [203] |
| *ABHD1* | ENSBTAG00000007077 | Abhydrolase domain containing 1 | [139] |
| *ABHD18* | ENSBTAG00000010630 | Abhydrolase domain containing 18 | [203] |
| *ABHD2* | ENSBTAG00000019954 | Abhydrolase domain containing 2 | [139] |
| *ABHD5* | ENSBTAG00000008416 | Abhydrolase domain containing 5 | [139] |
| *ABI2* | ENSBTAG00000011017 | Abl interactor 2 | [203] |
| *ABI3* | ENSBTAG00000000032 | ABI family member 3 | [203] |
| *ABI3BP* | ENSBTAG00000026836 | ABI family member 3 binding protein | [139] |
| *ABLIM1* | ENSBTAG00000004899 | Actin binding LIM protein 1 | [140] |
| *ABO* | ENSBTAG00000012525 | Alpha 1-3-N-acetylgalactosaminyltransferase | [203] |
| *ABRA* | ENSBTAG00000018352 | Actin binding Rho activating protein | [203] |
| *ABRAXAS2* | ENSBTAG00000023840 | Abraxas 2, BRISC complex subunit | [203] |
| *ACE3* | ENSBTAG00000038138 | Angiotensin I converting enzyme (peptidyl-dipeptidase A) 3 | [203] |
| *ACKR3* | ENSBTAG00000018424 | Atypical chemokine receptor 3 | [203] |
| *ACOT11* | ENSBTAG00000039686 | Acyl-CoA thioesterase 11 | [140] |
| *ACP2* | ENSBTAG00000021002 | Acid phosphatase 2, lysosomal | [51] |
| *ACP5* | ENSBTAG00000004826 | Acid phosphatase 5, tartrate resistant | [203] |
| *ACSF2* | ENSBTAG00000021301 | Acyl-CoA synthetase family member 2 | [204] |
| *ACSL6* | ENSBTAG00000019708 | Acyl-CoA synthetase long chain family member 6 | [51, 138] |
| *ACSM3* | ENSBTAG00000006447 | Acyl-CoA synthetase medium chain family member 3 | [140] |
| *ACTA1* | ENSBTAG00000046332 | Actin alpha 1, skeletal muscle | [203] |
| *ACTN2* | ENSBTAG00000009696 | Actinin alpha 2 | [203] |
| *ACTR3* | ENSBTAG00000003401 | Actin related protein 3 | [140] |
| *ACYP1* | ENSBTAG00000013992 | Acylphosphatase 1 | [203] |
| *ADAM12* | ENSBTAG00000012444 | ADAM metallopeptidase domain 12 | [203] |
| *ADAM18* | ENSBTAG00000004830 | ADAM metallopeptidase domain 18 | [203] |
| *ADAM19* | ENSBTAG00000003836 | ADAM metallopeptidase domain 19 | [51] |
| *ADAMTS16* | ENSBTAG00000018460 | ADAM metallopeptidase with thrombospondin type 1 motif 16 | [203] |
| *ADAMTS2* | ENSBTAG00000014665 | ADAM metallopeptidase with thrombospondin type 1 motif 2 | [140] |
| *ADAMTS4* | ENSBTAG00000013210 | ADAM metallopeptidase with thrombospondin type 1 motif 4 | [139] |
| *ADAMTS5* | ENSBTAG00000000648 | ADAM metallopeptidase with thrombospondin type 1 motif 5 | [139] |
| *ADAMTSL4* | ENSBTAG00000003604 | ADAMTS like 4 | [138] |
| *ADCY2* | ENSBTAG00000019210 | Adenylate cyclase 2 | [203] |
| *ADCY6* | ENSBTAG00000005464 | Adenylate cyclase 6 | [139] |
| *ADCYAP1R1* | ENSBTAG00000020247 | ADCYAP receptor type I | [203] |
| *ADGRE5* | ENSBTAG00000021818 | Adhesion G protein-coupled receptor E5 | [203] |
| *ADGRL1* | ENSBTAG00000003675 | Adhesion G protein-coupled receptor L1 | [203] |
| *ADGRL2* | ENSBTAG00000012305 | Adhesion G protein-coupled receptor L2 | [203] |
| *ADH6* | ENSBTAG00000006986 | Alcohol dehydrogenase 6 (class V) | [203] |
| *ADHFE1* | ENSBTAG00000004476 | Alcohol dehydrogenase iron containing 1 | [51] |
| *ADI1* | ENSBTAG00000005403 | Acireductone dioxygenase 1 | [138] |
| *ADIG* | ENSBTAG00000031188 | Adipogenin | [203] |
| *ADM* | ENSBTAG00000021048 | Adrenomedullin | [139] |
| *ADPGK* | ENSBTAG00000001578 | ADP dependent glucokinase | [203] |
| *ADPRH* | ENSBTAG00000009391 | ADP-ribosylarginine hydrolase | [203] |
| *ADRB2* | ENSBTAG00000002144 | Adrenoceptor beta 2 | [139] |
| *ADRBK1* | ENSBTAG00000005832 | G protein-coupled receptor kinase 2 | [203] |
| *ADTRP* | ENSBTAG00000019533 | Androgen dependent TFPI regulating protein | [203] |
| *AFM* | ENSBTAG00000047833 | Afamin | [203] |
| *AFP* | ENSBTAG00000017131 | Alpha fetoprotein | [203] |
| *AGAP2* | ENSBTAG00000007156 | ArfGAP with GTPase domain, ankyrin repeat and PH domain 2 | [203] |
| *AGPAT1* | ENSBTAG00000004442 | 1-acylglycerol-3-phosphate O-acyltransferase 1 | [138] |
| *AGR2* | ENSBTAG00000024406 | Anterior gradient 2, protein disulphide isomerase family member | [139] |
| *AGT* | ENSBTAG00000012393 | Angiotensinogen | [204] |
| *AHSA1* | ENSBTAG00000020477 | Activator of HSP90 ATPase activity 1 | [203] |
| *AHSG* | ENSBTAG00000000522 | Alpha 2-HS glycoprotein | [203] |
| *AIF1* | ENSBTAG00000020554 | Allograft inflammatory factor 1 | [139] |
| *AIM1* | ENSBTAG00000017527 | Crystallin beta-gamma domain containing 1 | [204] |
| *AIMP2* | ENSBTAG00000007367 | Aminoacyl tRNA synthetase complex interacting multifunctional protein 2 | [203] |
| *AK3* | ENSBTAG00000017147 | Adenylate kinase 3 | [139] |
| *AK6* | ENSBTAG00000046192 | Adenylate kinase 6 | [203] |
| *AKAP5* | ENSBTAG00000009642 | A-kinase anchoring protein 5 | [203] |
| *AKAP6* | ENSBTAG00000017719 | A-kinase anchoring protein 6 | [203] |
| *AKAP8L* | ENSBTAG00000010439 | A-kinase anchoring protein 8 like | [203] |
| *AKR1C4* | ENSBTAG00000022564 | Aldo-keto reductase family 1, member C4 | [203] |
| *ALB* | ENSBTAG00000017121 | Albumin | [138] |
| *ALDH3A2* | ENSBTAG00000039161 | Aldehyde dehydrogenase 3 family member A2 | [138] |
| *ALDH6A1* | ENSBTAG00000018469 | Aldehyde dehydrogenase 6 family member A1 | [203] |
| *ALDOB* | ENSBTAG00000015358 | Aldolase, fructose-bisphosphate B | [203] |
| *ALG10* | ENSBTAG00000030632 | ALG10, alpha-1,2-glucosyltransferase | [140] |
| *ALG13* | ENSBTAG00000015251 | Putative bifunctional UDP-N-acetylglucosamine transferase and deubiquitinase ALG13 | [203] |
| *ALKBH1* | ENSBTAG00000008133 | AlkB homolog 1, histone H2A dioxygenase | [203] |
| *ALKBH2* | ENSBTAG00000019846 | AlkB homolog 2, alpha-ketoglutarate dependent dioxygenase | [203] |
| *ALOX12E* | ENSBTAG00000031933 | Arachidonate lipoxygenase, epidermal | [139] |
| *ALOX5AP* | ENSBTAG00000013201 | Arachidonate 5-lipoxygenase activating protein | [51] |
| *ALS2* | ENSBTAG00000007395 | Alsin Rho guanine nucleotide exchange factor ALS2 | [203] |
| *AMH* | ENSBTAG00000014955 | Anti-Mullerian hormone | [203] |
| *AMOT* | ENSBTAG00000016221 | Angiomotin | [204] |
| *AMPD2* | ENSBTAG00000013018 | Adenosine monophosphate deaminase 2 | [51] |
| *AMT* | ENSBTAG00000002321 | Aminomethyltransferase | [203] |
| *AMZ1* | ENSBTAG00000026194 | Archaelysin family metallopeptidase 1 | [203] |
| *ANGPT2* | ENSBTAG00000011034 | Angiopoietin 2 | [52] |
| *ANGPTL4* | ENSBTAG00000002473 | Angiopoietin like 4 | [139] |
| *ANKMY2* | ENSBTAG00000010980 | Ankyrin repeat and MYND domain containing 2 | [138] |
| *ANKRD1* | ENSBTAG00000011734 | Ankyrin repeat domain 1 | [139] |
| *ANKRD12* | ENSBTAG00000002755 | Ankyrin repeat domain 12 | [139] |
| *ANKRD13A* | ENSBTAG00000014376 | Ankyrin repeat domain 13A | [140] |
| *ANKRD17* | ENSBTAG00000004912 | Ankyrin repeat domain 17 | [203] |
| *ANKRD33B* | ENSBTAG00000011337 | Ankyrin repeat domain 33B | [203] |
| *ANKRD34B* | ENSBTAG00000007633 | Ankyrin repeat domain 34B | [203] |
| *ANKRD52* | ENSBTAG00000011762 | Ankyrin repeat domain 52 | [138] |
| *ANO10* | ENSBTAG00000008893 | Anoctamin 10 | [140] |
| *ANXA1* | ENSBTAG00000015978 | Annexin A1 | [140] |
| *AOAH* | ENSBTAG00000018119 | Acyloxyacyl hydrolase | [203] |
| *AP4E1* | ENSBTAG00000013616 | Adaptor related protein complex 4 subunit epsilon 1 | [203] |
| *APBB1IP* | ENSBTAG00000012526 | Amyloid beta precursor protein binding family B member 1 interacting protein | [203] |
| *APBB3* | ENSBTAG00000001450 | Amyloid beta precursor protein binding family B member 3 | [203] |
| *APCDD1* | ENSBTAG00000005154 | APC down-regulated 1 | [138] |
| *APEX1* | ENSBTAG00000002745 | Apurinic/apyrimidinic endodeoxyribonuclease 1 | [138] |
| *APLNR* | ENSBTAG00000027516 | Apelin receptor | [139] |
| *APOA5* | ENSBTAG00000019764 | Apolipoprotein A5 | [203] |
| *APOBEC3A* | ENSBTAG00000037800 | Apolipoprotein B mRNA editing enzyme, catalytic polypeptide-like 3A | [139] |
| *APOE* | ENSBTAG00000010123 | Apolipoprotein E | [52] |
| *APOF* | ENSBTAG00000004384 | Apolipoprotein F | [140] |
| *APOL3* | ENSBTAG00000000667 | Apolipoprotein L, 3 | [140] |
| *AQP12B* | ENSBTAG00000003389 | Aquaporin 12B | [203] |
| *AQP9* | ENSBTAG00000013452 | Aquaporin 9 | [51] |
| *ARAF* | ENSBTAG00000005039 | A-Raf proto-oncogene, serine/threonine kinase | [204] |
| *ARAP1* | ENSBTAG00000002051 | ArfGAP with RhoGAP domain, ankyrin repeat and PH domain 1 | [138] |
| *ARC* | ENSBTAG00000021639 | Activity regulated cytoskeleton associated protein | [138] |
| *AREL1* | ENSBTAG00000020379 | Apoptosis resistant E3 ubiquitin protein ligase 1 | [203] |
| *ARHGAP15* | ENSBTAG00000032289 | Rho GTPase activating protein 15 | [51] |
| *ARHGAP20* | ENSBTAG00000000333 | Rho GTPase activating protein 20 | [203] |
| *ARHGAP30* | ENSBTAG00000017875 | Rho GTPase activating protein 30 | [51] |
| *ARHGAP36* | ENSBTAG00000021189 | Rho GTPase activating protein 36 | [203] |
| *ARHGAP42* | ENSBTAG00000004423 | Rho GTPase activating protein 42 | [203] |
| *ARHGAP44* | ENSBTAG00000021938 | Rho GTPase activating protein 44 | [203] |
| *ARHGEF12* | ENSBTAG00000021343 | Rho guanine nucleotide exchange factor 12 | [203] |
| *ARHGEF28* | ENSBTAG00000005633 | Rho guanine nucleotide exchange factor 28 | [203] |
| *ARHGEF9* | ENSBTAG00000012729 | Cdc42 guanine nucleotide exchange factor 9 | [203] |
| *ARID3B* | ENSBTAG00000011828 | AT-rich interaction domain 3B | [203] |
| *ARID5A* | ENSBTAG00000014090 | AT-rich interaction domain 5A | [138] |
| *ARL11* | ENSBTAG00000016998 | ADP ribosylation factor like GTPase 11 | [203] |
| *ARMC4* | ENSBTAG00000000654 | Armadillo repeat containing 4 | [203] |
| *ARPC4* | ENSBTAG00000007964 | Actin related protein 2/3 complex subunit 4 | [203] |
| *ARPC5L* | ENSBTAG00000015278 | Actin related protein 2/3 complex subunit 5 like | [204] |
| *ARPIN* | ENSBTAG00000027059 | Actin related protein 2/3 complex inhibitor | [203] |
| *ARPP21* | ENSBTAG00000007732 | CAMP regulated phosphoprotein 21 | [203] |
| *ARRB1* | ENSBTAG00000020485 | Arrestin beta 1 | [51] |
| *ARRDC2* | ENSBTAG00000039335 | Arrestin domain containing 2 | [139] |
| *ARRDC4* | ENSBTAG00000018252 | Arrestin domain containing 4 | [140] |
| *ART3* | ENSBTAG00000010954 | ADP-ribosyltransferase 3 | [203] |
| *ASAP1* | ENSBTAG00000003185 | ArfGAP with SH3 domain, ankyrin repeat and PH domain 1 | [204] |
| *ASCC1* | ENSBTAG00000000392 | Activating signal cointegrator 1 complex subunit 1 | [204] |
| *ASGR2* | ENSBTAG00000025101 | Asialoglycoprotein receptor 2 | [203] |
| *ASPM* | ENSBTAG00000007860 | Assembly factor for spindle microtubules | [139] |
| *ASZ1* | ENSBTAG00000000169 | Ankyrin repeat, SAM and basic leucine zipper domain containing 1 | [203] |
| *ATAD1* | ENSBTAG00000000806 | ATPase family AAA domain containing 1 | [139] |
| *ATF3* | ENSBTAG00000008545 | Activating transcription factor 3 | [139] |
| *ATF4* | ENSBTAG00000017462 | Activating transcription factor 4 | [204] |
| *ATG4B* | ENSBTAG00000015401 | Autophagy related 4B cysteine peptidase | [203] |
| *ATM* | ENSBTAG00000003111 | ATM serine/threonine kinase | [205] |
| *ATP10D* | ENSBTAG00000000473 | ATPase phospholipid transporting 10D (putative) | [203] |
| *ATP1B3* | ENSBTAG00000014140 | ATPase Na+/K+ transporting subunit beta 3 | [138] |
| *ATP1B4* | ENSBTAG00000008920 | ATPase Na+/K+ transporting family member beta 4 | [138] |
| *ATP2A3* | ENSBTAG00000014806 | ATPase sarcoplasmic/endoplasmic reticulum Ca2+ transporting 3 | [203] |
| *ATP5B* | ENSBTAG00000013315 | ATP synthase F1 subunit beta | [140] |
| *ATP5O* | ENSBTAG00000018278 | ATP synthase peripheral stalk subunit OSCP | [138] |
| *ATP6AP1* | ENSBTAG00000012117 | ATPase H+ transporting accessory protein 1 | [203] |
| *ATP6AP2* | ENSBTAG00000017801 | ATPase H+ transporting accessory protein 2 | [140] |
| *ATP6V1A* | ENSBTAG00000002703 | ATPase H+ transporting V1 subunit A | [140] |
| *ATP6V1B1* | ENSBTAG00000010620 | ATPase H+ transporting V1 subunit B1 | [203] |
| *ATRIP* | ENSBTAG00000008399 | ATR interacting protein | [203] |
| *ATXN3* | ENSBTAG00000005786 | Ataxin 3 | [139] |
| *AURKB* | ENSBTAG00000001717 | Aurora kinase B | [140] |
| *B3GNT5* | ENSBTAG00000010397 | UDP-GlcNAc:betaGal beta-1,3-N-acetylglucosaminyltransferase 5 | [203] |
| *B4GALT1* | ENSBTAG00000015249 | Beta-1,4-galactosyltransferase 1 | [204] |
| *BABAM2* | ENSBTAG00000031335 | BRISC and BRCA1 A complex member 2 | [205] |
| *BACE2* | ENSBTAG00000000394 | Beta-secretase 2 | [51] |
| *BACH2* | ENSBTAG00000020713 | BTB domain and CNC homolog 2 | [139] |
| *BAG3* | ENSBTAG00000013641 | BAG cochaperone 3 | [138] |
| *BAG6* | ENSBTAG00000019685 | BAG cochaperone 6 | [203] |
| *BAIAP2L1* | ENSBTAG00000019181 | BAR/IMD domain containing adaptor protein 2 like 1 | [140] |
| *BAMBI* | ENSBTAG00000003045 | BMP and activin membrane bound inhibitor | [205] |
| *BAP1* | ENSBTAG00000045817 | BRCA1 associated protein 1 | [203] |
| *BASP1* | ENSBTAG00000048565 | rain abundant membrane attached signal protein 1 | [51] |
| *BAT1* | ENSBTAG00000014490 | DExD-box helicase 39B | [138] |
| *BATF* | ENSBTAG00000025405 | Basic leucine zipper ATF-like transcription factor | [203] |
| *BAX* | ENSBTAG00000013340 | BCL2 associated X, apoptosis regulator | [204] |
| *BBS10* | ENSBTAG00000050467 | Bardet-Biedl syndrome 10 | [203] |
| *BCAT1* | ENSBTAG00000013825 | Branched chain amino acid transaminase 1 | [203] |
| *BCCIP* | ENSBTAG00000005792 | BRCA2 and CDKN1A interacting protein | [140] |
| *BCHE* | ENSBTAG00000011139 | Butyrylcholinesterase | [139] |
| *BCKDHA* | ENSBTAG00000016037 | Branched chain keto acid dehydrogenase E1 subunit alpha | [51] |
| *BCL2* | ENSBTAG00000019302 | BCL2 apoptosis regulator | [52] |
| *BCL2A1* | ENSBTAG00000000735 | BCL2 related protein A1 | [51, 138] |
| *BCL6* | ENSBTAG00000001511 | BCL6 transcription repressor | [203] |
| *BDH2* | ENSBTAG00000002526 | 3-hydroxybutyrate dehydrogenase 2 | [203] |
| *BEX2* | ENSBTAG00000032875 | Brain expressed X-linked 2 | [203] |
| *BHLHE22* | ENSBTAG00000033173 | Basic helix-loop-helix family member e22 | [139] |
| *BHLHE41* | ENSBTAG00000030575 | Basic helix-loop-helix family member e41 | [139] |
| *BIK* | ENSBTAG00000049724 | BCL2-interacting killer (apoptosis-inducing) | [203] |
| *BIN1* | ENSBTAG00000019177 | Bridging integrator 1 | [203] |
| *BIRC2* | ENSBTAG00000035735 | Baculoviral IAP repeat containing 2 | [139] |
| *BIRC3* | ENSBTAG00000024918 | Baculoviral IAP repeat containing 3 | [51, 138] |
| *BIVM* | ENSBTAG00000048674 | Basic, immunoglobulin-like variable motif containing | [203] |
| *BLOC1S3* | ENSBTAG00000007070 | Biogenesis of lysosomal organelles complex 1 subunit 3 | [203] |
| *BLZF1* | ENSBTAG00000002690 | Basic leucine zipper nuclear factor 1 | [139] |
| *BMP2* | ENSBTAG00000005111 | Bone morphogenetic protein 2 | [139] |
| *BMP7* | ENSBTAG00000015362 | Bone morphogenetic protein 7 | [203] |
| *BNIP3* | ENSBTAG00000017804 | BCL2 interacting protein 3 | [139] |
| *BOLA1* | ENSBTAG00000006050 | BolA family member 1 | [203] |
| *BOLA-DOB* | ENSBTAG00000047707 | Major histocompatibility complex, class II, DO beta | [203] |
| *BOLA-DQA5* | ENSBTAG00000038128 | Major histocompatibility complex, class II, DQ alpha 5 | [203] |
| *BOSTAUV1R403* | ENSBTAG00000038986 | Vomeronasal 1 receptor bosTauV1R403 | [203] |
| *BOSTAUV1R404* | ENSBTAG00000011119 | Vomeronasal 1 receptor bosTauV1R404 | [203] |
| *BPIFB1* | ENSBTAG00000015724 | BPI fold containing family B member 1 | [203] |
| *BPIFC* | ENSBTAG00000011071 | BPI fold containing family C | [203] |
| *BRINP1* | ENSBTAG00000015936 | BMP/retinoic acid inducible neural specific 1 | [203] |
| *BRS3* | ENSBTAG00000005616 | Bombesin receptor subtype 3 | [203] |
| *BRWD1* | ENSBTAG00000013602 | Bromodomain and WD repeat domain containing 1 | [203] |
| *BTG1* | ENSBTAG00000020330 | BTG anti-proliferation factor 1 | [204] |
| *BTG3* | ENSBTAG00000018704 | BTG anti-proliferation factor 3 | [140] |
| *BTK* | ENSBTAG00000019250 | Bruton tyrosine kinase | [203] |
| *BUB1* | ENSBTAG00000021181 | BUB1 mitotic checkpoint serine/threonine kinase | [139] |
| *BUB1B* | ENSBTAG00000007237 | BUB1 mitotic checkpoint serine/threonine kinase B | [139] |
| *BVES* | ENSBTAG00000018790 | Blood vessel epicardial substance | [203] |
| *BZW2* | ENSBTAG00000014262 | Basic leucine zipper and W2 domains 2 | [204] |
| *C10H15orf48* | ENSBTAG00000004558 | Chromosome 10 C15orf48 homolog | [203] |
| *C11H2orf42* | ENSBTAG00000016939 | Chromosome 11 C2orf42 homolog | [203] |
| *C15H11orf70* | ENSBTAG00000015309 | Cilia and flagella associated protein 300 | [203] |
| *C15H11orf88* | ENSBTAG00000034338 | HOATZ cilia and flagella associated protein | [203] |
| *C15H11orf96* | ENSBTAG00000045822 | Chromosome 15 C11orf96 homolog | [203] |
| *C17H22orf25* | ENSBTAG00000012421 | Transport and golgi organization 2 homolog | [203] |
| *C17H22orf39* | ENSBTAG00000048885 | Chromosome 17 C22orf39 homolog | [203] |
| *C17H4orf45* | ENSBTAG00000033547 | Chromosome 17 C4orf45 homolog | [203] |
| *C18H16orf70* | ENSBTAG00000005058 | Chromosome 18 C16orf70 homolog | [203] |
| *C1H21orf63* | ENSBTAG00000017310 | Eva-1 homolog C | [51] |
| *C1H3orf38* | ENSBTAG00000007970 | Chromosome 1 C3orf38 homolog | [203] |
| *C1H3orf52* | ENSBTAG00000016554 | Chromosome 1 C3orf52 homolog | [203] |
| *C1orf198* | ENSBTAG00000023074 | Chromosome 28 C1orf198 homolog | [203] |
| *C1orf54* | ENSBTAG00000021485 | Chromosome 3 C1orf54 homolog | [139] |
| *C1QTNF5* | ENSBTAG00000027766 | C1q and TNF related 5 | [139] |
| *C1R* | ENSBTAG00000008612 | Complement C1r | [139] |
| *C1S* | ENSBTAG00000004840 | Complement C1s | [138] |
| *C2* | ENSBTAG00000007450 | Complement C2 | [139] |
| *C20H5orf22* | ENSBTAG00000017569 | Chromosome 20 C5orf22 homolog | [203] |
| *C2CD3* | ENSBTAG00000002907 | C2 domain containing 3 centriole elongation regulator | [203] |
| *C2CD5* | ENSBTAG00000001673 | C2 calcium dependent domain containing 5 | [203] |
| *C2ORF80* | ENSBTAG00000012888 | Chromosome 2 C2orf80 homolog | [138] |
| *C3AR1* | ENSBTAG00000019741 | Complement C3a receptor 1 | [203] |
| *C3H1orf141* | ENSBTAG00000030809 | Chromosome 3 C1orf141 homolog | [203] |
| *C3H2orf54* | ENSBTAG00000012374 | Mab-21 like 4 | [203] |
| *C4BPA* | ENSBTAG00000009876 | Complement component 4 binding protein alpha | [52] |
| *C5AR2* | ENSBTAG00000037735 | Complement component 5a receptor 2 | [203] |
| *C5H12orf54* | ENSBTAG00000016996 | Chromosome 5 C12orf54 homolog | [203] |
| *CA3* | ENSBTAG00000015214 | Carbonic anhydrase 3 | [203] |
| *CA4* | ENSBTAG00000017969 | Carbonic anhydrase 4 | [203] |
| *CA5B* | ENSBTAG00000020418 | Carbonic anhydrase 5B | [203] |
| *CA8* | ENSBTAG00000017529 | Carbonic anhydrase 8 | [203] |
| *CACHD1* | ENSBTAG00000016455 | Cache domain containing 1 | [203] |
| *CACNA1D* | ENSBTAG00000010026 | Calcium voltage-gated channel subunit alpha1 D | [203] |
| *CACNA1E* | ENSBTAG00000020887 | Calcium voltage-gated channel subunit alpha1 E | [203] |
| *CACNA2D3* | ENSBTAG00000013117 | Calcium voltage-gated channel auxiliary subunit alpha2delta 3 | [203] |
| *CACNB3* | ENSBTAG00000021808 | Calcium voltage-gated channel auxiliary subunit beta 3 | [139] |
| *CACNB4* | ENSBTAG00000002297 | Calcium voltage-gated channel auxiliary subunit beta 4 | [203] |
| *CACYBP* | ENSBTAG00000001107 | Calcyclin binding protein | [138] |
| *CALCOCO2* | ENSBTAG00000007935 | Calcium binding and coiled-coil domain 2 | [139] |
| *CALM3* | ENSBTAG00000014583 | Calmodulin 3 | [203] |
| *CALR* | ENSBTAG00000015114 | Calreticulin | [138] |
| *CAMK1D* | ENSBTAG00000008650 | Calcium/calmodulin dependent protein kinase ID | [139] |
| *CAMP* | ENSBTAG00000003022 | Cathelicidin antimicrobial peptide | [140] |
| *CAMSAP1* | ENSBTAG00000006008 | Calmodulin regulated spectrin associated protein 1 | [203] |
| *CAMSAP3* | ENSBTAG00000011356 | Calmodulin regulated spectrin associated protein family member 3 | [203] |
| *CAMTA1* | ENSBTAG00000014602 | Calmodulin binding transcription activator 1 | [203] |
| *CAPN1* | ENSBTAG00000010230 | Calpain 1 | [203] |
| *CAPN6* | ENSBTAG00000000828 | Calpain 6 | [139] |
| *CAPS* | ENSBTAG00000006069 | Calcyphosine | [203] |
| *CARF* | ENSBTAG00000016000 | Calcium responsive transcription factor | [140] |
| *CARMIL3* | ENSBTAG00000015208 | Capping protein regulator and myosin 1 linker 3 | [203] |
| *CARTPT* | ENSBTAG00000047486 | CART prepropeptide | [203] |
| *CASP3* | ENSBTAG00000015874 | Caspase 3 | [139] |
| *CASP4* | ENSBTAG00000020884 | Caspase 4, apoptosis-related cysteine peptidase | [51, 138] |
| *CASP7* | ENSBTAG00000006615 | Caspase 7 | [139] |
| *CASP8* | ENSBTAG00000015718 | Caspase 8 | [140] |
| *CASTOR2* | ENSBTAG00000014417 | Cytosolic arginine sensor for mTORC1 subunit 2 | [203] |
| *CATHL4* | ENSBTAG00000053016 | Cathelicidin 4 | [203] |
| *CATHL5* | ENSBTAG00000020076 | Cathelicidin 5 | [203] |
| *CATSPER4* | ENSBTAG00000026638 | Cation channel sperm associated 4 | [203] |
| *CAV1* | ENSBTAG00000017869 | Caveolin 1 | [138] |
| *CAVIN1* | ENSBTAG00000039684 | Caveolae associated protein 1 | [203] |
| *CBLN1* | ENSBTAG00000015194 | Cerebellin 1 precursor | [203] |
| *CBLN4* | ENSBTAG00000017524 | Cerebellin 4 precursor | [203] |
| *CBS* | ENSBTAG00000000160 | Cystathionine beta-synthase | [204] |
| *CBX1* | ENSBTAG00000002949 | Chromobox 1 | [138] |
| *CBX8* | ENSBTAG00000009107 | Chromobox 8 | [203] |
| *CBY2* | ENSBTAG00000018580 | Chibby family member 2 | [203] |
| *CBY3* | ENSBTAG00000046663 | Chibby family member 3 | [203] |
| *CCDC105* | ENSBTAG00000020615 | Coiled-coil domain containing 105 | [203] |
| *CCDC112* | ENSBTAG00000001898 | Coiled-coil domain containing 112 | [139] |
| *CCDC116* | ENSBTAG00000006173 | Coiled-coil domain containing 116 | [140] |
| *CCDC120* | ENSBTAG00000002561 | Coiled-coil domain containing 120 | [203] |
| *CCDC149* | ENSBTAG00000021940 | Coiled-coil domain containing 149 | [203] |
| *CCDC158* | ENSBTAG00000012157 | Coiled-coil domain containing 158 | [203] |
| *CCDC172* | ENSBTAG00000032299 | Coiled-coil domain containing 172 | [203] |
| *CCDC173* | ENSBTAG00000001090 | Coiled-coil domain containing 173 | [203] |
| *CCDC53* | ENSBTAG00000000021 | WASH complex subunit 3 | [204] |
| *CCDC57* | ENSBTAG00000019321 | Coiled-coil domain containing 57 | [203] |
| *CCDC68* | ENSBTAG00000008158 | Coiled-coil domain containing 68 | [203] |
| *CCDC70* | ENSBTAG00000019863 | Coiled-coil domain containing 70 | [203] |
| *CCDC80* | ENSBTAG00000010793 | Coiled-coil domain containing 80 | [52] |
| *CCDC82* | ENSBTAG00000006581 | Coiled-coil domain containing 82 | [140] |
| *CCK* | ENSBTAG00000013027 | Cholecystokinin | [203] |
| *CCL11* | ENSBTAG00000004129 | Chemokine (C-C motif) ligand 11 | [203] |
| *CCL16* | ENSBTAG00000053578 | C-C motif chemokine ligand 16 | [138] |
| *CCL19* | ENSBTAG00000012684 | C-C motif chemokine ligand 19 | [51] |
| *CCL2* | ENSBTAG00000037811 | Chemokine (C-C motif) ligand 2 | [139] |
| *CCL20* | ENSBTAG00000021326 | C-C motif chemokine ligand 20 | [139] |
| *CCL28* | ENSBTAG00000001557 | C-C motif chemokine ligand 28 | [203] |
| *CCL3* | ENSBTAG00000025250 | Chemokine (C-C motif) ligand 3 | [52] |
| *CCL4* | ENSBTAG00000025257 | C-C motif chemokine ligand 4 | [51] |
| *CCN4* | ENSBTAG00000000707 | Cellular communication network factor 4 | [203] |
| *CCNA2* | ENSBTAG00000004943 | Cyclin A2 | [140] |
| *CCNB2* | ENSBTAG00000005269 | Cyclin B2 | [139] |
| *CCNY* | ENSBTAG00000005492 | Cyclin Y | [204] |
| *CCPG1* | ENSBTAG00000012741 | Cell cycle progression 1 | [139] |
| *CCR1* | ENSBTAG00000019428 | Chemokine (C-C motif) receptor 1 | [51] |
| *CCR5* | ENSBTAG00000008099 | C-C motif chemokine receptor 5 | [52] |
| *CCR7* | ENSBTAG00000015133 | C-C motif chemokine receptor 7 | [203] |
| *CCRL2* | ENSBTAG00000006155 | C-C motif chemokine receptor like 2 | [203] |
| *CCT2* | ENSBTAG00000019156 | Chaperonin containing TCP1 subunit 2 | [140] |
| *CCT3* | ENSBTAG00000006370 | Chaperonin containing TCP1 subunit 3 | [138] |
| *CCT6A* | ENSBTAG00000008184 | Chaperonin containing TCP1 subunit 6A | [203] |
| *CCT8* | ENSBTAG00000014233 | Chaperonin containing TCP1 subunit 8 | [138] |
| *CD163* | ENSBTAG00000019669 | CD163 molecule | [138] |
| *CD163L1* | ENSBTAG00000054245 | CD163 molecule-like 1 | [203] |
| *CD1E* | ENSBTAG00000009421 | CD1e molecule | [203] |
| *CD200R1L* | ENSBTAG00000001235 | CD200 receptor 1-like | [203] |
| *CD244* | ENSBTAG00000002951 | CD244 molecule | [138] |
| *CD276* | ENSBTAG00000019734 | CD276 molecule | [139] |
| *CD300LG* | ENSBTAG00000007434 | CD300 molecule like family member g | [205] |
| *CD34* | ENSBTAG00000010562 | CD34 molecule | [203] |
| *CD36* | ENSBTAG00000017866 | CD36 molecule | [138] |
| *CD37* | ENSBTAG00000011421 | CD37 molecule | [138] |
| *CD38* | ENSBTAG00000013569 | CD38 molecule | [139] |
| *CD3E* | ENSBTAG00000015710 | CD3e molecule | [138] |
| *CD40* | ENSBTAG00000020736 | CD40 molecule | [51] |
| *CD44* | ENSBTAG00000011578 | CD44 molecule | [138] |
| *CD48* | ENSBTAG00000011238 | CD48 molecule | [51] |
| *CD53* | ENSBTAG00000006466 | CD53 molecule | [51] |
| *CD55* | ENSBTAG00000006984 | CD55 molecule (Cromer blood group) | [51] |
| *CD68* | ENSBTAG00000000133 | CD68 molecule | [51, 138] |
| *CD69* | ENSBTAG00000002135 | CD69 molecule | [51] |
| *CD74* | ENSBTAG00000015228 | CD74 molecule | [138] |
| *CD83* | ENSBTAG00000050334 | CD83 molecule | [139] |
| *CD8A* | ENSBTAG00000021141 | CD8a molecule | [205] |
| *CD8B* | ENSBTAG00000008956 | CD8b molecule | [140] |
| *CD93* | ENSBTAG00000004207 | CD93 molecule | [138] |
| *CD96* | ENSBTAG00000014441 | CD96 molecule | [140] |
| *CDA* | ENSBTAG00000052516 | Cytidine deaminase | [203] |
| *CDADC1* | ENSBTAG00000002271 | Cytidine and dCMP deaminase domain containing 1 | [140] |
| *CDC20* | ENSBTAG00000009819 | Cell division cycle 20 | [139] |
| *CDCA3* | ENSBTAG00000019777 | Cell division cycle associated 3 | [139] |
| *CDCA8* | ENSBTAG00000014326 | Cell division cycle associated 8 | [139] |
| *CDH13* | ENSBTAG00000034373 | Cadherin 13 | [203] |
| *CDH26* | ENSBTAG00000020261 | Cadherin 26 | [203] |
| *CDH5* | ENSBTAG00000007421 | Cadherin 5 | [203] |
| *CDH9* | ENSBTAG00000018234 | Cadherin 9 | [203] |
| *CDHR1* | ENSBTAG00000001231 | Cadherin related family member 1 | [203] |
| *CDK1* | ENSBTAG00000010109 | Cyclin dependent kinase 1 | [139] |
| *CDK19* | ENSBTAG00000007288 | Cyclin dependent kinase 19 | [203] |
| *CDK2AP1* | ENSBTAG00000020504 | Cyclin dependent kinase 2 associated protein 1 | [203] |
| *CDK6* | ENSBTAG00000044023 | Cyclin dependent kinase 6 | [203] |
| *CDK7* | ENSBTAG00000011046 | Cyclin dependent kinase 7 | [203] |
| *CDKN2B* | ENSBTAG00000051567 | Cyclin dependent kinase inhibitor 2B | [140] |
| *CDKN2C* | ENSBTAG00000011059 | Cyclin dependent kinase inhibitor 2C | [139] |
| *CEBPB* | ENSBTAG00000051972 | CCAAT enhancer binding protein beta | [51] |
| *CEBPD* | ENSBTAG00000046307 | CCAAT enhancer binding protein delta | [140] |
| *CEBPG* | ENSBTAG00000017560 | CCAAT enhancer binding protein gamma | [204] |
| *CEBPZ* | ENSBTAG00000039770 | CCAAT enhancer binding protein zeta | [138] |
| *CEMIP2* | ENSBTAG00000005090 | Cell migration inducing hyaluronidase 2 | [203] |
| *CENPE* | ENSBTAG00000009035 | Centromere protein E | [203] |
| *CENPH* | ENSBTAG00000014246 | Centromere protein H | [203] |
| *CEP89* | ENSBTAG00000004864 | Centrosomal protein 89 | [203] |
| *CEP97* | ENSBTAG00000013464 | Centrosomal protein 97 | [203] |
| *CEPT1* | ENSBTAG00000020169 | Choline/ethanolamine phosphotransferase 1 | [203] |
| *CES1* | ENSBTAG00000011021 | Carboxylesterase 1 (monocyte/macrophage serine esterase 1) | [203] |
| *CFAP94* | ENSBTAG00000002972 | Cilia and flagella associated protein 94 | [203] |
| *CFB* | ENSBTAG00000046158 | Complement factor B | [138] |
| *CFD* | ENSBTAG00000048122 | Complement factor D | [204] |
| *CFH* | ENSBTAG00000023177 | Complement factor H | [139] |
| *CFL2* | ENSBTAG00000015053 | Cofilin 2 | [140] |
| *CFP* | ENSBTAG00000015815 | Complement factor properdin | [51] |
| *CFTR* | ENSBTAG00000006589 | CF transmembrane conductance regulator | [203] |
| *CGN* | ENSBTAG00000017564 | Cingulin | [203] |
| *CHAF1B* | ENSBTAG00000011880 | Chromatin assembly factor 1 subunit B | [203] |
| *CHAT* | ENSBTAG00000016814 | Choline O-acetyltransferase | [203] |
| *CHGB* | ENSBTAG00000011782 | Chromogranin B | [203] |
| *CHI3L1* | ENSBTAG00000018223 | Chitinase 3 like 1 | [138] |
| *CHI3L2* | ENSBTAG00000013578 | Chitinase 3 like 2 | [204] |
| *CHORDC1* | ENSBTAG00000013615 | Cysteine and histidine rich domain containing 1 | [138] |
| *CHRNA1* | ENSBTAG00000018253 | Cholinergic receptor nicotinic alpha 1 subunit | [203] |
| *CHST1* | ENSBTAG00000003721 | Carbohydrate sulfotransferase 1 | [139] |
| *CHST4* | ENSBTAG00000016968 | Carbohydrate sulfotransferase 4 | [203] |
| *CHSY1* | ENSBTAG00000007357 | Chondroitin sulfate synthase 1 | [203] |
| *CHTOP* | ENSBTAG00000005166 | Chromatin target of PRMT1 | [203] |
| *CIB3* | ENSBTAG00000016179 | Calcium and integrin binding family member 3 | [203] |
| *CIPC* | ENSBTAG00000009923 | CLOCK interacting pacemaker | [203] |
| *CIST1* | ENSBTAG00000033886 | Intestine-specific transcript 1 protein | [203] |
| *CIT* | ENSBTAG00000008963 | Citron rho-interacting serine/threonine kinase | [139] |
| *CITED1* | ENSBTAG00000045925 | Cbp/p300 interacting transactivator with Glu/Asp rich carboxy-terminal domain 1 | [203] |
| *CITED2* | ENSBTAG00000011224 | Cbp/p300 interacting transactivator with Glu/Asp rich carboxy-terminal domain 2 | [140] |
| *CLASP1* | ENSBTAG00000019781 | Cytoplasmic linker associated protein 1 | [203] |
| *CLASP2* | ENSBTAG00000011333 | Cytoplasmic linker associated protein 2 | [203] |
| *CLCA2* | ENSBTAG00000038215 | Chloride channel accessory 2 | [139] |
| *CLCC1* | ENSBTAG00000017129 | Chloride channel CLIC like 1 | [138] |
| *CLCN4* | ENSBTAG00000011121 | Chloride voltage-gated channel 4 | [138] |
| *CLDN1* | ENSBTAG00000013148 | Claudin 1 | [139] |
| *CLDN11* | ENSBTAG00000019060 | Claudin 11 | [139] |
| *CLIC1* | ENSBTAG00000013533 | Chloride intracellular channel 1 | [51] |
| *CLIC2* | ENSBTAG00000010948 | Chloride intracellular channel 2 | [203] |
| *CLIC4* | ENSBTAG00000009470 | Chloride intracellular channel 4 | [138] |
| *CLK2* | ENSBTAG00000014393 | CDC like kinase 2 | [140] |
| *CLMN* | ENSBTAG00000019916 | Calmin | [138] |
| *CLOCK* | ENSBTAG00000044044 | Clock circadian regulator | [203] |
| *CLPTM1* | ENSBTAG00000020560 | CLPTM1 regulator of GABA type A receptor forward trafficking | [203] |
| *CLPTM1L* | ENSBTAG00000001329 | CLPTM1 like | [140] |
| *CLRN3* | ENSBTAG00000047054 | Clarin 3 | [203] |
| *CMBL* | ENSBTAG00000010801 | Carboxymethylenebutenolidase homolog | [203] |
| *CMTM5* | ENSBTAG00000032709 | CKLF like MARVEL transmembrane domain containing 5 | [138] |
| *CMTM6* | ENSBTAG00000019526 | CKLF like MARVEL transmembrane domain containing 6 | [138] |
| *CMTM8* | ENSBTAG00000001710 | CKLF like MARVEL transmembrane domain containing 8 | [52] |
| *CMTR2* | ENSBTAG00000047367 | Cap methyltransferase 2 | [203] |
| *CNGB1* | ENSBTAG00000005702 | Cyclic nucleotide gated channel subunit beta 1 | [203] |
| *CNIH3* | ENSBTAG00000053199 | Cornichon family AMPA receptor auxiliary protein 3 | [138] |
| *CNKSR1* | ENSBTAG00000019399 | Connector enhancer of kinase suppressor of Ras 1 | [203] |
| *CNKSR2* | ENSBTAG00000044202 | Connector enhancer of kinase suppressor of Ras 2 | [203] |
| *CNN1* | ENSBTAG00000011207 | Calponin 1 | [138] |
| *CNNM1* | ENSBTAG00000011956 | Cyclin and CBS domain divalent metal cation transport mediator 1 | [203] |
| *CNP* | ENSBTAG00000025762 | 2,3,-cyclic nucleotide 3, phosphodiesterase | [140] |
| *CNPY3* | ENSBTAG00000015900 | Canopy FGF signaling regulator 3 | [138] |
| *CNR1* | ENSBTAG00000054523 | Cannabinoid receptor 1 | [203] |
| *CNTN3* | ENSBTAG00000007615 | Contactin 3 | [203] |
| *COG2* | ENSBTAG00000020314 | Component of oligomeric golgi complex 2 | [138] |
| *COL17A1* | ENSBTAG00000002430 | Collagen type XVII alpha 1 chain | [52] |
| *COL1A2* | ENSBTAG00000013472 | Collagen type I alpha 2 chain | [52] |
| *COL3A1* | ENSBTAG00000021466 | Collagen type III alpha 1 chain | [204] |
| *COL4A3* | ENSBTAG00000021316 | Collagen type IV alpha 3 chain | [203] |
| *COL4A5* | ENSBTAG00000014575 | Collagen type IV alpha 5 chain | [203] |
| *COL5A3* | ENSBTAG00000010179 | Collagen type V alpha 3 chain | [203] |
| *COL6A6* | ENSBTAG00000012837 | Collagen type VI alpha 6 chain | [203] |
| *COMMD10* | ENSBTAG00000019071 | COMM domain containing 10 | [203] |
| *COP1* | ENSBTAG00000031385 | COP1 E3 ubiquitin ligase | [203] |
| *COPS2* | ENSBTAG00000013425 | COP9 signalosome subunit 2 | [139] |
| *COQ6* | ENSBTAG00000020331 | Coenzyme Q6, monooxygenase | [203] |
| *CORO1A* | ENSBTAG00000008631 | Coronin 1A | [51] |
| *CORO7* | ENSBTAG00000009201 | Coronin 7 | [203] |
| *COTL1* | ENSBTAG00000016315 | Coactosin like F-actin binding protein 1 | [138] |
| *COX18* | ENSBTAG00000005394 | Cytochrome c oxidase assembly factor COX18 | [203] |
| *COX7A1* | ENSBTAG00000014878 | Cytochrome c oxidase subunit 7A1 | [138] |
| *CP* | ENSBTAG00000012164 | Ceruloplasmin | [138] |
| *CPNE8* | ENSBTAG00000020914 | Copine 8 | [139] |
| *CPOX* | ENSBTAG00000004124 | Coproporphyrinogen oxidase | [139] |
| *CRABP1* | ENSBTAG00000012909 | Cellular retinoic acid binding protein 1 | [139] |
| *CRABP2* | ENSBTAG00000005716 | Cellular retinoic acid binding protein 2 | [203] |
| *CREB3L1* | ENSBTAG00000006143 | CAMP responsive element binding protein 3 like 1 | [51] |
| *CRISPLD2* | ENSBTAG00000021176 | Cysteine rich secretory protein LCCL domain containing 2 | [52] |
| *CRLF3* | ENSBTAG00000018381 | Cytokine receptor like factor 3 | [138] |
| *CRSP9* | ENSBTAG00000050263 | Mediator complex subunit 7 | [140] |
| *CRYAA* | ENSBTAG00000003134 | Crystallin alpha A | [203] |
| *CRYBA2* | ENSBTAG00000014819 | Crystallin beta A2 | [203] |
| *CRYM* | ENSBTAG00000009842 | Crystallin mu | [139] |
| *CSF2* | ENSBTAG00000001570 | Colony stimulating factor 2 | [204] |
| *CSF2RA* | ENSBTAG00000045948 | Colony stimulating factor 2 receptor subunit alpha | [51] |
| *CSF2RB* | ENSBTAG00000009064 | Colony stimulating factor 2 receptor subunit beta | [203] |
| *CSF3* | ENSBTAG00000021462 | Colony stimulating factor 3 | [203] |
| *CSN2* | ENSBTAG00000002632 | Casein beta | [203] |
| *CSRNP3* | ENSBTAG00000039091 | Cysteine and serine rich nuclear protein 3 | [203] |
| *CSRP3* | ENSBTAG00000011869 | Cysteine and glycine rich protein 3 | [138] |
| *CTBP2* | ENSBTAG00000003397 | C-terminal binding protein 2 | [203] |
| *CSTF2* | ENSBTAG00000003547 | Cleavage stimulation factor subunit 2 | [140] |
| *CTGF* | ENSBTAG00000006367 | Cellular communication network factor 2 | [140] |
| *CTH* | ENSBTAG00000014791 | Cystathionine gamma-lyase | [140] |
| *CTNNB1* | ENSBTAG00000016420 | Catenin beta 1 | [140] |
| *CTSC* | ENSBTAG00000011100 | Cathepsin C | [51] |
| *CTSD* | ENSBTAG00000007622 | Cathepsin D | [204] |
| *CTSH* | ENSBTAG00000010992 | Cathepsin H | [139] |
| *CTSK* | ENSBTAG00000021035 | Cathepsin K | [139] |
| *CTSS* | ENSBTAG00000017135 | Cathepsin S | [51] |
| *CUL4B* | ENSBTAG00000018569 | Cullin 4B | [139] |
| *CUL5* | ENSBTAG00000012882 | Cullin 5 | [140] |
| *CWC22* | ENSBTAG00000021726 | CWC22 spliceosome associated protein homolog | [139] |
| *CX3CL1* | ENSBTAG00000024869 | C-X3-C motif chemokine ligand 1 | [139] |
| *CXCL10* | ENSBTAG00000001725 | C-X-C motif chemokine ligand 10 | [139] |
| *CXCL11* | ENSBTAG00000005603 | C-X-C motif chemokine ligand 11 | [52] |
| *CXCL13* | ENSBTAG00000008479 | C-X-C motif chemokine ligand 13 | [52] |
| *CXCL16* | ENSBTAG00000031998 | C-X-C motif chemokine ligand 16 | [138] |
| *CXCL2* | ENSBTAG00000027513 | Chemokine (C-X-C motif) ligand 2 | [51] |
| *CXCL3* | ENSBTAG00000037778 | Chemokine (C-X-C motif) ligand 3 | [139] |
| *CXCL5* | ENSBTAG00000009812 | Chemokine (C-X-C motif) ligand 5 | [51] |
| *CXCR4* | ENSBTAG00000001060 | C-X-C motif chemokine receptor 4 | [139] |
| *CYB561* | ENSBTAG00000020810 | Cytochrome b561 | [138] |
| *CYB5R1* | ENSBTAG00000019927 | Cytochrome b5 reductase 1 | [140] |
| *CYB5R4* | ENSBTAG00000013591 | Cytochrome b5 reductase 4 | [140] |
| *CYBA* | ENSBTAG00000051907 | Cytochrome b-245 alpha chain | [51] |
| *CYBB* | ENSBTAG00000019953 | Cytochrome b-245 beta chain | [51] |
| *CYP11A1* | ENSBTAG00000006934 | Cytochrome P450, family 11, subfamily A, polypeptide 1 | [139] |
| *CYP2A13* | ENSBTAG00000047815 | Cytochrome P450 2A13 | [203] |
| *CYP2C18* | ENSBTAG00000037559 | Cytochrome P450, family 2, subfamily C, polypeptide 18 | [203] |
| *CYP2C87* | ENSBTAG00000037795 | Cytochrome P450, family 2, subfamily C, polypeptide 87 | [203] |
| *CYP2E1* | ENSBTAG00000018365 | Cytochrome P450, family 2, subfamily E, polypeptide 1 | [140] |
| *CYP2J2* | ENSBTAG00000051030 | Cytochrome P450, family 2, subfamily J, polypeptide 2 | [139] |
| *CYP3A4* | ENSBTAG00000052665 | Cytochrome P450, subfamily IIIA, polypeptide 4 | [203] |
| *CYP3A5* | ENSBTAG00000053645 | Cytochrome P450, family 3, subfamily A, polypeptide 5 | [140] |
| *CYP7B1* | ENSBTAG00000001299 | Cytochrome P450, family 7, subfamily B, polypeptide 1 | [139] |
| *DAAM2* | ENSBTAG00000021381 | Dishevelled associated activator of morphogenesis 2 | [203] |
| *DAB1* | ENSBTAG00000013802 | DAB adaptor protein 1 | [139] |
| *DACT3* | ENSBTAG00000030705 | Dishevelled binding antagonist of beta catenin 3 | [204] |
| *DAGLB* | ENSBTAG00000009236 | Diacylglycerol lipase beta | [51] |
| *DAP* | ENSBTAG00000006346 | Death associated protein | [203] |
| *DAXX* | ENSBTAG00000016830 | Death domain associated protein | [140] |
| *DBNL* | ENSBTAG00000017547 | Drebrin like | [140] |
| *DCAF11* | ENSBTAG00000021392 | DDB1 and CUL4 associated factor 11 | [203] |
| *DCDC2C* | ENSBTAG00000038489 | Doublecortin domain containing 2C | [203] |
| *DDA1* | ENSBTAG00000017068 | DET1 and DDB1 associated 1 | [203] |
| *DDIT4L* | ENSBTAG00000010610 | DNA damage inducible transcript 4 like | [203] |
| *DDX31* | ENSBTAG00000003530 | DEAD-box helicase 31 | [203] |
| *DDX41* | ENSBTAG00000015187 | DEAD-box helicase 41 | [138] |
| *DDX42* | ENSBTAG00000021058 | DEAD-box helicase 42 | [140] |
| *DDX52* | ENSBTAG00000010313 | DExD-box helicase 52 | [139] |
| *DDX58* | ENSBTAG00000003366 | DExD/H-box helicase 58 | [139] |
| *DEF6* | ENSBTAG00000025622 | DEF6 guanine nucleotide exchange factor | [203] |
| *DEFB10* | ENSBTAG00000048737 | Beta-defensin 10 | [52] |
| *DENND10* | ENSBTAG00000008729 | DENN domain containing 10 | [203] |
| *DENND5A* | ENSBTAG00000006322 | DENN domain containing 5A | [203] |
| *DEPDC7* | ENSBTAG00000021809 | DEP domain containing 7 | [203] |
| *DEPP1* | ENSBTAG00000002670 | DEPP1 autophagy regulator | [203] |
| *DGAT2* | ENSBTAG00000016355 | Diacylglycerol O-acyltransferase 2 | [51] |
| *DGKE* | ENSBTAG00000004449 | Diacylglycerol kinase epsilon | [203] |
| *DHRS1* | ENSBTAG00000010234 | Dehydrogenase/reductase 1 | [204] |
| *DHRS12* | ENSBTAG00000016109 | Dehydrogenase/reductase 12 | [203] |
| *DHRS3* | ENSBTAG00000024493 | Dehydrogenase/reductase 3 | [51] |
| *DHX32* | ENSBTAG00000005795 | DEAH-box helicase 32 (putative) | [203] |
| *DHX38* | ENSBTAG00000013265 | DEAH-box helicase 38 | [203] |
| *DHX58* | ENSBTAG00000046580 | DExH-box helicase 58 | [203] |
| *DIRAS3* | ENSBTAG00000012182 | DIRAS family GTPase 3 | [203] |
| *DIS3* | ENSBTAG00000019889 | DIS3 homolog, exosome endoribonuclease and 3,-5, exoribonuclease | [203] |
| *DISP3* | ENSBTAG00000015533 | Dispatched RND transporter family member 3 | [203] |
| *DKC1* | ENSBTAG00000013045 | Dyskerin pseudouridine synthase 1 | [138] |
| *DKK2* | ENSBTAG00000012969 | Dickkopf WNT signaling pathway inhibitor 2 | [203] |
| *DLGAP3* | ENSBTAG00000013867 | DLG associated protein 3 | [203] |
| *DLST* | ENSBTAG00000006463 | Dihydrolipoamide S-succinyltransferase | [203] |
| *DMC1* | ENSBTAG00000005936 | DNA meiotic recombinase 1 | [203] |
| *DNAH7* | ENSBTAG00000016784 | Dynein axonemal heavy chain 7 | [203] |
| *DNAJB4* | ENSBTAG00000021752 | DnaJ heat shock protein family (Hsp40) member B4 | [203] |
| *DNAJC12* | ENSBTAG00000010932 | DnaJ heat shock protein family (Hsp40) member C12 | [138] |
| *DNAJC13* | ENSBTAG00000002966 | DnaJ heat shock protein family (Hsp40) member C13 | [140] |
| *DNAJC17* | ENSBTAG00000013480 | DnaJ heat shock protein family (Hsp40) member C17 | [140] |
| *DNAJC18* | ENSBTAG00000002286 | DnaJ heat shock protein family (Hsp40) member C18 | [203] |
| *DNAJC24* | ENSBTAG00000050888 | DnaJ heat shock protein family (Hsp40) member C24 | [140] |
| *DNAJC6* | ENSBTAG00000030749 | DnaJ heat shock protein family (Hsp40) member C6 | [203] |
| *DNAL1* | ENSBTAG00000015930 | Dynein axonemal light chain 1 | [203] |
| *DNASE1L1* | ENSBTAG00000007455 | Deoxyribonuclease 1 like 1 | [203] |
| *DOCK8* | ENSBTAG00000002190 | Dedicator of cytokinesis 8 | [140] |
| *DPF2* | ENSBTAG00000003077 | Double PHD fingers 2 | [140] |
| *DPP3* | ENSBTAG00000022242 | Dipeptidyl peptidase 3 | [51] |
| *DRAM1* | ENSBTAG00000017287 | DNA damage regulated autophagy modulator 1 | [139] |
| *DROSHA* | ENSBTAG00000017551 | Drosha ribonuclease III | [203] |
| *DTWD2* | ENSBTAG00000002143 | DTW domain containing 2 | [203] |
| *DTX3L* | ENSBTAG00000009933 | Deltex E3 ubiquitin ligase 3L | [139] |
| *DUS3L* | ENSBTAG00000011842 | Dihydrouridine synthase 3 like | [203] |
| *DUS4L* | ENSBTAG00000021849 | Dihydrouridine synthase 4 like | [203] |
| *DUSP1* | ENSBTAG00000013863 | Dual specificity phosphatase 1 | [139] |
| *DUSP23* | ENSBTAG00000004776 | Dual specificity phosphatase 23 | [203] |
| *DUSP8* | ENSBTAG00000051211 | Dual specificity phosphatase 8 | [203] |
| *DXO* | ENSBTAG00000005588 | Decapping exoribonuclease | [203] |
| *DYNC1I1* | ENSBTAG00000027134 | Dynein cytoplasmic 1 intermediate chain 1 | [203] |
| *DYNC2LI1* | ENSBTAG00000013676 | Dynein cytoplasmic 2 light intermediate chain 1 | [203] |
| *DYNLRB1* | ENSBTAG00000006134 | Dynein light chain roadblock-type 1 | [203] |
| *DYRK1A* | ENSBTAG00000001771 | Dual specificity tyrosine phosphorylation regulated kinase 1A | [204] |
| *DYSF* | ENSBTAG00000013290 | Dysferlin | [203] |
| *E2F8* | ENSBTAG00000017446 | E2F transcription factor 8 | [139] |
| *EBPL* | ENSBTAG00000018410 | EBP like | [203] |
| *ECM1* | ENSBTAG00000003806 | Extracellular matrix protein 1 | [52] |
| *ECM2* | ENSBTAG00000024081 | Extracellular matrix protein 2 | [139] |
| *EDEM1* | ENSBTAG00000012904 | ER degradation enhancing alpha-mannosidase like protein 1 | [203] |
| *EDN1* | ENSBTAG00000008096 | Endothelin 1 | [139] |
| *EDNRB* | ENSBTAG00000005299 | Endothelin receptor type B | [203] |
| *EEA1* | ENSBTAG00000000421 | Early endosome antigen 1 | [139] |
| *EED* | ENSBTAG00000007847 | Embryonic ectoderm development | [140] |
| *EEPD1* | ENSBTAG00000019065 | Endonuclease/exonuclease/phosphatase family domain containing 1 | [203] |
| *EFCAB6* | ENSBTAG00000030189 | EF-hand calcium binding domain 6 | [203] |
| *EFHC1* | ENSBTAG00000017810 | EF-hand domain containing 1 | [51] |
| *EFNA1* | ENSBTAG00000020244 | Ephrin A1 | [140] |
| *EGFLAM* | ENSBTAG00000019595 | EGF like, fibronectin type III and laminin G domains | [52] |
| *EGLN3* | ENSBTAG00000008172 | Egl-9 family hypoxia inducible factor 3 | [140] |
| *EGR1* | ENSBTAG00000010069 | Early growth response 1 | [138] |
| *EHBP1L1* | ENSBTAG00000009554 | EH domain binding protein 1 like 1 | [203] |
| *EHD1* | ENSBTAG00000050712 | EH domain containing 1 | [203] |
| *EHMT2* | ENSBTAG00000005676 | Euchromatic histone lysine methyltransferase 2 | [203] |
| *EI24* | ENSBTAG00000007202 | EI24 autophagy associated transmembrane protein | [140] |
| *EIF1* | ENSBTAG00000002282 | Eukaryotic translation initiation factor 1 | [138] |
| *EIF1B* | ENSBTAG00000019106 | Eukaryotic translation initiation factor 1B | [204] |
| *EIF2AK2* | ENSBTAG00000008703 | Eukaryotic translation initiation factor 2 alpha kinase 2 | [139] |
| *EIF2B2* | ENSBTAG00000008664 | Eukaryotic translation initiation factor 2B subunit beta | [203] |
| *EIF3A* | ENSBTAG00000002528 | Eukaryotic translation initiation factor 3 subunit A | [203] |
| *EIF3D* | ENSBTAG00000001988 | Eukaryotic translation initiation factor 3 subunit D | [204] |
| *EIF4A1* | ENSBTAG00000000132 | Eukaryotic translation initiation factor 4A1 | [138] |
| *EIF4A2* | ENSBTAG00000014724 | Eukaryotic translation initiation factor 4A2 | [204] |
| *EIF4E* | ENSBTAG00000009522 | Eukaryotic translation initiation factor 4E | [139] |
| *EIF4E3* | ENSBTAG00000018862 | Eukaryotic translation initiation factor 4E family member 3 | [140] |
| *EIF4EBP1* | ENSBTAG00000027654 | Eukaryotic translation initiation factor 4E binding protein 1 | [140] |
| *EIF4G2* | ENSBTAG00000020308 | Eukaryotic translation initiation factor 4 gamma 2 | [140] |
| *ELAVL3* | ENSBTAG00000014412 | ELAV like RNA binding protein 3 | [203] |
| *ELF2* | ENSBTAG00000020594 | E74 like ETS transcription factor 2 | [203] |
| *ELL3* | ENSBTAG00000032132 | Elongation factor for RNA polymerase II 3 | [138] |
| *ELOC* | ENSBTAG00000050514 | Elongin C | [203] |
| *ELP1* | ENSBTAG00000004991 | Elongator acetyltransferase complex subunit 1 | [203] |
| *EMC1* | ENSBTAG00000011950 | ER membrane protein complex subunit 1 | [203] |
| *EMC3* | ENSBTAG00000003882 | ER membrane protein complex subunit 3 | [203] |
| *EMC6* | ENSBTAG00000025121 | ER membrane protein complex subunit 6 | [203] |
| *EMILIN2* | ENSBTAG00000003880 | Elastin microfibril interfacer 2 | [203] |
| *EML3* | ENSBTAG00000011872 | EMAP like 3 | [203] |
| *EMP1* | ENSBTAG00000036078 | Epithelial membrane protein 1 | [204] |
| *EMX2* | ENSBTAG00000003027 | Empty spiracles homeobox 2 | [203] |
| *ENC1* | ENSBTAG00000026369 | Ectodermal-neural cortex 1 | [138] |
| *ENDOD1* | ENSBTAG00000024803 | Endonuclease domain containing 1 | [203] |
| *ENPP1* | ENSBTAG00000021830 | Ectonucleotide pyrophosphatase/phosphodiesterase 1 | [203] |
| *ENTPD1* | ENSBTAG00000020589 | Ectonucleoside triphosphate diphosphohydrolase 1 | [140] |
| *ENTPD5* | ENSBTAG00000020334 | Ectonucleoside triphosphate diphosphohydrolase 5 (inactive) | [203] |
| *ENTPD8* | ENSBTAG00000030246 | Ectonucleoside triphosphate diphosphohydrolase 8 | [203] |
| *EOMES* | ENSBTAG00000016920 | Eomesodermin | [203] |
| *EPAS1* | ENSBTAG00000003711 | Endothelial PAS domain protein 1 | [138] |
| *EPB41L1* | ENSBTAG00000001640 | Erythrocyte membrane protein band 4.1 like 1 | [203] |
| *EPB41L5* | ENSBTAG00000018236 | Erythrocyte membrane protein band 4.1 like 5 | [203] |
| *EPC2* | ENSBTAG00000018581 | Enhancer of polycomb homolog 2 | [139] |
| *EPCAM* | ENSBTAG00000006474 | Epithelial cell adhesion molecule | [203] |
| *EPHA2* | ENSBTAG00000000815 | EPH receptor A2 | [140] |
| *EPHB1* | ENSBTAG00000015377 | EPH receptor B1 | [203] |
| *EPHX2* | ENSBTAG00000015241 | Epoxide hydrolase 2 | [139] |
| *EPM2A* | ENSBTAG00000015181 | EPM2A glucan phosphatase, laforin | [203] |
| *EPS8* | ENSBTAG00000000369 | Epidermal growth factor receptor pathway substrate 8 | [203] |
| *EPS8L2* | ENSBTAG00000022147 | EPS8 like 2 | [203] |
| *EPSTI1* | ENSBTAG00000019054 | Epithelial stromal interaction 1 | [140] |
| *ERAP1* | ENSBTAG00000013557 | Endoplasmic reticulum aminopeptidase 1 | [205] |
| *ERAP2* | ENSBTAG00000039275 | Endoplasmic reticulum aminopeptidase 2 | [203] |
| *ERCC5* | ENSBTAG00000014043 | ERCC excision repair 5, endonuclease | [138] |
| *ERG28* | ENSBTAG00000011987 | Ergosterol biosynthesis 28 homolog | [203] |
| *ERLIN1* | ENSBTAG00000007588 | ER lipid raft associated 1 | [138] |
| *ERO1A* | ENSBTAG00000015716 | Endoplasmic reticulum oxidoreductase 1 alpha | [203] |
| *ESCO1* | ENSBTAG00000017597 | Establishment of sister chromatid cohesion N-acetyltransferase 1 | [139] |
| *ESF1* | ENSBTAG00000007130 | ESF1 nucleolar pre-rRNA processing protein homolog | [138] |
| *ESRRG* | ENSBTAG00000010392 | Estrogen related receptor gamma | [203] |
| *ETAA1* | ENSBTAG00000011727 | ETAA1 activator of ATR kinase | [139] |
| *ETFA* | ENSBTAG00000012490 | Electron transfer flavoprotein subunit alpha | [140] |
| *ETFRF1* | ENSBTAG00000026624 | Electron transfer flavoprotein regulatory factor 1 | [203] |
| *ETS2* | ENSBTAG00000009214 | ETS proto-oncogene 2, transcription factor | [51] |
| *EVX1* | ENSBTAG00000020919 | Even-skipped homeobox 1 | [203] |
| *EXOC3L1* | ENSBTAG00000012035 | Exocyst complex component 3 like 1 | [203] |
| *EXOC4* | ENSBTAG00000014112 | Exocyst complex component 4 | [205] |
| *EXOC8* | ENSBTAG00000006111 | Exocyst complex component 8 | [203] |
| *EXOSC5* | ENSBTAG00000016034 | Exosome component 5 | [138] |
| *EXPH5* | ENSBTAG00000008253 | Exophilin 5 | [203] |
| *EXTL1* | ENSBTAG00000006349 | Exostosin like glycosyltransferase 1 | [203] |
| *F13A1* | ENSBTAG00000007268 | Coagulation factor XIII A chain | [51] |
| *F2RL2* | ENSBTAG00000000898 | Coagulation factor II thrombin receptor like 2 | [139] |
| *F5* | ENSBTAG00000017722 | Coagulation factor V | [51] |
| *FA2H* | ENSBTAG00000012222 | Fatty acid 2-hydroxylase | [203] |
| *FAH* | ENSBTAG00000012338 | Fumarylacetoacetate hydrolase | [203] |
| *FAM102A* | ENSBTAG00000023851 | Family with sequence similarity 102 member A | [203] |
| *FAM114A1* | ENSBTAG00000033160 | Family with sequence similarity 114 member A1 | [140] |
| *FAM120A* | ENSBTAG00000008696 | Family with sequence similarity 120A | [140] |
| *FAM149B1* | ENSBTAG00000019130 | Family with sequence similarity 149 member B1 | [203] |
| *FAM160B2* | ENSBTAG00000014361 | Family with sequence similarity 160 member B2 | [203] |
| *FAM162B* | ENSBTAG00000033460 | Family with sequence similarity 162 member B | [203] |
| *FAM170A* | ENSBTAG00000003047 | Family with sequence similarity 170 member A | [203] |
| *FAM174B* | ENSBTAG00000022989 | Family with sequence similarity 174 member B | [138] |
| *FAM204A* | ENSBTAG00000005057 | Family with sequence similarity 204 member A | [203] |
| *FAM234B* | ENSBTAG00000014322 | Family with sequence similarity 234 member B | [203] |
| *FAM3C* | ENSBTAG00000007976 | FAM3 metabolism regulating signaling molecule C | [140] |
| *FAM43A* | ENSBTAG00000008462 | Family with sequence similarity 43 member A | [139] |
| *FAM49B* | ENSBTAG00000020801 | CYFIP related Rac1 interactor B | [51] |
| *FAM53C* | ENSBTAG00000030529 | Family with sequence similarity 53 member C | [138] |
| *FAM69C* | ENSBTAG00000000106 | Divergent protein kinase domain 1C | [138] |
| *FAM83D* | ENSBTAG00000000660 | Family with sequence similarity 83 member D | [139] |
| *FANK1* | ENSBTAG00000022917 | Fibronectin type III and ankyrin repeat domains 1 | [203] |
| *FAP* | ENSBTAG00000008140 | Fibroblast activation protein alpha | [139] |
| *FAS* | ENSBTAG00000010785 | Fas cell surface death receptor | [140] |
| *FASN* | ENSBTAG00000015980 | Fatty acid synthase | [51] |
| *FASTKD3* | ENSBTAG00000009400 | FAST kinase domains 3 | [203] |
| *FBL* | ENSBTAG00000002579 | Fibrillarin | [138] |
| *FBLN1* | ENSBTAG00000012088 | Fibulin 1 | [138] |
| *FBXL3* | ENSBTAG00000018848 | F-box and leucine rich repeat protein 3 | [138] |
| *FBXO10* | ENSBTAG00000012262 | F-box protein 10 | [203] |
| *FBXO30* | ENSBTAG00000005376 | F-box protein 30 | [139] |
| *FBXO4* | ENSBTAG00000016981 | F-box protein 4 | [203] |
| *FBXO45* | ENSBTAG00000006388 | F-box protein 45 | [203] |
| *FBXO47* | ENSBTAG00000024983 | F-box protein 47 | [203] |
| *FBXO5* | ENSBTAG00000021193 | F-box protein 5 | [139] |
| *FBXW8* | ENSBTAG00000036183 | F-box and WD repeat domain containing 8 | [140] |
| *FCAR* | ENSBTAG00000021647 | Fc fragment of IgA receptor | [51] |
| *FCER1G* | ENSBTAG00000024503 | Fc fragment of IgE receptor Ig | [138] |
| *FCF1* | ENSBTAG00000020381 | FCF1 rRNA-processing protein | [203] |
| *FCGR2A* | ENSBTAG00000038124 | Fc fragment of IgG receptor IIa | [204] |
| *FCGR3A* | ENSBTAG00000002096 | Fc fragment of IgG, low affinity IIIa, receptor (CD16a) | [203] |
| *FCHO2* | ENSBTAG00000017545 | FCH and mu domain containing endocytic adaptor 2 | [203] |
| *FCHSD2* | ENSBTAG00000019062 | FCH and double SH3 domains 2 | [139] |
| *FCNB* | ENSBTAG00000048155 | Ficolin 1 | [51] |
| *FCSK* | ENSBTAG00000012985 | Fucose kinase | [205] |
| *FDX2* | ENSBTAG00000010318 | Ferredoxin 2 | [203] |
| *FER1L6* | ENSBTAG00000013537 | Fer-1 like family member 6 | [203] |
| *FERMT3* | ENSBTAG00000045862 | Fermitin family member 3 | [51] |
| *FETUB* | ENSBTAG00000017531 | Fetuin B | [203] |
| *FEV* | ENSBTAG00000020191 | FEV transcription factor, ETS family member | [203] |
| *FEZ1* | ENSBTAG00000009124 | Fasciculation and elongation protein zeta 1 | [138] |
| *FEZF1* | ENSBTAG00000005103 | FEZ family zinc finger 1 | [203] |
| *FGB* | ENSBTAG00000022120 | Fibrinogen beta chain | [203] |
| *FGF1* | ENSBTAG00000005198 | Fibroblast growth factor 1 | [52] |
| *FGF12* | ENSBTAG00000012413 | Fibroblast growth factor 12 | [203] |
| *FGF22* | ENSBTAG00000027357 | Fibroblast growth factor 22 | [203] |
| *FGFBP3* | ENSBTAG00000039324 | Fibroblast growth factor binding protein 3 | [203] |
| *FGFRL1* | ENSBTAG00000027464 | Fibroblast growth factor receptor like 1 | [203] |
| *FGG* | ENSBTAG00000006745 | Fibrinogen gamma chain | [139] |
| *FGL1* | ENSBTAG00000016177 | Fibrinogen like 1 | [139] |
| *FGL2* | ENSBTAG00000009717 | Fibrinogen like 2 | [139] |
| *FGR* | ENSBTAG00000011784 | FGR proto-oncogene, Src family tyrosine kinase | [52] |
| *FHIT* | ENSBTAG00000014418 | Fragile histidine triad diadenosine triphosphatase | [203] |
| *FHL5* | ENSBTAG00000001803 | Four and a half LIM domains 5 | [203] |
| *FIBIN* | ENSBTAG00000006676 | Fin bud initiation factor homolog | [203] |
| *FKBP5* | ENSBTAG00000047502 | FKBP prolyl isomerase 5 | [138] |
| *FLI1* | ENSBTAG00000008283 | Fli-1 proto-oncogene, ETS transcription factor | [139] |
| *FLNA* | ENSBTAG00000011190 | Filamin A | [204] |
| *FLVCR1* | ENSBTAG00000015974 | FLVCR heme transporter 1 | [140] |
| *FLVCR2* | ENSBTAG00000040078 | Feline leukemia virus subgroup C cellular receptor family, member 2 | [203] |
| *FMNL1* | ENSBTAG00000004196 | Formin like 1 | [203] |
| *FMNL3* | ENSBTAG00000016593 | Formin like 3 | [139] |
| *FMO1* | ENSBTAG00000021408 | Flavin containing dimethylaniline monoxygenase 1 | [203] |
| *FMO4* | ENSBTAG00000016685 | Flavin containing dimethylaniline monoxygenase 4 | [139] |
| *FMOD* | ENSBTAG00000014912 | Fibromodulin | [139] |
| *FN1* | ENSBTAG00000008300 | Fibronectin 1 | [52] |
| *FNDC11* | ENSBTAG00000027405 | Fibronectin type III domain containing 11 | [203] |
| *FOSL2* | ENSBTAG00000023929 | FOS like 2, AP-1 transcription factor subunit | [205] |
| *FOXA1* | ENSBTAG00000013888 | Forkhead box A1 | [139] |
| *FOXA3* | ENSBTAG00000012104 | Forkhead box A3 | [139] |
| *FOXE3* | ENSBTAG00000051575 | Forkhead box E3 | [203] |
| *FOXF1* | ENSBTAG00000000009 | Forkhead box F1 | [139] |
| *FOXI2* | ENSBTAG00000006218 | Forkhead box I2 | [203] |
| *FOXO3* | ENSBTAG00000011234 | Forkhead box O3 | [203] |
| *FOXO4* | ENSBTAG00000004879 | Forkhead box O4 | [203] |
| *FOXQ1* | ENSBTAG00000052132 | Forkhead box Q1 | [139] |
| *FPGT* | ENSBTAG00000038480 | Fucose-1-phosphate guanylyltransferase | [203] |
| *FRK* | ENSBTAG00000020018 | Fyn related Src family tyrosine kinase | [140] |
| *FRMPD4* | ENSBTAG00000017785 | FERM and PDZ domain containing 4 | [203] |
| *FRY* | ENSBTAG00000006771 | FRY microtubule binding protein | [203] |
| *FSD1L* | ENSBTAG00000013420 | Fibronectin type III and SPRY domain containing 1 like | [139] |
| *FSTL4* | ENSBTAG00000017105 | Follistatin like 4 | [203] |
| *FTH1* | ENSBTAG00000011184 | Ferritin heavy chain 1 | [138] |
| *FUN14* | ENSBTAG00000003118 | FUN14 domain-containing protein 1-like | [140] |
| *FUS* | ENSBTAG00000005757 | FUS RNA binding protein | [140] |
| *FUT5* | ENSBTAG00000000414 | Fucosyltransferase 6 | [203] |
| *FZD10* | ENSBTAG00000007047 | Frizzled class receptor 10 | [138] |
| *FZD5* | ENSBTAG00000020957 | Frizzled class receptor 5 | [138] |
| *FZR1* | ENSBTAG00000031387 | Fizzy and cell division cycle 20 related 1 | [140] |
| *GABARAP* | ENSBTAG00000014883 | GABA type A receptor-associated protein | [138] |
| *GABRR1* | ENSBTAG00000011672 | Gamma-aminobutyric acid type A receptor subunit rho1 | [203] |
| *GADD45G* | ENSBTAG00000003033 | Growth arrest and DNA damage inducible gamma | [204] |
| *GADD45GIP1* | ENSBTAG00000015117 | GADD45G interacting protein 1 | [203] |
| *GAL* | ENSBTAG00000009393 | Galanin and GMAP prepropeptide | [203] |
| *GALM* | ENSBTAG00000021102 | Galactose mutarotase | [138] |
| *GALNT11* | ENSBTAG00000021260 | Polypeptide N-acetylgalactosaminyltransferase 11 | [138] |
| *GALNT18* | ENSBTAG00000002914 | Polypeptide N-acetylgalactosaminyltransferase 18 | [203] |
| *GAN* | ENSBTAG00000009428 | Gigaxonin | [203] |
| *GAP43* | ENSBTAG00000006451 | Growth associated protein 43 | [203] |
| *GAPDH* | ENSBTAG00000014731 | Glyceraldehyde-3-phosphate dehydrogenase | [51] |
| *GAS7* | ENSBTAG00000019107 | Growth arrest specific 7 | [203] |
| *GAS8* | ENSBTAG00000007096 | Growth arrest specific 8 | [203] |
| *GATA2* | ENSBTAG00000019707 | GATA binding protein 2 | [203] |
| *GATD1* | ENSBTAG00000010348 | Glutamine amidotransferase like class 1 domain containing 1 | [203] |
| *GATM* | ENSBTAG00000005586 | Glycine amidinotransferase | [139] |
| *GBF1* | ENSBTAG00000006014 | Bos taurus golgi brefeldin A resistant guanine nucleotide exchange factor 1 (GBF1), mRNA | [203] |
| *GBP4* | ENSBTAG00000014529 | Guanylate binding protein 4 | [139] |
| *GBP5* | ENSBTAG00000015060 | Guanylate binding protein 5 | [139] |
| *GBP6* | ENSBTAG00000031186 | Guanylate binding protein family, member 6 | [139] |
| *GCA* | ENSBTAG00000018446 | Grancalcin | [139] |
| *GCC1* | ENSBTAG00000003102 | GRIP and coiled-coil domain containing 1 | [203] |
| *GCH1* | ENSBTAG00000040151 | GTP cyclohydrolase 1 | [140] |
| *GCM2* | ENSBTAG00000018787 | Glial cells missing transcription factor 2 | [203] |
| *GCNT2* | ENSBTAG00000039326 | N-acetyllactosaminide beta-1,6-N-acetylglucosaminyl-transferase | [203] |
| *GDAP2* | ENSBTAG00000016387 | Ganglioside induced differentiation associated protein 2 | [140] |
| *GDE1* | ENSBTAG00000002101 | Glycerophosphodiester phosphodiesterase 1 | [140] |
| *GDF10* | ENSBTAG00000001019 | Growth differentiation factor 10 | [203] |
| *GDF9* | ENSBTAG00000009478 | Growth differentiation factor 9 | [203] |
| *GDPD1* | ENSBTAG00000003365 | Glycerophosphodiester phosphodiesterase domain containing 1 | [139] |
| *GDPD4* | ENSBTAG00000000886 | Glycerophosphodiester phosphodiesterase domain containing 4 | [203] |
| *GEM* | ENSBTAG00000007596 | GTP binding protein overexpressed in skeletal muscle | [139] |
| *GFAP* | ENSBTAG00000013534 | Glial fibrillary acidic protein | [203] |
| *GFER* | ENSBTAG00000017631 | Growth factor, augmenter of liver regeneration | [140] |
| *GFM2* | ENSBTAG00000015519 | GTP dependent ribosome recycling factor mitochondrial 2 | [140] |
| *GFPT1* | ENSBTAG00000017626 | Glutamine--fructose-6-phosphate transaminase 1 | [204] |
| *GFRA1* | ENSBTAG00000019297 | GDNF family receptor alpha 1 | [203] |
| *GGA3* | ENSBTAG00000016128 | Golgi associated, gamma adaptin ear containing, ARF binding protein 3 | [203] |
| *GGT5* | ENSBTAG00000006608 | Gamma-glutamyltransferase 5 | [203] |
| *GHITM* | ENSBTAG00000032829 | Growth hormone inducible transmembrane protein | [140] |
| *GHRL* | ENSBTAG00000012328 | Ghrelin and obestatin prepropeptide | [203] |
| *GIGYF1* | ENSBTAG00000015738 | GRB10 interacting GYF protein 1 | [203] |
| *GIMAP5* | ENSBTAG00000004894 | GTPase, IMAP family member 5 | [138] |
| *GIN1* | ENSBTAG00000021471 | Gypsy retrotransposon integrase 1 | [203] |
| *GIT2* | ENSBTAG00000006506 | GIT ArfGAP 2 | [203] |
| *GJA10* | ENSBTAG00000011315 | Gap junction protein alpha 10 | [203] |
| *GJA5* | ENSBTAG00000006720 | Gap junction protein alpha 5 | [203] |
| *GJB6* | ENSBTAG00000038662 | Gap junction protein beta 6 | [139] |
| *GJD4* | ENSBTAG00000003640 | Gap junction protein delta 4 | [203] |
| *GLG1* | ENSBTAG00000002303 | Golgi glycoprotein 1 | [205] |
| *GLI2* | ENSBTAG00000011682 | GLI family zinc finger 2 | [203] |
| *GLIS1* | ENSBTAG00000017986 | GLIS family zinc finger 1 | [203] |
| *GLMN* | ENSBTAG00000018737 | Glomulin, FKBP associated protein | [139] |
| *GLRB* | ENSBTAG00000021764 | Glycine receptor beta | [203] |
| *GLRX2* | ENSBTAG00000015972 | Glutaredoxin 2 | [140] |
| *GLT8D2* | ENSBTAG00000000925 | Glycosyltransferase 8 domain containing 2 | [51] |
| *GLUL* | ENSBTAG00000013631 | Glutamate-ammonia ligase | [138] |
| *GLYR1* | ENSBTAG00000001731 | Glyoxylate reductase 1 homolog | [203] |
| *GMDS* | ENSBTAG00000012058 | GDP-mannose 4,6-dehydratase | [138] |
| *GMIP* | ENSBTAG00000015144 | GEM interacting protein | [51] |
| *GMNN* | ENSBTAG00000017329 | Geminin DNA replication inhibitor | [203] |
| *GNAL* | ENSBTAG00000002125 | G protein subunit alpha L | [138] |
| *GNG11* | ENSBTAG00000000820 | G protein subunit gamma 11 | [139] |
| *GNG2* | ENSBTAG00000003043 | G protein subunit gamma 2 | [204] |
| *GNGT1* | ENSBTAG00000002674 | G protein subunit gamma transducin 1 | [203] |
| *GNRH1* | ENSBTAG00000000164 | Gonadotropin releasing hormone 1 | [203] |
| *GOLGA2* | ENSBTAG00000011317 | Golgin A2 | [140] |
| *GOLGA4* | ENSBTAG00000016563 | Golgin A4 | [139] |
| *GORAB* | ENSBTAG00000003295 | Golgin, RAB6 interacting | [139] |
| *GOT2* | ENSBTAG00000007172 | Glutamic-oxaloacetic transaminase 2 | [204] |
| *GP1BA* | ENSBTAG00000004909 | Glycoprotein Ib platelet subunit alpha | [203] |
| *GPAM* | ENSBTAG00000011917 | Glycerol-3-phosphate acyltransferase, mitochondrial | [51] |
| *GPATCH2L* | ENSBTAG00000003084 | G-patch domain containing 2 like | [203] |
| *GPATCH3* | ENSBTAG00000014849 | G-patch domain containing 3 | [203] |
| *GPBP1* | ENSBTAG00000012124 | GC-rich promoter binding protein 1 | [138] |
| *GPD1L* | ENSBTAG00000009826 | Glycerol-3-phosphate dehydrogenase 1 like | [139] |
| *GPM6A* | ENSBTAG00000004231 | Glycoprotein M6A | [203] |
| *GPNMB* | ENSBTAG00000000604 | Glycoprotein nmb | [139] |
| *GPR155* | ENSBTAG00000021636 | G protein-coupled receptor 155 | [203] |
| *GPR156* | ENSBTAG00000001464 | G protein-coupled receptor 156 | [205] |
| *GPR161* | ENSBTAG00000000616 | G protein-coupled receptor 161 | [203] |
| *GPR182* | ENSBTAG00000039157 | G protein-coupled receptor 182 | [203] |
| *GPRASP1* | ENSBTAG00000047213 | G protein-coupled receptor associated sorting protein 1 | [203] |
| *GPSM3* | ENSBTAG00000054438 | G protein signaling modulator 3 | [51] |
| *GPX1* | ENSBTAG00000054195 | Glutathione peroxidase 1 | [51] |
| *GPX3* | ENSBTAG00000043553 | Glutathione peroxidase 3 | [52] |
| *GRAP* | ENSBTAG00000003707 | GRB2-related adaptor protein | [203] |
| *GRAP2* | ENSBTAG00000015157 | GRB2 related adaptor protein 2 | [203] |
| *GRB14* | ENSBTAG00000019291 | Growth factor receptor bound protein 14 | [203] |
| *GREB1* | ENSBTAG00000015402 | Growth regulating estrogen receptor binding 1 | [203] |
| *GRINA* | ENSBTAG00000000312 | Glutamate ionotropic receptor NMDA type subunit associated protein 1 | [139] |
| *GRK5* | ENSBTAG00000007981 | G protein-coupled receptor kinase 5 | [203] |
| *GRM1* | ENSBTAG00000044181 | Glutamate metabotropic receptor 1 | [203] |
| *GRM7* | ENSBTAG00000013047 | Glutamate metabotropic receptor 7 | [203] |
| *GRO1* | ENSBTAG00000037558 | Chemokine (C-X-C motif) ligand 1 (melanoma growth stimulating activity, alpha) | [203] |
| *GSAP* | ENSBTAG00000018430 | Gamma-secretase activating protein | [203] |
| *GSDMC* | ENSBTAG00000017478 | Gasdermin C | [203] |
| *GSG1L* | ENSBTAG00000004607 | GSG1 like | [203] |
| *GSK3B* | ENSBTAG00000048057 | Glycogen synthase kinase 3 beta | [204] |
| *GSPT1* | ENSBTAG00000003577 | G1 to S phase transition | [140] |
| *GSS* | ENSBTAG00000003504 | Glutathione synthetase | [203] |
| *GSTM1* | ENSBTAG00000017765 | Bos taurus glutathione S-transferase M1 (GSTM1), mRNA | [204] |
| *GSTZ1* | ENSBTAG00000002706 | Glutathione S-transferase zeta 1 | [203] |
| *GTF2H2* | ENSBTAG00000027983 | General transcription factor IIH, polypeptide 2, 44kDa | [138] |
| *GTF3C4* | ENSBTAG00000004091 | General transcription factor IIIC subunit 4 | [140] |
| *GTPBP10* | ENSBTAG00000032166 | GTP binding protein 10 | [203] |
| *GUCD1* | ENSBTAG00000022022 | Guanylyl cyclase domain containing 1 | [203] |
| *GUCY1B3* | ENSBTAG00000003840 | Guanylate cyclase 1 soluble subunit beta 1 | [139] |
| *GYPC* | ENSBTAG00000014863 | Glycophorin C | [203] |
| *H2AC6* | ENSBTAG00000039657 | H2A clustered histone 6 | [203] |
| *H2BC5* | ENSBTAG00000031778 | H2B clustered histone 5 | [203] |
| *HACE1* | ENSBTAG00000001533 | HECT domain and ankyrin repeat containing E3 ubiquitin protein ligase 1 | [203] |
| *HAND1* | ENSBTAG00000002335 | Heart and neural crest derivatives expressed 1 | [203] |
| *HAPLN1* | ENSBTAG00000012411 | Hyaluronan and proteoglycan link protein 1 | [52] |
| *HAS2* | ENSBTAG00000019892 | Hyaluronan synthase 2 | [203] |
| *HBB* | ENSBTAG00000037644 | Hemoglobin, beta | [203] |
| *HCK* | ENSBTAG00000007932 | HCK proto-oncogene, Src family tyrosine kinase | [51] |
| *HCST* | ENSBTAG00000007336 | Hematopoietic cell signal transducer | [203] |
| *HDAC1* | ENSBTAG00000012698 | Histone deacetylase 1 | [140] |
| *HDHD5* | ENSBTAG00000016995 | Haloacid dehalogenase like hydrolase domain containing 5 | [203] |
| *HEATR4* | ENSBTAG00000002842 | HEAT repeat containing 4 | [203] |
| *HEPACAM* | ENSBTAG00000018650 | Hepatic and glial cell adhesion molecule | [203] |
| *HEPH* | ENSBTAG00000047416 | Hephaestin | [203] |
| *HERC3* | ENSBTAG00000010120 | HECT and RLD domain containing E3 ubiquitin protein ligase 3 | [203] |
| *HERC5* | ENSBTAG00000020538 | HECT and RLD domain containing E3 ubiquitin protein ligase 5 | [140] |
| *HES5* | ENSBTAG00000002483 | Hes family bHLH transcription factor 5 | [203] |
| *HGS* | ENSBTAG00000000411 | Hepatocyte growth factor-regulated tyrosine kinase substrate | [203] |
| *HHIP* | ENSBTAG00000016071 | Hedgehog interacting protein | [203] |
| *HIF1A* | ENSBTAG00000020935 | Hypoxia inducible factor 1 subunit alpha | [204] |
| *HIGD1B* | ENSBTAG00000016736 | HIG1 hypoxia inducible domain family member 1B | [138] |
| *HJURP* | ENSBTAG00000024726 | Holliday junction recognition protein | [139] |
| *HK2* | ENSBTAG00000013108 | Hexokinase 2 | [139] |
| *HK3* | ENSBTAG00000014898 | Hexokinase 3 | [51] |
| *HLF* | ENSBTAG00000006618 | HLF transcription factor, PAR bZIP family member | [139] |
| *HMGB3* | ENSBTAG00000005150 | High mobility group box 3 | [139] |
| *HMGCL* | ENSBTAG00000021832 | 3-hydroxy-3-methylglutaryl-CoA lyase | [204] |
| *HMGCS1* | ENSBTAG00000011839 | 3-hydroxy-3-methylglutaryl-CoA synthase 1 | [140] |
| *HMGCS2* | ENSBTAG00000003898 | 3-hydroxy-3-methylglutaryl-CoA synthase 2 | [140] |
| *HMGN4* | ENSBTAG00000031747 | High mobility group nucleosomal binding domain 4 | [138] |
| *HMOX1* | ENSBTAG00000015582 | Heme oxygenase 1 | [51] |
| *HMOX2* | ENSBTAG00000018937 | Heme oxygenase 2 | [139] |
| *HNRNPDL* | ENSBTAG00000014804 | Heterogeneous nuclear ribonucleoprotein D like | [204] |
| *HNRNPF* | ENSBTAG00000008853 | Heterogeneous nuclear ribonucleoprotein F | [138] |
| *HNRNPL* | ENSBTAG00000001055 | Heterogeneous nuclear ribonucleoprotein L | [140] |
| *HOOK1* | ENSBTAG00000030671 | Hook microtubule tethering protein 1 | [203] |
| *HOXA2* | ENSBTAG00000008138 | Homeobox A2 | [203] |
| *HOXA4* | ENSBTAG00000001063 | Homeobox A4 | [139] |
| *HOXB4* | ENSBTAG00000039599 | Homeobox B4 | [203] |
| *HP* | ENSBTAG00000006354 | Haptoglobin | [138] |
| *HPS3* | ENSBTAG00000002395 | HPS3 biogenesis of lysosomal organelles complex 2 subunit 1 | [203] |
| *HS2ST1* | ENSBTAG00000017595 | Heparan sulfate 2-O-sulfotransferase 1 | [138] |
| *HSD11B1* | ENSBTAG00000015086 | Hydroxysteroid 11-beta dehydrogenase 1 | [139] |
| *HSD11B2* | ENSBTAG00000005685 | Hydroxysteroid 11-beta dehydrogenase 2 | [203] |
| *HSD17B13* | ENSBTAG00000031679 | Hydroxysteroid 17-beta dehydrogenase 13 | [203] |
| *HSD3B1* | ENSBTAG00000006769 | Hydroxy-delta-5-steroid dehydrogenase, 3 beta- and steroid delta-isomerase 1 | [139] |
| *HSH2D* | ENSBTAG00000038154 | Hematopoietic SH2 domain containing | [51] |
| *HSP90AB1* | ENSBTAG00000000778 | Heat shock protein 90 alpha family class B member 1 | [138] |
| *HSPA13* | ENSBTAG00000012908 | Heat shock protein family A (Hsp70) member 13 | [139] |
| *HSPA8* | ENSBTAG00000013162 | Heat shock protein family A (Hsp70) member 8 | [138] |
| *HSPB3* | ENSBTAG00000022714 | Heat shock protein family B (small) member 3 | [203] |
| *HSPB8* | ENSBTAG00000001303 | Heat shock protein family B (small) member 8 | [139] |
| *HSPD1* | ENSBTAG00000012586 | Heat shock protein family D (Hsp60) member 1 | [204] |
| *HTR1B* | ENSBTAG00000037429 | 5-hydroxytryptamine receptor 1B | [203] |
| *HTR1E* | ENSBTAG00000026580 | 5-hydroxytryptamine receptor 1E | [203] |
| *HTR2A* | ENSBTAG00000013498 | 5-hydroxytryptamine receptor 2A | [203] |
| *IAH1* | ENSBTAG00000001140 | Isoamyl acetate hydrolyzing esterase 1 (putative) | [138] |
| *ICAM1* | ENSBTAG00000010303 | Intercellular adhesion molecule 1 | [138] |
| *ICAM3* | ENSBTAG00000015742 | Intercellular adhesion molecule 3 | [51] |
| *ICOSLG* | ENSBTAG00000014880 | Inducible T cell costimulator ligand | [139] |
| *ID3* | ENSBTAG00000030425 | Inhibitor of DNA binding 3, HLH protein | [139] |
| *IDH3A* | ENSBTAG00000006227 | Isocitrate dehydrogenase (NAD(+)) 3 catalytic subunit alpha | [140] |
| *IDH3B* | ENSBTAG00000018813 | Isocitrate dehydrogenase (NAD(+)) 3 non-catalytic subunit beta | [138] |
| *IDO1* | ENSBTAG00000020602 | Indoleamine 2,3-dioxygenase 1 | [140] |
| *IDS* | ENSBTAG00000011056 | Iduronate 2-sulfatase | [204] |
| *IER3* | ENSBTAG00000011358 | Immediate early response 3 | [138] |
| *IER5* | ENSBTAG00000030521 | Immediate early response 5 like | [51] |
| *IFI16* | ENSBTAG00000011511 | Interferon gamma inducible protein 16 | [139] |
| *IFI27L2* | ENSBTAG00000003155 | Interferon, alpha-inducible protein 27-like 2 | [139] |
| *IFI35* | ENSBTAG00000007389 | Interferon induced protein 35 | [205] |
| *IFI44* | ENSBTAG00000034349 | Interferon induced protein 44 | [139] |
| *IFI44L* | ENSBTAG00000030932 | Interferon induced protein 44 like | [139] |
| *IFI6* | ENSBTAG00000007554 | Interferon alpha inducible protein 6 | [138] |
| *IFIT3* | ENSBTAG00000009768 | Interferon induced protein with tetratricopeptide repeats 3 | [140] |
| *IFIT5* | ENSBTAG00000017367 | Interferon induced protein with tetratricopeptide repeats 5 | [139] |
| *IFITM1* | ENSBTAG00000048470 | Interferon induced transmembrane protein 1 (9-27) | [51] |
| *IFITM3* | ENSBTAG00000019015 | Interferon induced transmembrane protein 3 (1-8U) | [51] |
| *IFNAR2* | ENSBTAG00000015212 | Interferon alpha and beta receptor subunit 2 | [51] |
| *IFNB* | ENSBTAG00000055103 | Bos taurus interferon, beta 3 (IFNB3), mRNA | [140] |
| *IFNB2* | ENSBTAG00000054945 | Interleukin 6 (interferon, beta 2) | [203] |
| *IFRD1* | ENSBTAG00000010549 | Interferon related developmental regulator 1 | [139] |
| *IFT43* | ENSBTAG00000012005 | Intraflagellar transport 43 | [203] |
| *IGF2* | ENSBTAG00000013066 | Bos taurus insulin like growth factor 2 (IGF2), transcript variant 2, mRNA | [203] |
| *IGF2BP3* | ENSBTAG00000019406 | Insulin like growth factor 2 mRNA binding protein 3 | [203] |
| *IGFBP1* | ENSBTAG00000046768 | Insulin like growth factor binding protein 1 | [140] |
| *IGFBP3* | ENSBTAG00000003994 | Insulin like growth factor binding protein 3 | [139] |
| *IGFBP4* | ENSBTAG00000008611 | Insulin like growth factor binding protein 4 | [52] |
| *IGLL1* | ENSBTAG00000055118 | Pre-B lymphocyte 1 | [204] |
| *IGSF6* | ENSBTAG00000018869 | Immunoglobulin superfamily member 6 | [203] |
| *IKBKE* | ENSBTAG00000010408 | Inhibitor of nuclear factor kappa B kinase subunit epsilon | [139] |
| *IL10RB* | ENSBTAG00000019404 | Interleukin 10 receptor subunit beta | [138] |
| *IL11* | ENSBTAG00000014907 | Interleukin 11 receptor subunit alpha | [203] |
| *IL17RD* | ENSBTAG00000010999 | Interleukin 17 receptor D | [203] |
| *IL18* | ENSBTAG00000000277 | Interleukin 18 | [52] |
| *IL18BP* | ENSBTAG00000027676 | Interleukin 18 binding protein | [51] |
| *IL18RAP* | ENSBTAG00000033748 | Interleukin 18 receptor accessory protein | [203] |
| *IL1A* | ENSBTAG00000010349 | Interleukin 1 alpha | [139] |
| *IL1B* | ENSBTAG00000001321 | Interleukin 1 beta | [51] |
| *IL1RAP* | ENSBTAG00000013205 | Interleukin 1 receptor accessory protein | [140] |
| *IL2* | ENSBTAG00000020883 | Interleukin 2 | [140] |
| *IL20RA* | ENSBTAG00000015638 | Interleukin 20 receptor subunit alpha | [139] |
| *IL20RB* | ENSBTAG00000008299 | Interleukin 20 receptor subunit beta | [203] |
| *IL21* | ENSBTAG00000012368 | Interleukin 21 | [203] |
| *IL23A* | ENSBTAG00000004378 | Interleukin 23 subunit alpha | [140] |
| *IL2RG* | ENSBTAG00000007626 | Interleukin 2 receptor subunit gamma | [51] |
| *IL34* | ENSBTAG00000009357 | Interleukin 34 | [205] |
| *IL36A* | ENSBTAG00000002087 | Interleukin 36 alpha | [203] |
| *IL4R* | ENSBTAG00000001602 | Interleukin 4 receptor | [139] |
| *IL6* | ENSBTAG00000014921 | Interleukin 6 | [139] |
| *IMPDH2* | ENSBTAG00000031837 | Inosine monophosphate dehydrogenase 2 | [138] |
| *ING2* | ENSBTAG00000047706 | Inhibitor of growth family member 2 | [203] |
| *INHBB* | ENSBTAG00000049058 | Inhibin subunit beta B | [203] |
| *INO80E* | ENSBTAG00000005030 | INO80 complex subunit E | [138] |
| *INPP4B* | ENSBTAG00000014111 | Inositol polyphosphate-4-phosphatase type II B | [203] |
| *INSYN1* | ENSBTAG00000025803 | Inhibitory synaptic factor 1 | [203] |
| *INTS13* | ENSBTAG00000013515 | Integrator complex subunit 13 | [203] |
| *INTS6* | ENSBTAG00000002970 | Integrator complex subunit 6 | [204] |
| *IPCEF1* | ENSBTAG00000003489 | Interaction protein for cytohesin exchange factors 1 | [139] |
| *IPMK* | ENSBTAG00000011197 | Inositol polyphosphate multikinase | [139] |
| *IPO13* | ENSBTAG00000018887 | Importin 13 | [203] |
| *IPO4* | ENSBTAG00000002707 | Importin 4 | [203] |
| *IQCA1* | ENSBTAG00000013505 | IQ motif containing with AAA domain 1 | [203] |
| *IQCB1* | ENSBTAG00000013069 | IQ motif containing B1 | [139] |
| *IRF1* | ENSBTAG00000031231 | Interferon regulatory factor 1 | [139] |
| *IRF2* | ENSBTAG00000010002 | Interferon regulatory factor 2 | [140] |
| *IRF3* | ENSBTAG00000006633 | Interferon regulatory factor 3 | [203] |
| *IRF7* | ENSBTAG00000047680 | Interferon regulatory factor 7 | [140] |
| *IRF9* | ENSBTAG00000005816 | Interferon regulatory factor 9 | [51] |
| *IRGC* | ENSBTAG00000013766 | Immunity related GTPase cinema | [203] |
| *IRX6* | ENSBTAG00000016407 | Iroquois homeobox 6 | [203] |
| *ISCA2* | ENSBTAG00000021956 | Iron-sulfur cluster assembly 2 | [203] |
| *ISG15* | ENSBTAG00000014707 | ISG15 ubiquitin like modifier | [138] |
| *ISG20* | ENSBTAG00000014762 | Interferon stimulated exonuclease gene 20 | [139] |
| *ISG20L2* | ENSBTAG00000001518 | Interferon stimulated exonuclease gene 20 like 2 | [204] |
| *ISOC2* | ENSBTAG00000015950 | Isochorismatase domain containing 2 | [140] |
| *ITCH* | ENSBTAG00000000308 | Itchy E3 ubiquitin protein ligase | [203] |
| *ITGA2* | ENSBTAG00000019289 | Integrin subunit alpha 2 | [205] |
| *ITGA5* | ENSBTAG00000013745 | Integrin subunit alpha 5 | [139] |
| *ITGA8* | ENSBTAG00000007602 | Integrin subunit alpha 8 | [139] |
| *ITGA9* | ENSBTAG00000016566 | Integrin subunit alpha 9 | [203] |
| *ITGAL* | ENSBTAG00000007103 | Integrin subunit alpha L | [51] |
| *ITPKC* | ENSBTAG00000019465 | Inositol-trisphosphate 3-kinase C | [139] |
| *ITPR2* | ENSBTAG00000002313 | Inositol 1,4,5-trisphosphate receptor type 2 | [203] |
| *ITSN2* | ENSBTAG00000023172 | Intersectin 2 | [205] |
| *JAK1* | ENSBTAG00000003147 | Janus kinase 1 | [204] |
| *JAKMIP1* | ENSBTAG00000011170 | Janus kinase and microtubule interacting protein 1 | [205] |
| *JDP2* | ENSBTAG00000009451 | Jun dimerization protein 2 | [203] |
| *JSP1* | ENSBTAG00000020116 | Bos taurus uncharacterized protein 100125016 (LOC100125916), mRNA | [203] |
| *JUN* | ENSBTAG00000004037 | Jun proto-oncogene, AP-1 transcription factor subunit | [140] |
| *JUNB* | ENSBTAG00000012046 | JunB proto-oncogene, AP-1 transcription factor subunit | [51] |
| *KANK1* | ENSBTAG00000010976 | KN motif and ankyrin repeat domains 1 | [203] |
| *KAT6B* | ENSBTAG00000002920 | Lysine acetyltransferase 6B | [203] |
| *KCNA2* | ENSBTAG00000015459 | Potassium voltage-gated channel subfamily A member 2 | [203] |
| *KCNE1* | ENSBTAG00000001150 | Potassium voltage-gated channel subfamily E regulatory subunit 1 | [139] |
| *KCNG2* | ENSBTAG00000013665 | Potassium voltage-gated channel modifier subfamily G member 2 | [203] |
| *KCNJ10* | ENSBTAG00000002414 | Potassium inwardly rectifying channel subfamily J member 10 | [203] |
| *KCNJ15* | ENSBTAG00000000973 | Potassium inwardly rectifying channel subfamily J member 15 | [203] |
| *KCNK1* | ENSBTAG00000004515 | Potassium two pore domain channel subfamily K member 1 | [204] |
| *KCNK17* | ENSBTAG00000010328 | Potassium two pore domain channel subfamily K member 17 | [138] |
| *KCNK5* | ENSBTAG00000011112 | Potassium two pore domain channel subfamily K member 5 | [203] |
| *KCNMB1* | ENSBTAG00000002985 | Potassium calcium-activated channel subfamily M regulatory beta subunit 1 | [203] |
| *KCNMB2* | ENSBTAG00000011377 | Potassium calcium-activated channel subfamily M regulatory beta subunit 2 | [203] |
| *KCNMB4* | ENSBTAG00000003749 | Potassium calcium-activated channel subfamily M regulatory beta subunit 4 | [203] |
| *KCNRG* | ENSBTAG00000008176 | Potassium channel regulator | [203] |
| *KCNV1* | ENSBTAG00000008512 | Potassium voltage-gated channel modifier subfamily V member 1 | [203] |
| *KCTD11* | ENSBTAG00000010532 | Potassium channel tetramerization domain containing 11 | [139] |
| *KCTD20* | ENSBTAG00000020595 | Potassium channel tetramerization domain containing 20 | [203] |
| *KCTD8* | ENSBTAG00000038659 | Potassium channel tetramerization domain containing 8 | [203] |
| *KDM5C* | ENSBTAG00000014943 | Lysine demethylase 5C | [138] |
| *KDM7A* | ENSBTAG00000003495 | Lysine demethylase 7A | [203] |
| *KEL* | ENSBTAG00000014533 | Kell metallo-endopeptidase | [203] |
| *KERA* | ENSBTAG00000014340 | Keratocan | [52] |
| *KIAA0232* | ENSBTAG00000019427 | KIAA0232 | [203] |
| *KIAA0408* | ENSBTAG00000003919 | KIAA0408 | [138] |
| *KIAA0586* | ENSBTAG00000004631 | KIAA0586 | [204] |
| *KIAA0753* | ENSBTAG00000015593 | KIAA0753 | [203] |
| *KIAA0895L* | ENSBTAG00000003521 | KIAA0895 like | [203] |
| *KIAA1109* | ENSBTAG00000048798 | KIAA1109 ortholog | [139] |
| *KIAA2013* | ENSBTAG00000019388 | KIAA2013 ortholog | [203] |
| *KIF12* | ENSBTAG00000015685 | Kinesin family member 12 | [203] |
| *KIF13A* | ENSBTAG00000019217 | Kinesin family member 13A | [203] |
| *KIF17* | ENSBTAG00000017644 | Kinesin family member 17 | [203] |
| *KIF18A* | ENSBTAG00000002117 | Kinesin family member 18A | [203] |
| *KIF20A* | ENSBTAG00000008758 | Kinesin family member 20A | [139] |
| *KIF21A* | ENSBTAG00000004832 | Kinesin family member 21A | [203] |
| *KIF22* | ENSBTAG00000013669 | Kinesin family member 22 | [139] |
| *KIF23* | ENSBTAG00000009983 | Kinesin family member 23 | [139] |
| *KIF2A* | ENSBTAG00000015022 | Kinesin family member 2A | [203] |
| *KIF2C* | ENSBTAG00000015280 | Kinesin family member 2C | [139] |
| *KIRREL3* | ENSBTAG00000006804 | Kirre like nephrin family adhesion molecule 3 | [203] |
| *KIT* | ENSBTAG00000002699 | KIT proto-oncogene, receptor tyrosine kinase | [52] |
| *KLC3* | ENSBTAG00000002070 | Kinesin light chain 3 | [203] |
| *KLF4* | ENSBTAG00000020355 | Kruppel like factor 4 | [139] |
| *KLHDC9* | ENSBTAG00000020150 | Kelch domain containing 9 | [203] |
| *KLHL12* | ENSBTAG00000009726 | Kelch like family member 12 | [203] |
| *KLHL38* | ENSBTAG00000016754 | Kelch like family member 38 | [203] |
| *KLHL8* | ENSBTAG00000002455 | Kelch like family member 8 | [51] |
| *KLK10* | ENSBTAG00000015129 | Kallikrein related peptidase 10 | [51] |
| *KMT5A* | ENSBTAG00000000139 | Lysine methyltransferase 5A | [203] |
| *KNTC1* | ENSBTAG00000019278 | Kinetochore associated 1 | [139] |
| *KPNA3* | ENSBTAG00000004850 | Karyopherin subunit alpha 3 | [203] |
| *KRAS* | ENSBTAG00000009778 | KRAS proto-onco, GTPase | [203] |
| *KREMEN1* | ENSBTAG00000009773 | Kringle containing transmembrane protein 1 | [203] |
| *KRT20* | ENSBTAG00000007794 | Keratin 20 | [203] |
| *KRT4* | ENSBTAG00000012034 | Keratin 4 | [203] |
| *KRT82* | ENSBTAG00000007145 | Keratin 82 | [203] |
| *KTN1* | ENSBTAG00000017713 | Kinectin 1 | [139] |
| *LA-DQB* | ENSBTAG00000019588 | MHC class II antigen | [203] |
| *LAMA1* | ENSBTAG00000018160 | Laminin subunit alpha 1 | [138] |
| *LAMC2* | ENSBTAG00000000793 | Laminin subunit gamma 2 | [204] |
| *LAMP3* | ENSBTAG00000006552 | Lysosomal associated membrane protein 3 | [203] |
| *LAP* | ENSBTAG00000053889 | Lingual antimicrobial peptide | [51] |
| *LAP3* | ENSBTAG00000005989 | Leucine aminopeptidase 3 | [139] |
| *LAPTM4A* | ENSBTAG00000020894 | Lysosomal protein transmembrane 4 alpha | [140] |
| *LAPTM5* | ENSBTAG00000005477 | Lysosomal protein transmembrane 5 | [51] |
| *LARP7* | ENSBTAG00000009632 | La ribonucleoprotein 7, transcriptional regulator | [139] |
| *LASP1* | ENSBTAG00000030587 | LIM and SH3 protein 1 | [204] |
| *LAT2* | ENSBTAG00000045767 | Linker for activation of T cells family member 2 | [51] |
| *LBP* | ENSBTAG00000016864 | Lipopolysaccharide binding protein | [138] |
| *LCLAT1* | ENSBTAG00000054158 | Lysocardiolipin acyltransferase 1 | [205] |
| *LCN2* | ENSBTAG00000014149 | Lipocalin 2 | [138] |
| *LCN6* | ENSBTAG00000016144 | Lipocalin 6 | [203] |
| *LCP1* | ENSBTAG00000007079 | Lymphocyte cytosolic protein 1 | [51] |
| *LDAH* | ENSBTAG00000031014 | Lipid droplet associated hydrolase | [203] |
| *LEMD3* | ENSBTAG00000039435 | LEM domain containing 3 | [203] |
| *LEPROT* | ENSBTAG00000005909 | Leptin receptor overlapping transcript | [138] |
| *LETM2* | ENSBTAG00000015444 | LETM1 domain-containing protein LETM2, mitochondrial-like | [203] |
| *LGALS1* | ENSBTAG00000015089 | Galectin 1 | [52] |
| *LGALS3BP* | ENSBTAG00000001368 | Galectin 3 binding protein | [139] |
| *LGALS9* | ENSBTAG00000006846 | Galectin 9 | [139] |
| *LHFPL3* | ENSBTAG00000000189 | LHFPL tetraspan subfamily member 3 | [203] |
| *LHPP* | ENSBTAG00000010957 | Phospholysine phosphohistidine inorganic pyrophosphate phosphatase | [203] |
| *LIF* | ENSBTAG00000007424 | LIF interleukin 6 family cytokine | [203] |
| *LIN52* | ENSBTAG00000030557 | Lin-52 DREAM MuvB core complex component | [203] |
| *LIPE* | ENSBTAG00000038321 | Lipase E, hormone sensitive type | [204] |
| *LIPT1* | ENSBTAG00000003965 | Lipoyltransferase 1 | [140] |
| *LMCD1* | ENSBTAG00000005431 | LIM and cysteine rich domains 1 | [139] |
| *LMNB1* | ENSBTAG00000002882 | Lamin B1 | [139] |
| *LMNB2* | ENSBTAG00000013624 | Lamin B2 | [203] |
| *LMO2* | ENSBTAG00000006951 | LIM domain only 2 | [139] |
| *LOC100298356* | ENSBTAG00000045588 | Bone marrow stromal antigen 2 | [203] |
| *LOC407171* | ENSBTAG00000004043 | Fc gamma 2 receptor | [51] |
| *LOC504773* | ENSBTAG00000010155 | Regakine 1 | [52] |
| *LOXL1* | ENSBTAG00000009086 | Lysyl oxidase like 1 | [52] |
| *LOXL4* | ENSBTAG00000020895 | Lysyl oxidase like 4 | [52] |
| *LPAR3* | ENSBTAG00000003791 | Lysophosphatidic acid receptor 3 | [203] |
| *LPIN1* | ENSBTAG00000007689 | Lipin 1 | [204] |
| *LPIN3* | ENSBTAG00000009165 | Lipin 3 | [203] |
| *LPL* | ENSBTAG00000012855 | Lipoprotein lipase | [51] |
| *LPO* | ENSBTAG00000012780 | Lactoperoxidase | [52] |
| *LRATD1* | ENSBTAG00000004948 | LRAT domain containing 1 | [203] |
| *LRIG3* | ENSBTAG00000002227 | Leucine rich repeats and immunoglobulin like domains 3 | [203] |
| *LRP5* | ENSBTAG00000005903 | LDL receptor related protein 5 | [138] |
| *LRRC18* | ENSBTAG00000005996 | Leucine rich repeat containing 18 | [203] |
| *LRRC20* | ENSBTAG00000005666 | Leucine rich repeat containing 20 | [203] |
| *LRRC25* | ENSBTAG00000017273 | Leucine rich repeat containing 25 | [203] |
| *LRRC42* | ENSBTAG00000019198 | Leucine rich repeat containing 42 | [140] |
| *LRRC4C* | ENSBTAG00000007324 | Leucine rich repeat containing 4C | [204] |
| *LRRC58* | ENSBTAG00000033170 | Leucine rich repeat containing 58 | [203] |
| *LRRC75A* | ENSBTAG00000025078 | Leucine rich repeat containing 75A | [203] |
| *LRRC8D* | ENSBTAG00000020958 | Leucine rich repeat containing 8 VRAC subunit D | [140] |
| *LRRK2* | ENSBTAG00000016260 | Leucine rich repeat kinase 2 | [138] |
| *LRRN3* | ENSBTAG00000004399 | Leucine rich repeat neuronal 3 | [138] |
| *LSAMP* | ENSBTAG00000049383 | Limbic system associated membrane protein | [203] |
| *LTB* | ENSBTAG00000020674 | Lymphotoxin beta | [139] |
| *LTBP2* | ENSBTAG00000021957 | Latent transforming growth factor beta binding protein 2 | [203] |
| *LTBP3* | ENSBTAG00000006747 | Latent transforming growth factor beta binding protein 3 | [203] |
| *LTBP4* | ENSBTAG00000004757 | Latent transforming growth factor beta binding protein 4 | [138] |
| *LUM* | ENSBTAG00000001745 | Lumican | [52] |
| *LUZP1* | ENSBTAG00000009502 | Leucine zipper protein 1 | [203] |
| *LXN* | ENSBTAG00000009336 | Latexin | [140] |
| *LYN* | ENSBTAG00000020034 | LYN proto-oncogene, Src family tyrosine kinase | [139] |
| *LYPLAL1* | ENSBTAG00000015534 | Lysophospholipase like 1 | [138] |
| *LYRM4* | ENSBTAG00000031432 | LYR motif containing 4 | [138] |
| *LYZ* | ENSBTAG00000026779 | Lysozyme | [51] |
| *LYZ2* | ENSBTAG00000026088 | Lysozyme C-2 | [52] |
| *LYZL1* | ENSBTAG00000010330 | Lysozyme-like 1 | [205] |
| *LYZL6* | ENSBTAG00000000029 | Lysozyme like 6 | [203] |
| *MAB21L1* | ENSBTAG00000034069 | Mab-21 like 1 | [139] |
| *MAD2L1BP* | ENSBTAG00000009548 | MAD2L1 binding protein | [203] |
| *MADCAM1* | ENSBTAG00000039355 | Mucosal vascular addressin cell adhesion molecule 1 | [205] |
| *MAFF* | ENSBTAG00000021435 | MAF bZIP transcription factor F | [139] |
| *MAGEE2* | ENSBTAG00000025062 | MAGE family member E2 | [203] |
| *MAML3* | ENSBTAG00000011373 | Mastermind like transcriptional coactivator 3 | [203] |
| *MANSC1* | ENSBTAG00000008721 | MANSC domain containing 1 | [139] |
| *MAP1B* | ENSBTAG00000001961 | Microtubule associated protein 1B | [203] |
| *MAP3K8* | ENSBTAG00000011600 | Mitogen-activated protein kinase kinase kinase 8 | [51] |
| *MAP4K5* | ENSBTAG00000014792 | Mitogen-activated protein kinase kinase kinase kinase 5 | [203] |
| *MAP6* | ENSBTAG00000001028 | Microtubule associated protein 6 | [138] |
| *MAP7D1* | ENSBTAG00000030301 | MAP7 domain containing 1 | [204] |
| *MAPK1IP1L* | ENSBTAG00000021986 | Mitogen-activated protein kinase 1 interacting protein 1 like | [140] |
| *MAPK3* | ENSBTAG00000016156 | Mitogen-activated protein kinase 3 | [203] |
| *MAPK7* | ENSBTAG00000001014 | Mitogen-activated protein kinase 7 | [203] |
| *MAPK8* | ENSBTAG00000007876 | Mitogen-activated protein kinase 8 | [203] |
| *MAPRE1* | ENSBTAG00000003908 | Microtubule associated protein RP/EB family member 1 | [138] |
| *MAPRE3* | ENSBTAG00000008884 | Microtubule associated protein RP/EB family member 3 | [138] |
| *MARCH7* | ENSBTAG00000008755 | Membrane associated ring-CH-type finger 7 | [140] |
| *MARCHF6* | ENSBTAG00000003536 | Membrane associated ring-CH-type finger 6 | [203] |
| *MARCKSL1* | ENSBTAG00000046862 | MARCKS like 1 | [51] |
| *MARVELD1* | ENSBTAG00000013478 | MARVEL domain containing 1 | [203] |
| *MAT2A* | ENSBTAG00000020873 | Methionine adenosyltransferase 2A | [140] |
| *MATN1* | ENSBTAG00000003479 | Matrilin 1 | [203] |
| *MATN3* | ENSBTAG00000020893 | Matrilin 3 | [203] |
| *MAX* | ENSBTAG00000017994 | MYC associated factor X | [138] |
| *MBP* | ENSBTAG00000022890 | Myelin basic protein | [51] |
| *MCAM* | ENSBTAG00000006835 | Melanoma cell adhesion molecule | [138] |
| *MCM3AP* | ENSBTAG00000003148 | Minichromosome maintenance complex component 3 associated protein | [203] |
| *MCM4* | ENSBTAG00000017021 | Minichromosome maintenance complex component 4 | [139] |
| *MCM5* | ENSBTAG00000015595 | Minichromosome maintenance complex component 5 | [139] |
| *MCM6* | ENSBTAG00000015172 | Minichromosome maintenance complex component 6 | [140] |
| *MCOLN3* | ENSBTAG00000016982 | Mucolipin 3 | [203] |
| *MCTP2* | ENSBTAG00000013689 | Multiple C2 and transmembrane domain containing 2 | [51] |
| *MDK* | ENSBTAG00000007740 | Midkine | [139] |
| *MDP1* | ENSBTAG00000002712 | Magnesium dependent phosphatase 1 | [203] |
| *ME2* | ENSBTAG00000016269 | Malic enzyme 2 | [51] |
| *MECOM* | ENSBTAG00000005871 | MDS1 and EVI1 complex locus | [138] |
| *MED18* | ENSBTAG00000002678 | Mediator complex subunit 18 | [203] |
| *MED20* | ENSBTAG00000010100 | Mediator complex subunit 20 | [138] |
| *MEDAG* | ENSBTAG00000008271 | Mesenteric estrogen dependent adipogenesis | [203] |
| *MEF2C* | ENSBTAG00000020701 | Myocyte enhancer factor 2C | [203] |
| *MEFV* | ENSBTAG00000019123 | MEFV innate immuity regulator, pyrin | [203] |
| *MEI1* | ENSBTAG00000004488 | Meiotic double-stranded break formation protein 1 | [203] |
| *MEIS2* | ENSBTAG00000003172 | Meis homeobox 2 | [139] |
| *MEMO1* | ENSBTAG00000007734 | Mediator of cell motility 1 | [140] |
| *MEOX1* | ENSBTAG00000015751 | Mesenchyme homeobox 1 | [203] |
| *METTL15* | ENSBTAG00000003361 | Methyltransferase like 15 | [203] |
| *METTL4* | ENSBTAG00000021668 | Methyltransferase like 4 | [203] |
| *MFAP4* | ENSBTAG00000006187 | Microfibril associated protein 4 | [52] |
| *MFGE8* | ENSBTAG00000003300 | Milk fat globule EGF and factor V/VIII domain containing | [52] |
| *MFSD13A* | ENSBTAG00000021065 | Major facilitator superfamily domain containing 13A | [203] |
| *MFSD4A* | ENSBTAG00000004952 | Major facilitator superfamily domain containing 4A | [204] |
| *MFSD6L* | ENSBTAG00000039012 | Major facilitator superfamily domain containing 6 like | [203] |
| *MFSD9* | ENSBTAG00000001714 | Major facilitator superfamily domain containing 9 | [203] |
| *MGAT2* | ENSBTAG00000033891 | Alpha-1,6-mannosyl-glycoprotein 2-beta-N-acetylglucosaminyltransferase | [140] |
| *MGAT4C* | ENSBTAG00000011153 | MGAT4 family member C | [203] |
| *MGC152281* | ENSBTAG00000037856 | Uncharacterized LOC507942 | [140] |
| *MGEA5* | ENSBTAG00000016336 | O-GlcNAcase | [138] |
| *MGP* | ENSBTAG00000012370 | Matrix Gla protein | [203] |
| *MIA2* | ENSBTAG00000006819 | MIA SH3 domain ER export factor 2 | [203] |
| *MID1* | ENSBTAG00000010152 | Midline 1 | [138] |
| *MID1IP1* | ENSBTAG00000011463 | MID1 interacting protein 1 | [204] |
| *MINDY2* | ENSBTAG00000012391 | MINDY lysine 48 deubiquitinase 2 | [203] |
| *MIOS* | ENSBTAG00000013781 | Meiosis regulator for oocyte development | [203] |
| *MIPOL1* | ENSBTAG00000000655 | Mirror-image polydactyly 1 | [139] |
| *MIR16A* | ENSBTAG00000036389 | Bta-mir-16a | [203] |
| *MIR2284S* | ENSBTAG00000044565 | Bta-mir-2284s | [203] |
| *MIR2285C* | ENSBTAG00000050027 | Bta-mir-2285c | [203] |
| *MIR2436* | ENSBTAG00000045137 | Bta-mir-2436 | [203] |
| *MIR6530* | ENSBTAG00000052061 | Bta-mir-6530 | [203] |
| *MIR7858* | ENSBTAG00000048923 | Bta-mir-7858 | [203] |
| *MIR7859* | ENSBTAG00000049659 | Bta-mir-7859 | [203] |
| *MIS18BP1* | ENSBTAG00000002613 | MIS18 binding protein 1 | [203] |
| *MKI67* | ENSBTAG00000002444 | Marker of proliferation Ki-67 | [139] |
| *MKLN1* | ENSBTAG00000010437 | Muskelin 1 | [138] |
| *MKNK1* | ENSBTAG00000019933 | MAPK interacting serine/threonine kinase 1 | [51] |
| *MLEC* | ENSBTAG00000020050 | Malectin | [204] |
| *MLIP* | ENSBTAG00000014581 | Muscular LMNA interacting protein | [203] |
| *MLLT11* | ENSBTAG00000015369 | MLLT11 transcription factor 7 cofactor | [139] |
| *MMP1* | ENSBTAG00000048029 | Matrix metallopeptidase 1 | [139] |
| *MMP12* | ENSBTAG00000053542 | Matrix metallopeptidase 12 | [203] |
| *MMP13* | ENSBTAG00000015059 | Matrix metallopeptidase 13 | [139] |
| *MMP15* | ENSBTAG00000012919 | Matrix metallopeptidase 15 | [203] |
| *MMP19* | ENSBTAG00000009051 | Matrix metallopeptidase 19 | [51] |
| *MMP2* | ENSBTAG00000019267 | Matrix metallopeptidase 2 | [138] |
| *MMP9* | ENSBTAG00000020676 | Matrix metallopeptidase 9 | [51] |
| *MOB1A* | ENSBTAG00000006259 | MOB kinase activator 1A | [203] |
| *MOBP* | ENSBTAG00000009774 | Myelin associated oligodendrocyte basic protein | [203] |
| *MOCOS* | ENSBTAG00000012252 | Molybdenum cofactor sulfurase | [138] |
| *MOG* | ENSBTAG00000017818 | Myelin oligodendrocyte glycoprotein | [203] |
| *MORF4L2* | ENSBTAG00000001311 | Mortality factor 4 like 2 | [139] |
| *MORN3* | ENSBTAG00000004458 | MORN repeat containing 3 | [203] |
| *MOSC2* | ENSBTAG00000016277 | Mitochondrial amidoxime reducing component 2 | [140] |
| *MOV10* | ENSBTAG00000014297 | Mov10 RISC complex RNA helicase | [140] |
| *MPHOSPH10* | ENSBTAG00000009854 | M-phase phosphoprotein 10 | [138] |
| *MPPE1* | ENSBTAG00000002128 | Metallophosphoesterase 1 | [203] |
| *MPTX* | ENSBTAG00000012884 | Putative mucosal pentraxin homolog | [203] |
| *MRAP2* | ENSBTAG00000000257 | Melanocortin 2 receptor accessory protein 2 | [203] |
| *MRCL* | ENSBTAG00000004766 | C-type lectin domain family 18 member C | [203] |
| *MREG* | ENSBTAG00000013215 | Melanoregulin | [139] |
| *MRGPRX2* | ENSBTAG00000002245 | MAS related GPR family member X2 | [203] |
| *MROH1* | ENSBTAG00000014458 | Maestro heat like repeat family member 1 | [203] |
| *MROH9* | ENSBTAG00000006408 | Maestro heat like repeat family member 9 | [203] |
| *MRPL18* | ENSBTAG00000001289 | Mitochondrial ribosomal protein L18 | [138] |
| *MRPL32* | ENSBTAG00000000991 | Mitochondrial ribosomal protein L32 | [204] |
| *MRPS27* | ENSBTAG00000001962 | Mitochondrial ribosomal protein S27 | [138] |
| *MRPS36* | ENSBTAG00000011045 | Mitochondrial ribosomal protein S36 | [203] |
| *MS4A8* | ENSBTAG00000009289 | Membrane spanning 4-domains A8 | [51] |
| *M-SAA32* | ENSBTAG00000054278 | Mammary serum amyloid A3.2 | [203] |
| *MSR1* | ENSBTAG00000002885 | Macrophage scavenger receptor 1 | [52] |
| *MSRB1* | ENSBTAG00000049306 | Methionine sulfoxide reductase B1 | [203] |
| *MSTN* | ENSBTAG00000011808 | Myostatin | [52] |
| *MSTO1* | ENSBTAG00000026613 | Misato mitochondrial distribution and morphology regulator 1 | [203] |
| *MSX1* | ENSBTAG00000010875 | Msh homeobox 1 | [139] |
| *MT1A* | ENSBTAG00000054808 | Metallothionein-1A | [51] |
| *MT1E* | ENSBTAG00000001595 | Metallothionein 1E | [51] |
| *MT2A* | ENSBTAG00000023659 | Metallothionein 2A | [51] |
| *MT3* | ENSBTAG00000016886 | Metallothionein 3 | [140] |
| *MTHFD1L* | ENSBTAG00000009846 | Methylenetetrahydrofolate dehydrogenase (NADP+ dependent) 1 like | [203] |
| *MTHFS* | ENSBTAG00000020023 | 5,10-methenyltetrahydrofolate synthetase (5-formyltetrahydrofolate cyclo-ligase) | [138] |
| *MTMR2* | ENSBTAG00000016557 | Myotubularin related protein 2 | [203] |
| *MTMR3* | ENSBTAG00000001030 | Myotubularin related protein 3 | [203] |
| *MTPN* | ENSBTAG00000007806 | Myotrophin | [140] |
| *MUC1* | ENSBTAG00000017104 | Mucin 1, cell surface associated | [139] |
| *MX1* | ENSBTAG00000030913 | MX dynamin like GTPase 1 | [51] |
| *MXD3* | ENSBTAG00000005010 | MAX dimerization protein 3 | [139] |
| *MXI1* | ENSBTAG00000017263 | MAX interactor 1, dimerization protein | [139] |
| *MXRA5* | ENSBTAG00000022150 | Matrix remodeling associated 5 | [139] |
| *MYB* | ENSBTAG00000012074 | MYB proto-oncogene, transcription factor | [140] |
| *MYCBP2* | ENSBTAG00000018851 | MYC binding protein 2 | [140] |
| *MYH11* | ENSBTAG00000015988 | Myosin heavy chain 11 | [204] |
| *MYH9* | ENSBTAG00000010402 | Myosin heavy chain 9 | [203] |
| *MYL6B* | ENSBTAG00000031217 | Myosin light chain 6B | [140] |
| *MYL9* | ENSBTAG00000011473 | Myosin light chain 9 | [204] |
| *MYO1B* | ENSBTAG00000011256 | Myosin IB | [203] |
| *MYO1F* | ENSBTAG00000007661 | Myosin IF | [51] |
| *MYO3B* | ENSBTAG00000003626 | Myosin IIIB | [203] |
| *MYO9B* | ENSBTAG00000011125 | Myosin IXB | [204] |
| *MYOC* | ENSBTAG00000020342 | Myocilin | [52] |
| *MYOF* | ENSBTAG00000016918 | Myoferlin | [203] |
| *MYOM1* | ENSBTAG00000019585 | Myomesin 1 | [203] |
| *MYOM2* | ENSBTAG00000009387 | Myomesin 2 | [203] |
| *MYOT* | ENSBTAG00000007782 | Myotilin | [203] |
| *NAA25* | ENSBTAG00000006666 | N-alpha-acetyltransferase 25, NatB auxiliary subunit | [203] |
| *NAALADL2* | ENSBTAG00000048493 | N-acetylated alpha-linked acidic dipeptidase like 2 | [139] |
| *NABP1* | ENSBTAG00000018653 | Nucleic acid binding protein 1 | [203] |
| *NAMPT* | ENSBTAG00000015509 | Nicotinamide phosphoribosyltransferase | [139] |
| *NAV2* | ENSBTAG00000018431 | Neuron navigator 2 | [203] |
| *NBEAL1* | ENSBTAG00000014469 | Neurobeachin like 1 | [203] |
| *NCALD* | ENSBTAG00000026963 | Neurocalcin delta | [203] |
| *NCAM1* | ENSBTAG00000005710 | Neural cell adhesion molecule 1 | [203] |
| *NCAPG2* | ENSBTAG00000016131 | Non-SMC condensin II complex subunit G2 | [139] |
| *NCF2* | ENSBTAG00000008004 | Neutrophil cytosolic factor 2 | [51] |
| *NCKAP1L* | ENSBTAG00000019686 | NCK associated protein 1 like | [51] |
| *NCOA2* | ENSBTAG00000020312 | Nuclear receptor coactivator 2 | [203] |
| *NCOA4* | ENSBTAG00000019565 | Nuclear receptor coactivator 4 | [140] |
| *NCOA6* | ENSBTAG00000007930 | Nuclear receptor coactivator 6 | [203] |
| *NCOA7* | ENSBTAG00000046503 | Nuclear receptor coactivator 7 | [140] |
| *ND2* | ENSBTAG00000043571 | NADH dehydrogenase subunit 2 | [138] |
| *ND3* | ENSBTAG00000043568 | NADH dehydrogenase subunit 3 | [138] |
| *NDE1* | ENSBTAG00000015986 | NudE neurodevelopment protein 1 | [138] |
| *NDFIP2* | ENSBTAG00000014227 | Nedd4 family interacting protein 2 | [203] |
| *NDP* | ENSBTAG00000012623 | Norrin cystine knot growth factor NDP | [204] |
| *NDUFA11* | ENSBTAG00000019025 | NADH:ubiquinone oxidoreductase subunit A11 | [203] |
| *NDUFA8* | ENSBTAG00000004295 | NADH:ubiquinone oxidoreductase subunit A8 | [140] |
| *NDUFAB1* | ENSBTAG00000006391 | NADH:ubiquinone oxidoreductase subunit AB1 | [138] |
| *NDUFB8* | ENSBTAG00000000091 | NADH:ubiquinone oxidoreductase subunit B8 | [138] |
| *NECTIN1* | ENSBTAG00000015939 | Nectin cell adhesion molecule 1 | [203] |
| *NECTIN2* | ENSBTAG00000015318 | Nectin cell adhesion molecule 2 | [203] |
| *NEFM* | ENSBTAG00000039530 | Neurofilament medium | [138] |
| *NEIL1* | ENSBTAG00000007365 | Nei like DNA glycosylase 1 | [138] |
| *NEK9* | ENSBTAG00000016612 | NIMA related kinase 9 | [203] |
| *NELFA* | ENSBTAG00000017921 | Negative elongation factor complex member A | [203] |
| *NELFE* | ENSBTAG00000007453 | Negative elongation factor complex member E | [203] |
| *NEUROD6* | ENSBTAG00000000558 | Neuronal differentiation 6 | [203] |
| *NEUROG2* | ENSBTAG00000015259 | Neurogenin 2 | [203] |
| *NFAM1* | ENSBTAG00000005018 | NFAT activating protein with ITAM motif 1 | [51] |
| *NFE2* | ENSBTAG00000001562 | Nuclear factor, erythroid 2 | [51] |
| *NFKB1* | ENSBTAG00000020270 | Nuclear factor kappa B subunit 1 | [51] |
| *NFKB2* | ENSBTAG00000006017 | Nuclear factor kappa B subunit 2 | [51] |
| *NFKBIA* | ENSBTAG00000016683 | NFKB inhibitor alpha | [138] |
| *NFKBIB* | ENSBTAG00000001778 | NFKB inhibitor beta | [140] |
| *NFKBID* | ENSBTAG00000007334 | NFKB inhibitor delta | [203] |
| *NFKBIE* | ENSBTAG00000013705 | NFKB inhibitor epsilon | [139] |
| *NFKBIZ* | ENSBTAG00000010987 | NFKB inhibitor zeta | [139] |
| *NGB* | ENSBTAG00000016285 | Neuroglobin | [203] |
| *NGF* | ENSBTAG00000007446 | Nerve growth factor | [139] |
| *NGP* | ENSBTAG00000031519 | Neutrophilic granule protein-like | [203] |
| *NHEJ1* | ENSBTAG00000031041 | Non-homologous end joining factor 1 | [205] |
| *NHS* | ENSBTAG00000039195 | NHS actin remodeling regulator | [203] |
| *NID1* | ENSBTAG00000007244 | Nidogen 1 | [139] |
| *NIFK* | ENSBTAG00000019387 | Nucleolar protein interacting with the FHA domain of MKI67 | [203] |
| *NIN* | ENSBTAG00000020281 | Ninein | [140] |
| *NIPAL2* | ENSBTAG00000018206 | NIPA like domain containing 2 | [138] |
| *NLF1* | ENSBTAG00000020814 | C2 calcium dependent domain containing 4B | [51] |
| *NLRP3* | ENSBTAG00000001273 | NLR family pyrin domain containing 3 | [203] |
| *NOC2L* | ENSBTAG00000016528 | NOC2 like nucleolar associated transcriptional repressor | [204] |
| *NOL4L* | ENSBTAG00000008348 | Nucleolar protein 4 like | [203] |
| *NOL8* | ENSBTAG00000018613 | Nucleolar protein 8 | [140] |
| *NOP14* | ENSBTAG00000001504 | NOP14 nucleolar protein | [138] |
| *NOP16* | ENSBTAG00000010734 | NOP16 nucleolar protein | [140] |
| *NOS2* | ENSBTAG00000006894 | Nitric oxide synthase 2 | [139] |
| *NOSTRIN* | ENSBTAG00000010362 | Nitric oxide synthase trafficking | [203] |
| *NOX3* | ENSBTAG00000013551 | NADPH oxidase 3 | [203] |
| *NPBWR1* | ENSBTAG00000016159 | Neuropeptides B and W receptor 1 | [203] |
| *NPBWR2* | ENSBTAG00000047704 | Neuropeptides B and W receptor 2 | [203] |
| *NPC2* | ENSBTAG00000021955 | NPC intracellular cholesterol transporter 2 | [203] |
| *NPR2* | ENSBTAG00000011434 | Natriuretic peptide receptor 2 | [204] |
| *NR1I3* | ENSBTAG00000009215 | Nuclear receptor subfamily 1 group I member 3 | [203] |
| *NR4A1* | ENSBTAG00000000507 | Nuclear receptor subfamily 4 group A member 1 | [139] |
| *NR4A3* | ENSBTAG00000001864 | Nuclear receptor subfamily 4 group A member 3 | [139] |
| *NRAS* | ENSBTAG00000046797 | NRAS proto-oncogene, GTPase | [203] |
| *NRDC* | ENSBTAG00000021174 | Nardilysin convertase | [203] |
| *NRXN2* | ENSBTAG00000000244 | Neurexin 2 | [138] |
| *NRXN3* | ENSBTAG00000025324 | Neurexin 3 | [203] |
| *NSD2* | ENSBTAG00000007986 | Nuclear receptor binding SET domain protein 2 | [203] |
| *NSRP1* | ENSBTAG00000019341 | Nuclear speckle splicing regulatory protein 1 | [203] |
| *NT5C3B* | ENSBTAG00000011456 | 5,-nucleotidase, cytosolic IIIB | [138] |
| *NT5E* | ENSBTAG00000048655 | 5,-nucleotidase ecto | [138] |
| *NTM* | ENSBTAG00000010032 | Neurotrimin | [203] |
| *NTN4* | ENSBTAG00000003183 | Netrin 4 | [52] |
| *NUB1* | ENSBTAG00000004540 | Negative regulator of ubiquitin like proteins 1 | [140] |
| *NUCB1* | ENSBTAG00000002378 | Nucleobindin 1 | [138] |
| *NUCB2* | ENSBTAG00000017468 | Nucleobindin 2 | [139] |
| *NUDT11* | ENSBTAG00000005252 | Nudix hydrolase 11 | [203] |
| *NUFIP1* | ENSBTAG00000009415 | Nuclear FMR1 interacting protein 1 | [138] |
| *NUMB* | ENSBTAG00000011683 | NUMB endocytic adaptor protein | [203] |
| *NUMBL* | ENSBTAG00000019461 | NUMB like endocytic adaptor protein | [203] |
| *NUP93* | ENSBTAG00000006611 | Nucleoporin 93 | [140] |
| *NUPR1* | ENSBTAG00000018016 | Nuclear protein 1, transcriptional regulator | [139] |
| *NUSAP1* | ENSBTAG00000010774 | Nucleolar and spindle associated protein 1 | [139] |
| *NXPE3* | ENSBTAG00000013465 | Neurexophilin and PC-esterase domain family member 3 | [203] |
| *NXPH1* | ENSBTAG00000006924 | Neurexophilin 1 | [138] |
| *NXPH4* | ENSBTAG00000047650 | Neurexophilin 4 | [203] |
| *NYAP2* | ENSBTAG00000019152 | Neuronal tyrosine-phosphorylated phosphoinositide-3-kinase adaptor 2 | [203] |
| *OAF* | ENSBTAG00000021338 | Out at first homolog | [204] |
| *OBI1* | ENSBTAG00000000869 | ORC ubiquitin ligase 1 | [203] |
| *OCLN* | ENSBTAG00000000561 | Occludin | [203] |
| *OGFOD1* | ENSBTAG00000001171 | 2-oxoglutarate and iron dependent oxygenase domain containing 1 | [140] |
| *OGN* | ENSBTAG00000011824 | Osteoglycin | [52] |
| *OIP5* | ENSBTAG00000010766 | Opa interacting protein 5 | [139] |
| *OLFM4* | ENSBTAG00000022779 | Olfactomedin 4 | [51] |
| *OLFML2A* | ENSBTAG00000004848 | Olfactomedin like 2A | [139] |
| *OLR1* | ENSBTAG00000004547 | ENSBTAG00000004547 | [139] |
| *OPA1* | ENSBTAG00000019514 | OPA1 mitochondrial dynamin like GTPase | [139] |
| *OPALIN* | ENSBTAG00000011740 | Oligodendrocytic myelin paranodal and inner loop protein | [203] |
| *OPN4* | ENSBTAG00000032800 | Opsin 4 | [203] |
| *OPTN* | ENSBTAG00000016401 | Optineurin | [139] |
| *OR10A6* | ENSBTAG00000053062 | Olfactory receptor family 10 subfamily A member 6 | [203] |
| *OR10R2* | ENSBTAG00000017778 | Olfactory receptor family 10 subfamily R member 2 | [203] |
| *OR1D3B* | ENSBTAG00000052339 | Olfactory receptor family 1 subfamily D member 3B | [203] |
| *OR2Y1* | ENSBTAG00000038629 | Olfactory receptor 2Y1 | [203] |
| *OR2Z1* | ENSBTAG00000051841 | Olfactory receptor 2Z1 | [203] |
| *OR52H1* | ENSBTAG00000048742 | Olfactory receptor family 52 subfamily H member 1 | [203] |
| *OR5M9* | ENSBTAG00000049790 | Olfactory receptor family 5 subfamily M member 9 | [203] |
| *ORAI1* | ENSBTAG00000004457 | ORAI calcium release-activated calcium modulator 1 | [203] |
| *ORC1* | ENSBTAG00000002719 | Origin recognition complex subunit 1 | [139] |
| *ORM1* | ENSBTAG00000017294 | Orosomucoid 1 | [52] |
| *OSBP2* | ENSBTAG00000019146 | Oxysterol binding protein 2 | [203] |
| *OSBPL7* | ENSBTAG00000013523 | Oxysterol binding protein like 7 | [138] |
| *OSCAR* | ENSBTAG00000001051 | Osteoclast associated Ig-like receptor | [140] |
| *OTC* | ENSBTAG00000001488 | Ornithine carbamoyltransferase | [203] |
| *OTOP1* | ENSBTAG00000003651 | Otopetrin 1 | [203] |
| *P2RX4* | ENSBTAG00000010812 | Purinergic receptor P2X 4 | [140] |
| *P2RX5* | ENSBTAG00000015258 | Purinergic receptor P2X 5 | [203] |
| *P2RY12* | ENSBTAG00000015837 | Purinergic receptor P2Y12 | [203] |
| *P4HA1* | ENSBTAG00000032996 | Prolyl 4-hydroxylase subunit alpha 1 | [139] |
| *PAFAH1B2* | ENSBTAG00000005627 | Platelet activating factor acetylhydrolase 1b catalytic subunit 2 | [204] |
| *PAG1* | ENSBTAG00000014026 | Phosphoprotein membrane anchor with glycosphingolipid microdomains 1 | [203] |
| *PAG11* | ENSBTAG00000022759 | Pregnancy-associated glycoprotein 11 | [203] |
| *PAG19* | ENSBTAG00000054803 | Pregnancy-associated glycoprotein 19 | [203] |
| *PAG6* | ENSBTAG00000006304 | Pregnancy-associated glycoprotein 6 | [203] |
| *PAOX* | ENSBTAG00000018321 | Polyamine oxidase | [138] |
| *PAPPA* | ENSBTAG00000004010 | Pappalysin 1 | [203] |
| *PARP12* | ENSBTAG00000016546 | Poly(ADP-ribose) polymerase family member 12 | [139] |
| *PARP14* | ENSBTAG00000016656 | Poly(ADP-ribose) polymerase family member 14 | [139] |
| *PARP8* | ENSBTAG00000004066 | Poly(ADP-ribose) polymerase family member 8 | [140] |
| *PARP9* | ENSBTAG00000021791 | Poly(ADP-ribose) polymerase family member 9 | [139] |
| *PARS2* | ENSBTAG00000040313 | Prolyl-tRNA synthetase 2, mitochondrial | [203] |
| *PARVB* | ENSBTAG00000021978 | Parvin beta | [203] |
| *PARVG* | ENSBTAG00000008057 | Parvin gamma | [203] |
| *PAX6* | ENSBTAG00000004561 | Paired box 6 | [203] |
| *PBX2* | ENSBTAG00000014421 | PBX homeobox 2 | [203] |
| *PC* | ENSBTAG00000019700 | Pyruvate carboxylase | [138] |
| *PCBP2* | ENSBTAG00000020757 | Poly(rC) binding protein 2 | [140] |
| *PCDH19* | ENSBTAG00000014169 | Protocadherin 19 | [138] |
| *PCDH9* | ENSBTAG00000019340 | Protocadherin 9 | [203] |
| *PCED1A* | ENSBTAG00000003863 | PC-esterase domain containing 1A | [203] |
| *PCGF1* | ENSBTAG00000016606 | Polycomb group ring finger 1 | [138] |
| *PCIF1* | ENSBTAG00000010126 | PDX1 C-terminal inhibiting factor 1 | [203] |
| *PCLAF* | ENSBTAG00000039462 | PCNA clamp associated factor | [203] |
| *PCNA* | ENSBTAG00000006065 | Proliferating cell nuclear antigen | [139] |
| *PCNX2* | ENSBTAG00000000535 | Pecanex 2 | [138] |
| *PCP4* | ENSBTAG00000051421 | Purkinje cell protein 4 | [203] |
| *PDE10A* | ENSBTAG00000007758 | Phosphodiesterase 10A | [203] |
| *PDE1A* | ENSBTAG00000012100 | Phosphodiesterase 1A | [203] |
| *PDE1C* | ENSBTAG00000002739 | Phosphodiesterase 1C | [203] |
| *PDE4B* | ENSBTAG00000008636 | Phosphodiesterase 4B | [51] |
| *PDE4C* | ENSBTAG00000010652 | Phosphodiesterase 4C | [203] |
| *PDE8A* | ENSBTAG00000015909 | Phosphodiesterase 8A | [203] |
| *PDGFB* | ENSBTAG00000021697 | Platelet derived growth factor subunit B | [139] |
| *PDHB* | ENSBTAG00000021724 | Pyruvate dehydrogenase E1 subunit beta | [140] |
| *PDIA3* | ENSBTAG00000017196 | Protein disulfide isomerase family A member 3 | [138] |
| *PDIA4* | ENSBTAG00000017143 | Protein disulfide isomerase family A member 4 | [138] |
| *PDLIM5* | ENSBTAG00000012818 | PDZ and LIM domain 5 | [203] |
| *PDPK1* | ENSBTAG00000034436 | 3-phosphoinositide dependent protein kinase 1 | [204] |
| *PDS5A* | ENSBTAG00000017809 | PDS5 cohesin associated factor A | [140] |
| *PDZD3* | ENSBTAG00000012507 | PDZ domain containing 3 | [203] |
| *PDZD8* | ENSBTAG00000011401 | PDZ domain containing 8 | [203] |
| *PER1* | ENSBTAG00000003889 | Period circadian regulator 1 | [204] |
| *PEX13* | ENSBTAG00000005257 | Peroxisomal biogenesis factor 13 | [203] |
| *PEX6* | ENSBTAG00000005532 | Peroxisomal biogenesis factor 6 | [203] |
| *PFKFB2* | ENSBTAG00000002126 | 6-phosphofructo-2-kinase/fructose-2,6-biphosphatase 2 | [203] |
| *PFKFB3* | ENSBTAG00000008401 | 6-phosphofructo-2-kinase/fructose-2,6-biphosphatase 3 | [139] |
| *PGA5* | ENSBTAG00000014761 | Pepsinogen 5, group I (pepsinogen A) | [51] |
| *PGBD1* | ENSBTAG00000046573 | PiggyBac transposable element derived 1 | [203] |
| *PGF* | ENSBTAG00000013688 | Placental growth factor | [203] |
| *PGK1* | ENSBTAG00000000894 | Phosphoglycerate kinase 1 | [138] |
| *PGLYRP3* | ENSBTAG00000021443 | Peptidoglycan recognition protein 3 | [203] |
| *PGM1* | ENSBTAG00000019011 | Phosphoglucomutase 1 | [203] |
| *PGM5* | ENSBTAG00000033190 | Phosphoglucomutase 5 | [139] |
| *PGS1* | ENSBTAG00000000675 | Phosphatidylglycerophosphate synthase 1 | [51] |
| *PHACTR1* | ENSBTAG00000012039 | Phosphatase and actin regulator 1 | [203] |
| *PHB* | ENSBTAG00000017120 | Prohibitin | [138] |
| *PHB2* | ENSBTAG00000013123 | Prohibitin 2 | [140] |
| *PHC3* | ENSBTAG00000013938 | Polyhomeotic homolog 3 | [140] |
| *PHF11* | ENSBTAG00000013159 | PHD finger protein 11 | [203] |
| *PHF20* | ENSBTAG00000005571 | PHD finger protein 20 | [203] |
| *PHKA2* | ENSBTAG00000015355 | Phosphorylase kinase regulatory subunit alpha 2 | [203] |
| *PHLPP* | ENSBTAG00000007545 | PH domain and leucine rich repeat protein phosphatase 2 | [204] |
| *PHYHIP* | ENSBTAG00000015757 | Phytanoyl-CoA 2-hydroxylase interacting protein | [203] |
| *PIBF1* | ENSBTAG00000019895 | Progesterone immunomodulatory binding factor 1 | [139] |
| *PICALM* | ENSBTAG00000001657 | Phosphatidylinositol binding clathrin assembly protein | [140] |
| *PIDD1* | ENSBTAG00000019634 | P53-induced death domain protein 1 | [203] |
| *PIF1* | ENSBTAG00000007638 | PIF1 5,-to-3, DNA helicase | [203] |
| *PIGR* | ENSBTAG00000019798 | Polymeric immunoglobulin receptor | [204] |
| *PIGS* | ENSBTAG00000013098 | Phosphatidylinositol glycan anchor biosynthesis class S | [203] |
| *PIK3AP1* | ENSBTAG00000019872 | Phosphoinositide-3-kinase adaptor protein 1 | [139] |
| *PIK3CB* | ENSBTAG00000006909 | Phosphatidylinositol-4,5-bisphosphate 3-kinase catalytic subunit beta | [203] |
| *PIK3R1* | ENSBTAG00000010989 | Phosphoinositide-3-kinase regulatory subunit 1 | [140] |
| *PIK3R2* | ENSBTAG00000002350 | Phosphoinositide-3-kinase regulatory subunit 2 | [203] |
| *PIK3R5* | ENSBTAG00000005978 | Phosphoinositide-3-kinase regulatory subunit 5 | [51] |
| *PIKFYVE* | ENSBTAG00000002177 | Phosphoinositide kinase, FYVE-type zinc finger containing | [203] |
| *PILRA* | ENSBTAG00000001066 | Paired immunoglobin like type 2 receptor alpha | [203] |
| *PIM1* | ENSBTAG00000000396 | Pim-1 proto-oncogene, serine/threonine kinase | [139] |
| *PIMREG* | ENSBTAG00000002981 | PICALM interacting mitotic regulator | [203] |
| *PIP5K1B* | ENSBTAG00000009917 | Phosphatidylinositol-4-phosphate 5-kinase type 1 beta | [139] |
| *PIP5K1C* | ENSBTAG00000000710 | Phosphatidylinositol-4-phosphate 5-kinase type 1 gamma | [203] |
| *PIR* | ENSBTAG00000009477 | Pirin | [204] |
| *PJA2* | ENSBTAG00000021675 | Praja ring finger ubiquitin ligase 2 | [140] |
| *PKIA* | ENSBTAG00000008150 | CAMP-dependent protein kinase inhibitor alpha | [138] |
| *PKIB* | ENSBTAG00000004394 | CAMP-dependent protein kinase inhibitor beta | [139] |
| *PKN1* | ENSBTAG00000017037 | Protein kinase N1 | [203] |
| *PLA2G12A* | ENSBTAG00000015230 | Phospholipase A2 group XIIA | [140] |
| *PLA2G15* | ENSBTAG00000007512 | Phospholipase A2 group XV | [203] |
| *PLA2G4A* | ENSBTAG00000013298 | Phospholipase A2 group IVA | [203] |
| *PLA2G7* | ENSBTAG00000019315 | Phospholipase A2 group VII | [52] |
| *PLAGL1* | ENSBTAG00000026523 | PLAG1 like zinc finger 1 | [203] |
| *PLAT* | ENSBTAG00000001244 | Plasminogen activator, tissue type | [139] |
| *PLAU* | ENSBTAG00000005947 | Plasminogen activator, urokinase | [51] |
| *PLAUR* | ENSBTAG00000013125 | Plasminogen activator, urokinase receptor | [140] |
| *PLCL1* | ENSBTAG00000007635 | Phospholipase C like 1 (inactive) | [203] |
| *PLCXD1* | ENSBTAG00000035144 | Phosphatidylinositol-specific phospholipase C, X domain containing 1 | [203] |
| *PLCXD3* | ENSBTAG00000010822 | Phosphatidylinositol specific phospholipase C X domain containing 3 | [203] |
| *PLEK* | ENSBTAG00000009658 | Pleckstrin | [51] |
| *PLEKHA4* | ENSBTAG00000009177 | Pleckstrin homology domain containing A4 | [139] |
| *PLEKHG1* | ENSBTAG00000009679 | Pleckstrin homology and RhoGEF domain containing G1 | [203] |
| *PLEKHO1* | ENSBTAG00000004852 | Pleckstrin homology domain containing O1 | [51] |
| *PLEKHO2* | ENSBTAG00000008411 | Pleckstrin homology domain containing O2 | [51] |
| *PLG* | ENSBTAG00000001271 | Plasminogen | [203] |
| *PLK2* | ENSBTAG00000016771 | Polo like kinase 2 | [140] |
| *PLP1* | ENSBTAG00000006977 | Proteolipid protein 1 | [138] |
| *PLSCR1* | ENSBTAG00000031306 | Phospholipid scramblase 1 | [140] |
| *PLSCR2* | ENSBTAG00000022227 | Phospholipid scramblase 1 | [140] |
| *PLSCR4* | ENSBTAG00000011986 | Phospholipid scramblase 4 | [139] |
| *PLXNA4* | ENSBTAG00000014543 | Plexin A4 | [203] |
| *PMEPA1* | ENSBTAG00000054628 | Prostate transmembrane protein, androgen induced 1 | [204] |
| *PML* | ENSBTAG00000015779 | PML nuclear body scaffold | [139] |
| *PMP22* | ENSBTAG00000019070 | Peripheral myelin protein 22 | [138] |
| *PMS1* | ENSBTAG00000018795 | PMS1 homolog 1, mismatch repair system component | [203] |
| *PMVK* | ENSBTAG00000017819 | Phosphomevalonate kinase | [204] |
| *PNISR* | ENSBTAG00000019730 | Arginine/serine-rich protein PNISR | [203] |
| *PNKD* | ENSBTAG00000003936 | PNKD metallo-beta-lactamase domain containing | [203] |
| *PNMA1* | ENSBTAG00000009271 | PNMA family member 1 | [203] |
| *PNPLA1* | ENSBTAG00000015118 | Patatin like phospholipase domain containing 1 | [203] |
| *PNPT1* | ENSBTAG00000008909 | Polyribonucleotide nucleotidyltransferase 1 | [138] |
| *PNRC2* | ENSBTAG00000030435 | Proline rich nuclear receptor coactivator 2 | [140] |
| *PODXL* | ENSBTAG00000010452 | Podocalyxin like | [139] |
| *POLE* | ENSBTAG00000000590 | DNA polymerase epsilon, catalytic subunit | [203] |
| *POLE2* | ENSBTAG00000004931 | DNA polymerase epsilon 2, accessory subunit | [139] |
| *POLH* | ENSBTAG00000006015 | DNA polymerase eta | [139] |
| *POLK* | ENSBTAG00000004028 | DNA polymerase kappa | [139] |
| *POLL* | ENSBTAG00000003576 | DNA polymerase lambda | [203] |
| *POLR1F* | ENSBTAG00000006721 | RNA polymerase I subunit F | [203] |
| *POLR3B* | ENSBTAG00000004781 | RNA polymerase III subunit B | [138] |
| *POMT2* | ENSBTAG00000002704 | Protein O-mannosyltransferase 2 | [204] |
| *POR* | ENSBTAG00000017082 | Cytochrome p450 oxidoreductase | [204] |
| *POSTN* | ENSBTAG00000012409 | Periostin | [52] |
| *POU2F1* | ENSBTAG00000024534 | POU class 2 homeobox 1 | [203] |
| *PPIB* | ENSBTAG00000016822 | Peptidylprolyl isomerase B | [138] |
| *PPIF* | ENSBTAG00000016711 | Peptidylprolyl isomerase F | [140] |
| *PPM1L* | ENSBTAG00000001274 | Protein phosphatase, Mg2+/Mn2+ dependent 1L | [203] |
| *PPOX* | ENSBTAG00000021894 | Protoporphyrinogen oxidase | [140] |
| *PPP1R10* | ENSBTAG00000006933 | Protein phosphatase 1 regulatory subunit 10 | [138] |
| *PPP1R16A* | ENSBTAG00000007834 | Protein phosphatase 1 regulatory subunit 16A | [203] |
| *PPP1R3C* | ENSBTAG00000014831 | Protein phosphatase 1 regulatory subunit 3C | [139] |
| *PPP1R9B* | ENSBTAG00000049864 | Protein phosphatase 1 regulatory subunit 9B | [204] |
| *PPP2R1A* | ENSBTAG00000019851 | Protein phosphatase 2 scaffold subunit A, alpha | [140] |
| *PPP2R3C* | ENSBTAG00000009681 | Protein phosphatase 2 regulatory subunit B, gamma | [139] |
| *PPP3CA* | ENSBTAG00000016005 | Protein phosphatase 3 catalytic subunit alpha | [203] |
| *PPP3CC* | ENSBTAG00000020133 | Protein phosphatase 3 catalytic subunit gamma | [204] |
| *PPP5C* | ENSBTAG00000008014 | Protein phosphatase 5 catalytic subunit | [140] |
| *PRC1* | ENSBTAG00000018643 | Protein regulator of cytokinesis 1 | [139] |
| *PRDM1* | ENSBTAG00000000816 | PR/SET domain 1 | [203] |
| *PRDM5* | ENSBTAG00000012111 | PR/SET domain 5 | [205] |
| *PRDX1* | ENSBTAG00000003642 | Peroxiredoxin 1 | [138] |
| *PRDX3* | ENSBTAG00000008731 | Peroxiredoxin 3 | [203] |
| *PRELP* | ENSBTAG00000017834 | Proline and arginine rich end leucine rich repeat protein | [52] |
| *PRKAA1* | ENSBTAG00000000013 | Protein kinase AMP-activated catalytic subunit alpha 1 | [203] |
| *PRKAG3* | ENSBTAG00000013492 | Protein kinase AMP-activated non-catalytic subunit gamma 3 | [203] |
| *PRKCQ* | ENSBTAG00000010664 | Protein kinase C theta | [203] |
| *PRKCZ* | ENSBTAG00000014119 | Protein kinase C zeta | [203] |
| *PRLHR* | ENSBTAG00000047842 | Prolactin releasing hormone receptor | [203] |
| *PROX2* | ENSBTAG00000015052 | Prospero homeobox 2 | [203] |
| *PRPF31* | ENSBTAG00000007757 | Pre-mRNA processing factor 31 | [203] |
| *PRPF39* | ENSBTAG00000002603 | Pre-mRNA processing factor 39 | [139] |
| *PRPSAP1* | ENSBTAG00000030172 | Phosphoribosyl pyrophosphate synthetase associated protein 1 | [204] |
| *PRR13* | ENSBTAG00000026916 | Proline rich 13 | [51] |
| *PRRC2A* | ENSBTAG00000019682 | Proline rich coiled-coil 2A | [203] |
| *PRRG2* | ENSBTAG00000030563 | Proline rich and Gla domain 2 | [203] |
| *PRSS16* | ENSBTAG00000012461 | Serine protease 16 | [203] |
| *PRSS2* | ENSBTAG00000021565 | Serine protease 2 | [51] |
| *PRSS22* | ENSBTAG00000020582 | Serine protease 22 | [203] |
| *PRSS35* | ENSBTAG00000052055 | Serine protease 35 | [139] |
| *PSEN1* | ENSBTAG00000011757 | Presenilin 1 | [203] |
| *PSMA5* | ENSBTAG00000020641 | Proteasome 20S subunit alpha 5 | [138] |
| *PSMA6* | ENSBTAG00000009683 | Proteasome 20S subunit alpha 6 | [138] |
| *PSMB10* | ENSBTAG00000018040 | Proteasome 20S subunit beta 10 | [51] |
| *PSMB8* | ENSBTAG00000003039 | Proteasome 20S subunit beta 8 | [139] |
| *PSMB9* | ENSBTAG00000008954 | Proteasome 20S subunit beta 9 | [138] |
| *PSMD12* | ENSBTAG00000002423 | Proteasome 26S subunit, non-ATPase 12 | [138] |
| *PSMD2* | ENSBTAG00000005660 | Proteasome 26S subunit, non-ATPase 2 | [140] |
| *PSMD7* | ENSBTAG00000018425 | Proteasome 26S subunit, non-ATPase 7 | [138] |
| *PSME1* | ENSBTAG00000021395 | Proteasome activator subunit 1 | [51] |
| *PSMF1* | ENSBTAG00000018417 | Proteasome inhibitor subunit 1 | [139] |
| *PSMG2* | ENSBTAG00000010552 | Proteasome assembly chaperone 2 | [138] |
| *PSPH* | ENSBTAG00000013081 | Phosphoserine phosphatase | [204] |
| *PSTPIP1* | ENSBTAG00000021144 | Proline-serine-threonine phosphatase interacting protein 1 | [51] |
| *PSTPIP2* | ENSBTAG00000002624 | Proline-serine-threonine phosphatase interacting protein 2 | [51] |
| *PTAFR* | ENSBTAG00000027051 | Platelet activating factor receptor | [138] |
| *PTCH2* | ENSBTAG00000024137 | Patched 2 | [138] |
| *PTGER2* | ENSBTAG00000002163 | Prostaglandin E receptor 2 | [139] |
| *PTGER3* | ENSBTAG00000019230 | Prostaglandin E receptor 3 | [203] |
| *PTGES* | ENSBTAG00000019453 | Prostaglandin E synthase | [139] |
| *PTGFRN* | ENSBTAG00000008022 | Prostaglandin F2 receptor inhibitor | [203] |
| *PTGR2* | ENSBTAG00000003747 | Prostaglandin reductase 2 | [203] |
| *PTGS1* | ENSBTAG00000006716 | Prostaglandin-endoperoxide synthase 1 | [203] |
| *PTGS2* | ENSBTAG00000014127 | Prostaglandin-endoperoxide synthase 2 | [139] |
| *PTHLH* | ENSBTAG00000006538 | Parathyroid hormone like hormone | [139] |
| *PTPN2* | ENSBTAG00000010563 | Protein tyrosine phosphatase non-receptor type 2 | [51] |
| *PTPN22* | ENSBTAG00000019617 | Protein tyrosine phosphatase non-receptor type 22 | [203] |
| *PTPN4* | ENSBTAG00000020855 | Protein tyrosine phosphatase non-receptor type 4 | [203] |
| *PTPN7* | ENSBTAG00000003016 | Protein tyrosine phosphatase non-receptor type 7 | [203] |
| *PTPRD* | ENSBTAG00000010178 | Protein tyrosine phosphatase receptor type D | [203] |
| *PTPRE* | ENSBTAG00000002435 | Protein tyrosine phosphatase receptor type E | [51] |
| *PTPRH* | ENSBTAG00000040559 | Protein tyrosine phosphatase receptor type H | [203] |
| *PTPRK* | ENSBTAG00000020829 | Protein tyrosine phosphatase receptor type K | [203] |
| *PTRH2* | ENSBTAG00000016710 | Peptidyl-tRNA hydrolase 2 | [140] |
| *PTTG1* | ENSBTAG00000012184 | PTTG1 regulator of sister chromatid separation, securin | [139] |
| *PUS10* | ENSBTAG00000009694 | Pseudouridine synthase 10 | [203] |
| *PVALB* | ENSBTAG00000019159 | Parvalbumin | [138] |
| *PVR* | ENSBTAG00000051298 | PVR cell adhesion molecule | [139] |
| *PYCARD* | ENSBTAG00000020535 | PYD and CARD domain containing | [51] |
| *QRICH2* | ENSBTAG00000030173 | Glutamine rich 2 | [203] |
| *QSOX1* | ENSBTAG00000014191 | Quiescin sulfhydryl oxidase 1 | [51] |
| *RAB11FIP1* | ENSBTAG00000011684 | RAB11 family interacting protein 1 | [204] |
| *RAB11FIP2* | ENSBTAG00000007460 | RAB11 family interacting protein 2 | [203] |
| *RAB13* | ENSBTAG00000017604 | RAB13, member RAS oncogene family | [140] |
| *RAB20* | ENSBTAG00000049416 | RAB20, member RAS oncogene family | [203] |
| *RAB27A* | ENSBTAG00000012408 | RAB27A, member RAS oncogene family | [52] |
| *RAB39A* | ENSBTAG00000018246 | RAB39A, member RAS onco family | [205] |
| *RAB40B* | ENSBTAG00000015387 | RAB40B, member RAS oncogene family | [203] |
| *RAB42* | ENSBTAG00000021525 | RAB42, member RAS oncogene family | [203] |
| *RAB43* | ENSBTAG00000053386 | RAB43, member RAS oncogene family | [204] |
| *RAB7B* | ENSBTAG00000012774 | RAB7B, member RAS oncogene family | [51] |
| *RAB8B* | ENSBTAG00000011146 | RAB8B, member RAS oncogene family | [51] |
| *RABGAP1L* | ENSBTAG00000027809 | RAB GTPase activating protein 1-like | [203] |
| *RABL3* | ENSBTAG00000015898 | RAB, member of RAS oncogene family like 3 | [140] |
| *RAC2* | ENSBTAG00000011043 | Rac family small GTPase 2 | [51] |
| *RAD1* | ENSBTAG00000019209 | RAD1 checkpoint DNA exonuclease | [203] |
| *RAD51AP1* | ENSBTAG00000040065 | RAD51 associated protein 1 | [139] |
| *RAD51L3* | ENSBTAG00000019082 | RAD51 paralog D | [138] |
| *RAE1* | ENSBTAG00000010934 | Ribonucleic acid export 1 | [140] |
| *RALB* | ENSBTAG00000032021 | RAS like proto-onco B | [203] |
| *RALGAPA1* | ENSBTAG00000001282 | Ral GTPase activating protein catalytic subunit alpha 1 | [203] |
| *RAMP1* | ENSBTAG00000011534 | Receptor activity modifying protein 1 | [203] |
| *RANBP2* | ENSBTAG00000000152 | RAN binding protein 2 | [140] |
| *RAP1GDS1* | ENSBTAG00000007522 | Rap1 GTPase-GDP dissociation stimulator 1 | [204] |
| *RAPGEF3* | ENSBTAG00000006828 | Rap guanine nucleotide exchange factor 3 | [203] |
| *RARB* | ENSBTAG00000011518 | Retinoic acid receptor beta | [139] |
| *RARRES2* | ENSBTAG00000004215 | Retinoic acid receptor responder 2 | [138] |
| *RASA2* | ENSBTAG00000015413 | RAS p21 protein activator 2 | [140] |
| *RASA3* | ENSBTAG00000019967 | RAS p21 protein activator 3 | [203] |
| *RASGEF1B* | ENSBTAG00000006561 | RasGEF domain family member 1B | [138] |
| *RASGRP3* | ENSBTAG00000008006 | RAS guanyl releasing protein 3 | [203] |
| *RASL11B* | ENSBTAG00000020647 | RAS like family 11 member B | [51] |
| *RASSF1* | ENSBTAG00000020963 | Ras association domain family member 1 | [140] |
| *RASSF4* | ENSBTAG00000002669 | Ras association domain family member 4 | [138] |
| *RASSF8* | ENSBTAG00000021980 | Ras association domain family member 8 | [203] |
| *RB1* | ENSBTAG00000006640 | RB transcriptional corepressor 1 | [140] |
| *RBBP6* | ENSBTAG00000009441 | RB binding protein 6, ubiquitin ligase | [140] |
| *RBCK1* | ENSBTAG00000017002 | RANBP2-type and C3HC4-type zinc finger containing 1 | [139] |
| *RBL1* | ENSBTAG00000011541 | RB transcriptional corepressor like 1 | [139] |
| *RBM43* | ENSBTAG00000016217 | RNA binding motif protein 43 | [139] |
| *RBP1* | ENSBTAG00000020028 | Retinol binding protein 1 | [51] |
| *RBP4* | ENSBTAG00000000442 | Retinol binding protein 4 | [52] |
| *RBPMS* | ENSBTAG00000033727 | RNA binding protein, mRNA processing factor | [140] |
| *RCBTB1* | ENSBTAG00000034269 | RCC1 and BTB domain containing protein 1 | [203] |
| *RCC1* | ENSBTAG00000003788 | Regulator of chromosome condensation 1 | [203] |
| *RCHY1* | ENSBTAG00000007189 | Ring finger and CHY zinc finger domain containing 1 | [140] |
| *RCL1* | ENSBTAG00000018667 | RNA terminal phosphate cyclase like 1 | [204] |
| *RECQL5* | ENSBTAG00000011715 | RecQ like helicase 5 | [204] |
| *REEP6* | ENSBTAG00000002311 | Receptor accessory protein 6 | [203] |
| *REG3G* | ENSBTAG00000007135 | Regenerating islet-derived 3 gamma | [203] |
| *RELA* | ENSBTAG00000013895 | RELA proto-oncogene, NF-kB subunit | [203] |
| *RELB* | ENSBTAG00000038428 | RELB proto-oncogene, NF-kB subunit | [203] |
| *RELL2* | ENSBTAG00000017371 | RELT like 2 | [203] |
| *RERG* | ENSBTAG00000002174 | RAS like estrogen regulated growth inhibitor | [203] |
| *RESTB* | ENSBTAG00000011789 | RE1 silencing transcription factor B | [203] |
| *REXO5* | ENSBTAG00000006449 | RNA exonuclease 5 | [203] |
| *RFC4* | ENSBTAG00000014727 | Replication factor C subunit 4 | [139] |
| *RGL1* | ENSBTAG00000019630 | Ral guanine nucleotide dissociation stimulator like 1 | [203] |
| *RGS16* | ENSBTAG00000004305 | Regulator of G protein signaling 16 | [139] |
| *RGS2* | ENSBTAG00000020620 | Regulator of G-protein signaling 2, 24kDa | [139] |
| *RGS6* | ENSBTAG00000014189 | Regulator of G protein signaling 6 | [203] |
| *RHAG* | ENSBTAG00000011300 | Rh associated glycoprotein | [203] |
| *RHBDD1* | ENSBTAG00000023963 | Rhomboid domain containing 1 | [203] |
| *RHOBTB2* | ENSBTAG00000035958 | Rho-related BTB domain containing 2 | [203] |
| *RHOF* | ENSBTAG00000032534 | Ras homolog family member F, filopodia associated | [138] |
| *RHOG* | ENSBTAG00000000347 | Ras homolog family member G | [51] |
| *RHOH* | ENSBTAG00000013451 | Ras homolog family member H | [203] |
| *RHOJ* | ENSBTAG00000017200 | Ras homolog family member J | [139] |
| *RIDA* | ENSBTAG00000012595 | Reactive intermediate imine deaminase A homolog | [203] |
| *RILPL2* | ENSBTAG00000007804 | Rab interacting lysosomal protein like 2 | [51] |
| *RING1* | ENSBTAG00000018884 | Ring finger protein 1 | [203] |
| *RIOK2* | ENSBTAG00000011763 | RIO kinase 2 | [139] |
| *RIOK3* | ENSBTAG00000010042 | RIO kinase 3 | [140] |
| *RIOX1* | ENSBTAG00000025372 | Ribosomal oxygenase 1 | [203] |
| *RIPK2* | ENSBTAG00000015271 | Receptor interacting serine/threonine kinase 2 | [140] |
| *RIPOR1* | ENSBTAG00000008860 | RHO family interacting cell polarization regulator 1 | [203] |
| *RIT1* | ENSBTAG00000000105 | Ras like without CAAX 1 | [139] |
| *RLF* | ENSBTAG00000019695 | RLF zinc finger | [139] |
| *RMDN1* | ENSBTAG00000015734 | Regulator of microtubule dynamics 1 | [203] |
| *RMND5A* | ENSBTAG00000021140 | Required for meiotic nuclear division 5 homolog A | [138] |
| *RNASE1* | ENSBTAG00000008793 | Ribonuclease, RNase A family, 1 (pancreatic) | [138] |
| *RNASEH2A* | ENSBTAG00000009661 | Ribonuclease H2 subunit A | [203] |
| *RNASEH2B* | ENSBTAG00000020149 | Ribonuclease H2 subunit B | [203] |
| *RND1* | ENSBTAG00000018773 | Rho family GTPase 1 | [51] |
| *RNF114* | ENSBTAG00000027317 | Ring finger protein 114 | [139] |
| *RNF125* | ENSBTAG00000055051 | Ring finger protein 125 | [203] |
| *RNF157* | ENSBTAG00000016240 | Ring finger protein 157 | [204] |
| *RNF169* | ENSBTAG00000000727 | Ring finger protein 169 | [203] |
| *RNF181* | ENSBTAG00000018286 | Ring finger protein 181 | [138] |
| *RNF19B* | ENSBTAG00000008330 | Ring finger protein 19B | [138] |
| *RNF213* | ENSBTAG00000022489 | E3 ubiquitin-protein ligase RNF213 | [139] |
| *RNF31* | ENSBTAG00000005815 | Ring finger protein 31 | [203] |
| *RNF4* | ENSBTAG00000027453 | Ring finger protein 4 | [203] |
| *RNF41* | ENSBTAG00000026754 | Ring finger protein 41 | [139] |
| *RNMT* | ENSBTAG00000009142 | RNA guanine-7 methyltransferase | [139] |
| *ROBO1* | ENSBTAG00000009851 | Roundabout guidance receptor 1 | [139] |
| *ROGDI* | ENSBTAG00000016331 | Rogdi atypical leucine zipper | [138] |
| *ROPN1L* | ENSBTAG00000003539 | Rhophilin associated tail protein 1 like | [203] |
| *RPAP3* | ENSBTAG00000018690 | RNA polymerase II associated protein 3 | [139] |
| *RPL27A* | ENSBTAG00000005349 | Ribosomal protein L27a | [140] |
| *RPL37* | ENSBTAG00000005142 | Ribosomal protein L37 | [203] |
| *RPRM* | ENSBTAG00000021526 | Reprimo, TP53 dependent G2 arrest mediator homolog | [203] |
| *RPS27L* | ENSBTAG00000012898 | 40S ribosomal protein S27-like | [204] |
| *RPS6KA1* | ENSBTAG00000014447 | Ribosomal protein S6 kinase A1 | [51] |
| *RPS6KB1* | ENSBTAG00000016851 | Ribosomal protein S6 kinase B1 | [203] |
| *RPS6KL1* | ENSBTAG00000006688 | Ribosomal protein S6 kinase like 1 | [203] |
| *RRAD* | ENSBTAG00000013929 | RRAD, Ras related glycolysis inhibitor and calcium channel regulator | [203] |
| *RRAGA* | ENSBTAG00000033543 | Ras related GTP binding A | [140] |
| *RRM2* | ENSBTAG00000008216 | Ribonucleotide reductase regulatory subunit M2 | [139] |
| *RSAD2* | ENSBTAG00000016061 | Radical S-adenosyl methionine domain containing 2 | [139] |
| *RSBN1* | ENSBTAG00000031940 | Round spermatid basic protein 1 | [139] |
| *RSL1D1* | ENSBTAG00000014588 | Ribosomal L1 domain containing 1 | [138] |
| *RTCB* | ENSBTAG00000011070 | RNA 2',3'-cyclic phosphate and 5'-OH ligase | [203] |
| *RTKN2* | ENSBTAG00000014020 | Rhotekin 2 | [203] |
| *RTP3* | ENSBTAG00000018767 | Receptor transporter protein 3 | [139] |
| *RTP4* | ENSBTAG00000032265 | Receptor transporter protein 4 | [139] |
| *RUBCNL* | ENSBTAG00000013371 | Rubicon like autophagy enhancer | [203] |
| *RXFP3* | ENSBTAG00000039929 | Relaxin family peptide receptor 3 | [203] |
| *RYK* | ENSBTAG00000016999 | Receptor like tyrosine kinase | [140] |
| *RYR1* | ENSBTAG00000006999 | Ryanodine receptor 1 | [203] |
| *S100A11* | ENSBTAG00000015145 | S100 calcium binding protein A11 | [138] |
| *S100A12* | ENSBTAG00000012638 | S100 calcium binding protein A12 | [138] |
| *S100A2* | ENSBTAG00000037651 | S100 calcium binding protein A2 | [51] |
| *S100A8* | ENSBTAG00000012640 | S100 calcium binding protein A8 | [138] |
| *S100A9* | ENSBTAG00000006505 | S100 calcium binding protein A9 | [138] |
| *S100G* | ENSBTAG00000017020 | S100 calcium binding protein G | [203] |
| *S100Z* | ENSBTAG00000020201 | S100 calcium binding protein Z | [203] |
| *SAA2* | ENSBTAG00000022394 | Serum amyloid A2 | [203] |
| *SAA3* | ENSBTAG00000049589 | Serum amyloid A3 | [51] |
| *SALL1* | ENSBTAG00000008544 | Spalt like transcription factor 1 | [203] |
| *SALL2* | ENSBTAG00000013249 | Spalt like transcription factor 2 | [204] |
| *SALL4* | ENSBTAG00000003101 | Spalt like transcription factor 4 | [203] |
| *SAMD10* | ENSBTAG00000011655 | Sterile alpha motif domain containing 10 | [203] |
| *SAMD3* | ENSBTAG00000021361 | Sterile alpha motif domain containing 3 | [203] |
| *SAMD9* | ENSBTAG00000053050 | Sterile alpha motif domain containing 9 | [51] |
| *SAMHD1* | ENSBTAG00000022007 | SAM and HD domain containing deoxynucleoside triphosphate triphosphohydrolase 1 | [138] |
| *SAMSN1* | ENSBTAG00000002623 | SAM domain, SH3 domain and nuclear localization signals 1 | [51] |
| *SAP18* | ENSBTAG00000018631 | Sin3A associated protein 18 | [140] |
| *SART3* | ENSBTAG00000031846 | Spliceosome associated factor 3, U4/U6 recycling protein | [140] |
| *SASS6* | ENSBTAG00000015778 | SAS-6 centriolar assembly protein | [139] |
| *SAT1* | ENSBTAG00000017363 | Spermidine/spermine N1-acetyltransferase 1 | [138] |
| *SBK2* | ENSBTAG00000006121 | SH3 domain binding kinase family member 2 | [203] |
| *SBNO2* | ENSBTAG00000020780 | Strawberry notch homolog 2 | [51] |
| *SBSN* | ENSBTAG00000015915 | Suprabasin | [203] |
| *SCAF11* | ENSBTAG00000001810 | SR-related CTD associated factor 11 | [203] |
| *SCCPDH* | ENSBTAG00000003804 | Saccharopine dehydrogenase (putative) | [140] |
| *SCD* | ENSBTAG00000055207 | Stearoyl-CoA desaturase | [138] |
| *SCD5* | ENSBTAG00000022449 | Stearoyl-CoA desaturase 5 | [139] |
| *SCEL* | ENSBTAG00000032821 | Sciellin | [203] |
| *SCGB1A1* | ENSBTAG00000032918 | Secretoglobin family 1A member 1 | [203] |
| *SCIN* | ENSBTAG00000009786 | Scinderin | [203] |
| *SCLY* | ENSBTAG00000017826 | Selenocysteine lyase | [139] |
| *SCMH1* | ENSBTAG00000001629 | Scm polycomb group protein homolog 1 | [203] |
| *SDC2* | ENSBTAG00000014357 | Syndecan 2 | [51] |
| *SDC4* | ENSBTAG00000015127 | Syndecan 4 | [138] |
| *SDCBP* | ENSBTAG00000019910 | Syndecan binding protein | [138] |
| *SDF2L1* | ENSBTAG00000000067 | Stromal cell derived factor 2 like 1 | [138] |
| *SDHAF2* | ENSBTAG00000015266 | Succinate dehydrogenase complex assembly factor 2 | [138] |
| *SDR39U1* | ENSBTAG00000004788 | Short chain dehydrogenase/reductase family 39U member 1 | [204] |
| *SDS* | ENSBTAG00000031814 | Serine dehydratase | [139] |
| *SEC16B* | ENSBTAG00000000854 | SEC16 homolog B, endoplasmic reticulum export factor | [51] |
| *SEC24D* | ENSBTAG00000003619 | SEC24 homolog D, COPII coat complex component | [204] |
| *SEL1L* | ENSBTAG00000008083 | SEL1L adaptor subunit of ERAD E3 ubiquitin ligase | [203] |
| *SELENBP1* | ENSBTAG00000008091 | Selenium binding protein 1 | [138] |
| *SELENOK* | ENSBTAG00000032374 | Selenoprotein K | [203] |
| *SELENOP* | ENSBTAG00000054085 | Selenoprotein P | [203] |
| *SELL* | ENSBTAG00000011515 | Selectin L | [51] |
| *SELP* | ENSBTAG00000020755 | Selectin P | [52] |
| *SEMA4D* | ENSBTAG00000001941 | Semaphorin 4D | [51] |
| *SEMA6A* | ENSBTAG00000020489 | Semaphorin 6A | [51] |
| *SENP2* | ENSBTAG00000001749 | SUMO specific peptidase 2 | [138] |
| *SERINC2* | ENSBTAG00000035081 | Serine incorporator 2 | [203] |
| *SERINC4* | ENSBTAG00000017211 | Serine incorporator 4 | [140] |
| *SERPINA1* | ENSBTAG00000018843 | Serpin family A member 1 | [52] |
| *SERPINA3-1* | ENSBTAG00000046540 | Serpin peptidase inhibitor, clade A (alpha-1 antiproteinase, antitrypsin), member 3 | [51] |
| *SERPINA3-7* | ENSBTAG00000048094 | Endopin 2 | [203] |
| *SERPINB1* | ENSBTAG00000011975 | Serpin family B member 1 | [140] |
| *SERPINB2* | ENSBTAG00000023198 | Serpin family B member 2 | [139] |
| *SERPINB9* | ENSBTAG00000031267 | Serpin family B member 9 | [203] |
| *SERPINE3* | ENSBTAG00000005284 | Serpin family E member 3 | [203] |
| *SERPINF1* | ENSBTAG00000009705 | Serpin family F member 1 | [52] |
| *SERPINF2* | ENSBTAG00000020859 | Serpin family F member 2 | [52] |
| *SERPING1* | ENSBTAG00000016267 | Serpin family G member 1 | [138] |
| *SERTAD4* | ENSBTAG00000010850 | SERTA domain containing 4 | [203] |
| *SESN3* | ENSBTAG00000004034 | Sestrin 3 | [140] |
| *SETD7* | ENSBTAG00000003581 | SET domain containing 7, histone lysine methyltransferase | [204] |
| *SETDB2* | ENSBTAG00000007329 | SET domain bifurcated histone lysine methyltransferase 2 | [203] |
| *SETMAR* | ENSBTAG00000018935 | SET domain without mariner transposase fusion | [203] |
| *SEZ6L* | ENSBTAG00000006569 | Seizure related 6 homolog like | [203] |
| *SEZ6L2* | ENSBTAG00000007955 | Seizure related 6 homolog like 2 | [204] |
| *SF3A2* | ENSBTAG00000025452 | Splicing factor 3a subunit 2 | [140] |
| *SFPQ* | ENSBTAG00000016328 | Splicing factor proline and glutamine rich | [140] |
| *SFRP2* | ENSBTAG00000018563 | Secreted frizzled related protein 2 | [204] |
| *SFTPC* | ENSBTAG00000012560 | Surfactant protein C | [203] |
| *SFXN4* | ENSBTAG00000008730 | Sideroflexin 4 | [203] |
| *SGIP1* | ENSBTAG00000011091 | SH3GL interacting endocytic adaptor 1 | [203] |
| *SGMS2* | ENSBTAG00000016805 | Sphingomyelin synthase 2 | [140] |
| *SH2D2A* | ENSBTAG00000024470 | SH2 domain containing 2A | [203] |
| *SH2D3A* | ENSBTAG00000013857 | SH2 domain containing 3A | [203] |
| *SH3BGRL2* | ENSBTAG00000055014 | SH3 domain binding glutamate rich protein like 2 | [138] |
| *SH3BP4* | ENSBTAG00000013869 | SH3 domain binding protein 4 | [138] |
| *SH3GL2* | ENSBTAG00000014103 | SH3 domain containing GRB2 like 2, endophilin A1 | [138] |
| *SH3TC2* | ENSBTAG00000017151 | SH3 domain and tetratricopeptide repeats 2 | [51] |
| *SH3YL1* | ENSBTAG00000020495 | SH3 and SYLF domain containing 1 | [138] |
| *SHANK1* | ENSBTAG00000013107 | SH3 and multiple ankyrin repeat domains 1 | [203] |
| *SHD* | ENSBTAG00000039696 | Src homology 2 domain containing transforming protein D | [203] |
| *SHISAL1* | ENSBTAG00000003574 | Shisa like 1 | [203] |
| *SHISAL2B* | ENSBTAG00000009468 | Shisa like 2B | [203] |
| *SHOC2* | ENSBTAG00000007709 | SHOC2 leucine rich repeat scaffold protein | [138] |
| *SHPK* | ENSBTAG00000000829 | Sedoheptulokinase | [204] |
| *SHQ1* | ENSBTAG00000005436 | SHQ1, H/ACA ribonucleoprotein assembly factor | [140] |
| *SIAH1* | ENSBTAG00000010868 | Siah E3 ubiquitin protein ligase 1 | [203] |
| *SIGLEC1* | ENSBTAG00000013167 | Sialic acid binding Ig like lectin 1 | [138] |
| *SIK1* | ENSBTAG00000011437 | Salt inducible kinase 1 | [139] |
| *SIRPA* | ENSBTAG00000007213 | Signal regulatory protein alpha | [51] |
| *SIRPD* | ENSBTAG00000033397 | Signal-regulatory protein delta | [203] |
| *SIRT6* | ENSBTAG00000019909 | Sirtuin 6 | [203] |
| *SIX1* | ENSBTAG00000012139 | SIX homeobox 1 | [203] |
| *SIX2* | ENSBTAG00000004159 | SIX homeobox 2 | [203] |
| *SKA1* | ENSBTAG00000018216 | Spindle and kinetochore associated complex subunit 1 | [139] |
| *SKA3* | ENSBTAG00000003314 | Spindle and kinetochore associated complex subunit 3 | [139] |
| *SKAP2* | ENSBTAG00000005650 | Src kinase associated phosphoprotein 2 | [51] |
| *SLA* | ENSBTAG00000007828 | Src like adaptor | [51] |
| *SLAMF7* | ENSBTAG00000001197 | SLAM family member 7 | [138] |
| *SLAMF8* | ENSBTAG00000016698 | SLAM family member 8 | [139] |
| *SLC10A4* | ENSBTAG00000004888 | Solute carrier family 10 member 4 | [203] |
| *SLC12A2* | ENSBTAG00000009637 | Solute carrier family 12 member 2 | [203] |
| *SLC16A13* | ENSBTAG00000020853 | Solute carrier family 16 member 13 | [203] |
| *SLC18A2* | ENSBTAG00000004739 | Solute carrier family 18 member A2 | [204] |
| *SLC18A3* | ENSBTAG00000014990 | Solute carrier family 18 member A3 | [203] |
| *SLC1A1* | ENSBTAG00000019125 | Solute carrier family 1 member 1 | [139] |
| *SLC1A7* | ENSBTAG00000012516 | Solute carrier family 1 member 7 | [203] |
| *SLC22A15* | ENSBTAG00000000664 | Solute carrier family 22 member 15 | [140] |
| *SLC22A18* | ENSBTAG00000012742 | Solute carrier family 22 member 18 | [203] |
| *SLC22A2* | ENSBTAG00000009583 | Solute carrier family 22 member 2 | [203] |
| *SLC22A20P* | ENSBTAG00000010233 | Solute carrier family 22 member 20, pseudogene | [203] |
| *SLC25A19* | ENSBTAG00000000202 | Solute carrier family 25 member 19 | [51] |
| *SLC25A21* | ENSBTAG00000019350 | Solute carrier family 25 member 21 | [139] |
| *SLC25A28* | ENSBTAG00000012107 | Solute carrier family 25 member 28 | [139] |
| *SLC25A44* | ENSBTAG00000009431 | Solute carrier family 25 member 44 | [203] |
| *SLC26A7* | ENSBTAG00000032301 | Solute carrier family 26 member 7 | [203] |
| *SLC27A2* | ENSBTAG00000004303 | Solute carrier family 27 member 2 | [138] |
| *SLC27A5* | ENSBTAG00000015164 | Solute carrier family 27 member 5 | [138] |
| *SLC29A1* | ENSBTAG00000015131 | Solute carrier family 29 member 1 | [138] |
| *SLC2A1* | ENSBTAG00000009617 | Solute carrier family 2 member 1 | [139] |
| *SLC2A10* | ENSBTAG00000002942 | Solute carrier family 2 member 10 | [203] |
| *SLC2A3* | ENSBTAG00000004556 | Solute carrier family 2 member 3 | [139] |
| *SLC2A4* | ENSBTAG00000009190 | Solute carrier family 2 member 4 | [203] |
| *SLC2A6* | ENSBTAG00000001824 | Solute carrier family 2 member 6 | [139] |
| *SLC30A10* | ENSBTAG00000004414 | Solute carrier family 30 member 10 | [203] |
| *SLC30A5* | ENSBTAG00000001013 | Solute carrier family 30 member 5 | [203] |
| *SLC31A2* | ENSBTAG00000005115 | Solute carrier family 31 member 2 | [138] |
| *SLC35F6* | ENSBTAG00000018761 | Solute carrier family 35 member F6 | [203] |
| *SLC36A2* | ENSBTAG00000020809 | Solute carrier family 36 member 2 | [203] |
| *SLC37A2* | ENSBTAG00000016704 | Solute carrier family 37 member 2 | [203] |
| *SLC38A10* | ENSBTAG00000018271 | Solute carrier family 38 member 10 | [203] |
| *SLC38A7* | ENSBTAG00000007170 | Solute carrier family 38 member 7 | [138] |
| *SLC39A10* | ENSBTAG00000016782 | Solute carrier family 39 member 10 | [203] |
| *SLC39A2* | ENSBTAG00000000936 | Solute carrier family 39 member 2 | [51] |
| *SLC39A8* | ENSBTAG00000005668 | Solute carrier family 39 member 8 | [203] |
| *SLC3A2* | ENSBTAG00000013240 | Solute carrier family 3 member 2 | [204] |
| *SLC40A1* | ENSBTAG00000010498 | Solute carrier family 40 member 1 | [139] |
| *SLC46A1* | ENSBTAG00000002817 | Solute carrier family 46 member 1 | [203] |
| *SLC46A2* | ENSBTAG00000013670 | Solute carrier family 46 member 2 | [203] |
| *SLC4A1* | ENSBTAG00000001610 | Solute carrier family 4 member 1 | [203] |
| *SLC5A5* | ENSBTAG00000015830 | Solute carrier family 5 member 5 | [51] |
| *SLC6A11* | ENSBTAG00000038962 | Solute carrier family 6 member 11 | [203] |
| *SLC6A14* | ENSBTAG00000000185 | Solute carrier family 6 member 14 | [51] |
| *SLC6A7* | ENSBTAG00000000910 | Solute carrier family 6 member 7 | [138] |
| *SLC7A5* | ENSBTAG00000006731 | Solute carrier family 7 member 5 | [51] |
| *SLC7A7* | ENSBTAG00000014821 | Solute carrier family 7 member 7 | [51] |
| *SLC7A8* | ENSBTAG00000007415 | Solute carrier family 7 member 8 | [204] |
| *SLC8A1* | ENSBTAG00000013861 | Solute carrier family 8 member A1 | [203] |
| *SLC8A3* | ENSBTAG00000027080 | Solute carrier family 8 member A3 | [203] |
| *SLC9A7* | ENSBTAG00000003290 | Solute carrier family 9 member A7 | [139] |
| *SLCO1A2* | ENSBTAG00000003282 | Solute carrier organic anion transporter family member 1A2 | [203] |
| *SLCO3A1* | ENSBTAG00000001652 | Solute carrier organic anion transporter family member 3A1 | [139] |
| *SLCO4A1* | ENSBTAG00000016388 | Solute carrier organic anion transporter family member 4A1 | [140] |
| *SLCO4C1* | ENSBTAG00000016359 | Solute carrier organic anion transporter family member 4C1 | [205] |
| *SLCO6A1* | ENSBTAG00000030210 | Solute carrier organic anion transporter family member 6A1 | [205] |
| *SLIRP* | ENSBTAG00000008135 | SRA stem-loop interacting RNA binding protein | [203] |
| *SLPI* | ENSBTAG00000004148 | Secretory leukocyte peptidase inhibitor | [139] |
| *SLX4* | ENSBTAG00000004509 | SLX4 structure-specific endonuclease subunit | [203] |
| *SMAD2* | ENSBTAG00000039916 | SMAD family member 2 | [140] |
| *SMAD7* | ENSBTAG00000012441 | SMAD family member 7 | [139] |
| *SMARCD1* | ENSBTAG00000037935 | SWI/SNF related, matrix associated, actin dependent regulator of chromatin, subfamily d, member 1 | [203] |
| *SMC1A* | ENSBTAG00000017761 | Structural maintenance of chromosomes 1A | [203] |
| *SMC5* | ENSBTAG00000018437 | Structural maintenance of chromosomes 5 | [203] |
| *SMIM26* | ENSBTAG00000051249 | Small integral membrane protein 26 | [203] |
| *SMIM3* | ENSBTAG00000010487 | Small integral membrane protein 3 | [203] |
| *SMPDL3B* | ENSBTAG00000012997 | Sphingomyelin phosphodiesterase acid like 3B | [139] |
| *SNAP23* | ENSBTAG00000005661 | Synaptosome associated protein 23 | [139] |
| *SNAPC1* | ENSBTAG00000020937 | Small nuclear RNA activating complex polypeptide 1 | [139] |
| *SNAPIN* | ENSBTAG00000012470 | SNAP associated protein | [140] |
| *SNN* | ENSBTAG00000054404 | Stannin | [51] |
| *SNORA8* | ENSBTAG00000042369 | Small nucleolar RNA SNORA8 | [204] |
| *SNORD65* | ENSBTAG00000047641 | Small nucleolar RNA SNORD65 | [204] |
| *SNRNP27* | ENSBTAG00000037746 | Small nuclear ribonucleoprotein U4/U6.U5 subunit 27 | [140] |
| *SNRPG* | ENSBTAG00000053210 | Small nuclear ribonucleoprotein polypeptide G | [204] |
| *SNTA1* | ENSBTAG00000000512 | Syntrophin alpha 1 | [204] |
| *SNW1* | ENSBTAG00000008136 | SNW domain containing 1 | [203] |
| *SNX13* | ENSBTAG00000014074 | Sorting nexin 13 | [140] |
| *SNX22* | ENSBTAG00000016821 | Sorting nexin 22 | [140] |
| *SOAT1* | ENSBTAG00000004059 | Sterol O-acyltransferase 1 | [51] |
| *SOCS3* | ENSBTAG00000008441 | Suppressor of cytokine signaling 3 | [51] |
| *SOD2* | ENSBTAG00000006523 | Superoxide dismutase 2 | [138] |
| *SOX13* | ENSBTAG00000008132 | SRY-box transcription factor 13 | [203] |
| *SOX6* | ENSBTAG00000044185 | SRY-box transcription factor 6 | [203] |
| *SP1* | ENSBTAG00000003021 | Sp1 transcription factor | [203] |
| *SP110* | ENSBTAG00000015752 | SP110 nuclear body protein | [140] |
| *SP6* | ENSBTAG00000001825 | Sp6 transcription factor | [203] |
| *SPACA9* | ENSBTAG00000005189 | Sperm acrosome associated 9 | [203] |
| *SPAG5* | ENSBTAG00000013100 | Sperm associated antigen 5 | [139] |
| *SPAG6* | ENSBTAG00000033291 | Sperm associated antigen 6 | [203] |
| *SPARC* | ENSBTAG00000014835 | Secreted protein acidic and cysteine rich | [138] |
| *SPARCL1* | ENSBTAG00000004094 | SPARC like 1 | [203] |
| *SPART* | ENSBTAG00000008040 | Spartin | [203] |
| *SPDEF* | ENSBTAG00000021993 | SAM pointed domain containing ETS transcription factor | [203] |
| *SPDL1* | ENSBTAG00000008180 | Spindle apparatus coiled-coil protein 1 | [203] |
| *SPECC1* | ENSBTAG00000000928 | Sperm antigen with calponin homology and coiled-coil domains 1 | [140] |
| *SPEF1* | ENSBTAG00000006578 | Sperm flagellar 1 | [140] |
| *SPEG* | ENSBTAG00000046176 | Striated muscle enriched protein kinase | [203] |
| *SPG11* | ENSBTAG00000000362 | SPG11 vesicle trafficking associated, spatacsin | [138] |
| *SPON1* | ENSBTAG00000009150 | Spondin 1 | [203] |
| *SPP2* | ENSBTAG00000002030 | Secreted phosphoprotein 2 | [203] |
| *SPRY1* | ENSBTAG00000021245 | Sprouty RTK signaling antagonist 1 | [139] |
| *SPRYD7* | ENSBTAG00000018829 | SPRY domain containing 7 | [203] |
| *SPTAN1* | ENSBTAG00000015327 | Spectrin alpha, non-erythrocytic 1 | [138] |
| *SPTLC2* | ENSBTAG00000020480 | Serine palmitoyltransferase long chain base subunit 2 | [140] |
| *SQRDL* | ENSBTAG00000010365 | Sulfide quinone oxidoreductase | [139] |
| *SQSTM1* | ENSBTAG00000015591 | Sequestosome 1 | [140] |
| *SRGN* | ENSBTAG00000017863 | Serglycin | [138] |
| *SRL* | ENSBTAG00000001078 | Sarcalumenin | [139] |
| *SRPX2* | ENSBTAG00000037686 | Sushi repeat containing protein X-linked 2 | [203] |
| *SRSF3* | ENSBTAG00000040006 | Serine and arginine rich splicing factor 3 | [203] |
| *SRXN1* | ENSBTAG00000010913 | Sulfiredoxin 1 | [139] |
| *SSB* | ENSBTAG00000008716 | Small RNA binding exonuclease protection factor La | [138] |
| *SSPN* | ENSBTAG00000000393 | Sarcospan | [139] |
| *SSR1* | ENSBTAG00000022731 | Signal sequence receptor subunit 1 | [138] |
| *ST6GAL1* | ENSBTAG00000010357 | ST6 beta-galactoside alpha-2,6-sialyltransferase 1 | [138] |
| *STAC2* | ENSBTAG00000008366 | SH3 and cysteine rich domain 2 | [203] |
| *STARD9* | ENSBTAG00000048958 | StAR related lipid transfer domain containing 9 | [203] |
| *STAT1* | ENSBTAG00000007867 | Signal transducer and activator of transcription 1 | [140] |
| *STAT2* | ENSBTAG00000004380 | Signal transducer and activator of transcription 2 | [140] |
| *STAT4* | ENSBTAG00000046699 | Signal transducer and activator of transcription 4 | [203] |
| *STAT6* | ENSBTAG00000006335 | Signal transducer and activator of transcription 6 | [138] |
| *STAU2* | ENSBTAG00000002076 | Staufen double-stranded RNA binding protein 2 | [138] |
| *STC1* | ENSBTAG00000001687 | Stanniocalcin 1 | [204] |
| *STEAP2* | ENSBTAG00000003506 | STEAP2 metalloreductase | [138] |
| *STEEP1* | ENSBTAG00000047169 | STING1 ER exit protein 1 | [203] |
| *STIM2* | ENSBTAG00000019353 | Stromal interaction molecule 2 | [203] |
| *STK17A* | ENSBTAG00000008143 | Serine/threonine kinase 17a | [203] |
| *STK38* | ENSBTAG00000011126 | Serine/threonine kinase 38 | [139] |
| *STK38L* | ENSBTAG00000011798 | Serine/threonine kinase 38 like | [139] |
| *STN1* | ENSBTAG00000015019 | STN1 subunit of CST complex | [203] |
| *STOM* | ENSBTAG00000038375 | Stomatin | [204] |
| *STRAP* | ENSBTAG00000014175 | Serine/threonine kinase receptor associated protein | [138] |
| *STX12* | ENSBTAG00000001192 | Syntaxin 12 | [140] |
| *STX2* | ENSBTAG00000007988 | Syntaxin 2 | [51] |
| *STXBP3* | ENSBTAG00000007330 | Syntaxin binding protein 3 | [139] |
| *SULF1* | ENSBTAG00000004720 | Sulfatase 1 | [139] |
| *SULT1C2* | ENSBTAG00000038369 | Sulfotransferase family, cytosolic, 1C, member 2 | [203] |
| *SULT1E1* | ENSBTAG00000011952 | Sulfotransferase family 1E member 1 | [51] |
| *SULT6B1* | ENSBTAG00000008707 | Sulfotransferase family 6B member 1 | [203] |
| *SUSD3* | ENSBTAG00000000181 | Sushi domain containing 3 | [51] |
| *SUSD6* | ENSBTAG00000003857 | Sushi domain containing 6 | [203] |
| *SVIP* | ENSBTAG00000054222 | Small VCP interacting protein | [205] |
| *SYNDIG1L* | ENSBTAG00000052950 | Synapse differentiation inducing 1 like | [203] |
| *SYNE1* | ENSBTAG00000009362 | Spectrin repeat containing nuclear envelope protein 1 | [138] |
| *SYNE4* | ENSBTAG00000019679 | Spectrin repeat containing nuclear envelope family member 4 | [203] |
| *SYNPO2* | ENSBTAG00000006434 | Synaptopodin 2 | [203] |
| *SYT1* | ENSBTAG00000034693 | Synaptotagmin 1 | [138] |
| *SYTL1* | ENSBTAG00000010253 | Synaptotagmin like 1 | [205] |
| *TAAR6* | ENSBTAG00000052548 | Trace amine associated receptor 6 | [203] |
| *TAB3* | ENSBTAG00000048159 | TGF-beta activated kinase 1 (MAP3K7) binding protein 3 | [203] |
| *TADA3* | ENSBTAG00000007961 | Transcriptional adaptor 3 | [140] |
| *TAF1D* | ENSBTAG00000003545 | TATA-box binding protein associated factor, RNA polymerase I subunit D | [204] |
| *TAF6L* | ENSBTAG00000009484 | TATA-box binding protein associated factor 6 like | [203] |
| *TAF9* | ENSBTAG00000027980 | TATA-box binding protein associated factor 9 | [203] |
| *TAGLN3* | ENSBTAG00000004599 | Transgelin 3 | [138] |
| *TANK* | ENSBTAG00000006654 | TRAF family member associated NFKB activator | [140] |
| *TAP* | ENSBTAG00000048171 | Tracheal antimicrobial peptide | [203] |
| *TAP1* | ENSBTAG00000008953 | Transporter 1, ATP binding cassette subfamily B member | [138] |
| *TAPBPL* | ENSBTAG00000014728 | TAP binding protein like | [139] |
| *TAX1BP1* | ENSBTAG00000019020 | Tax1 binding protein 1 | [139] |
| *TBC1D10C* | ENSBTAG00000013133 | TBC1 domain family member 10C | [203] |
| *TBC1D13* | ENSBTAG00000012480 | TBC1 domain family member 13 | [203] |
| *TBC1D14* | ENSBTAG00000005493 | TBC1 domain family member 14 | [138] |
| *TBC1D5* | ENSBTAG00000030581 | TBC1 domain family member 5 | [203] |
| *TBC1D9B* | ENSBTAG00000015611 | TBC1 domain family member 9B | [140] |
| *TBCEL* | ENSBTAG00000018213 | Tubulin folding cofactor E like | [203] |
| *TCEA2* | ENSBTAG00000021836 | Transcription elongation factor A2 | [204] |
| *TCEAL4* | ENSBTAG00000022204 | Transcription elongation factor A like 4 | [140] |
| *TCF19* | ENSBTAG00000014435 | Transcription factor 19 | [140] |
| *TCF20* | ENSBTAG00000000662 | Transcription factor 20 | [203] |
| *TCIM* | ENSBTAG00000033304 | Transcriptional and immune response regulator | [203] |
| *TCN1* | ENSBTAG00000020580 | Transcobalamin 1 | [203] |
| *TCP11L2* | ENSBTAG00000002127 | T-complex 11 like 2 | [203] |
| *TDH* | ENSBTAG00000000011 | L-threonine dehydrogenase | [203] |
| *TDRD7* | ENSBTAG00000003719 | Tudor domain containing 7 | [139] |
| *TEK* | ENSBTAG00000020148 | TEK receptor tyrosine kinase | [138] |
| *TENM3* | ENSBTAG00000021133 | Teneurin transmembrane protein 3 | [203] |
| *TENT2* | ENSBTAG00000006751 | Terminal nucleotidyltransferase 2 | [203] |
| *TESK1* | ENSBTAG00000011406 | Testis associated actin remodelling kinase 1 | [140] |
| *TEX12* | ENSBTAG00000016548 | Testis expressed 12 | [203] |
| *TEX36* | ENSBTAG00000007011 | Testis expressed 36 | [203] |
| *TFCP2* | ENSBTAG00000019312 | Transcription factor CP2 | [203] |
| *TFCP2L1* | ENSBTAG00000007765 | Transcription factor CP2 like 1 | [203] |
| *TFEB* | ENSBTAG00000012384 | Transcription factor EB | [205] |
| *TFF3* | ENSBTAG00000021276 | Trefoil factor 3 | [52] |
| *TFPI2* | ENSBTAG00000015844 | Tissue factor pathway inhibitor 2 | [139] |
| *TGDS* | ENSBTAG00000005309 | TDP-glucose 4,6-dehydratase | [140] |
| *TGFB1* | ENSBTAG00000020457 | Transforming growth factor beta 1 | [204] |
| *TGFB2* | ENSBTAG00000005359 | Transforming growth factor beta 2 | [52] |
| *TGFB3* | ENSBTAG00000012004 | Transforming growth factor beta 3 | [203] |
| *TGFBI* | ENSBTAG00000009513 | Transforming growth factor beta induced | [203] |
| *TGIF1* | ENSBTAG00000007718 | TGFB induced factor homeobox 1 | [140] |
| *TGM2* | ENSBTAG00000016208 | Transglutaminase 2 | [138] |
| *TGM3* | ENSBTAG00000001785 | Transglutaminase 3 | [51] |
| *THBD* | ENSBTAG00000048591 | Thrombomodulin | [203] |
| *THBS1* | ENSBTAG00000002006 | Thrombospondin 1 | [52] |
| *THOC1* | ENSBTAG00000019215 | THO complex 1 | [140] |
| *THY1* | ENSBTAG00000019627 | Thy-1 cell surface antigen | [204] |
| *TIFA* | ENSBTAG00000049422 | TRAF interacting protein with forkhead associated domain | [139] |
| *TIMP4* | ENSBTAG00000020263 | TIMP metallopeptidase inhibitor 4 | [203] |
| *TIPARP* | ENSBTAG00000012120 | TCDD inducible poly(ADP-ribose) polymerase | [139] |
| *TJP1* | ENSBTAG00000015398 | Tight junction protein 1 | [203] |
| *TKTL2* | ENSBTAG00000008947 | Transketolase like 2 | [203] |
| *TLE4* | ENSBTAG00000003532 | TLE family member 4, transcriptional corepressor | [204] |
| *TLN2* | ENSBTAG00000003667 | Talin 2 | [203] |
| *TLR5* | ENSBTAG00000000477 | Toll like receptor 5 | [203] |
| *TM4SF18* | ENSBTAG00000019960 | Transmembrane 4 L six family member 18 | [203] |
| *TMCC3* | ENSBTAG00000014375 | Transmembrane and coiled-coil domain family 3 | [204] |
| *TMED10* | ENSBTAG00000005694 | Transmembrane p24 trafficking protein 10 | [203] |
| *TMED8* | ENSBTAG00000020469 | Transmembrane p24 trafficking protein family member 8 | [203] |
| *TMEFF2* | ENSBTAG00000014832 | Transmembrane protein with EGF like and two follistatin like domains 2 | [203] |
| *TMEM106A* | ENSBTAG00000006801 | Transmembrane protein 106A | [139] |
| *TMEM106B* | ENSBTAG00000019750 | Transmembrane protein 106B | [204] |
| *TMEM125* | ENSBTAG00000021082 | Transmembrane protein 125 | [140] |
| *TMEM140* | ENSBTAG00000001576 | Transmembrane protein 140 | [139] |
| *TMEM156* | ENSBTAG00000015649 | Transmembrane protein 156 | [51] |
| *TMEM164* | ENSBTAG00000037413 | Transmembrane protein 164 | [204] |
| *TMEM177* | ENSBTAG00000009058 | Transmembrane protein 177 | [203] |
| *TMEM183A* | ENSBTAG00000002874 | Transmembrane protein 183A | [140] |
| *TMEM185B* | ENSBTAG00000026829 | Transmembrane protein 185B | [203] |
| *TMEM200C* | ENSBTAG00000046396 | Transmembrane protein 200C | [140] |
| *TMEM204* | ENSBTAG00000007246 | Transmembrane protein 204 | [139] |
| *TMEM209* | ENSBTAG00000015588 | Transmembrane protein 209 | [138] |
| *TMEM216* | ENSBTAG00000021517 | Transmembrane protein 216 | [204] |
| *TMEM268* | ENSBTAG00000000204 | Transmembrane protein 268 | [203] |
| *TMEM51* | ENSBTAG00000005300 | Transmembrane protein 51 | [138] |
| *TMEM63C* | ENSBTAG00000016283 | Transmembrane protein 63C | [203] |
| *TMEM97* | ENSBTAG00000008109 | Transmembrane protein 97 | [140] |
| *TMOD4* | ENSBTAG00000018071 | Tropomodulin 4 | [203] |
| *TMPRSS2* | ENSBTAG00000009132 | Transmembrane serine protease 2 | [140] |
| *TMTC1* | ENSBTAG00000005370 | Transmembrane O-mannosyltransferase targeting cadherins 1 | [203] |
| *TNFAIP2* | ENSBTAG00000035995 | TNF alpha induced protein 2 | [138] |
| *TNFAIP3* | ENSBTAG00000000436 | TNF alpha induced protein 3 | [139] |
| *TNFRSF11A* | ENSBTAG00000007569 | TNF receptor superfamily member 11a | [203] |
| *TNFRSF1A* | ENSBTAG00000004211 | TNF receptor superfamily member 1A | [51] |
| *TNFRSF1B* | ENSBTAG00000024928 | TNF receptor superfamily member 1B | [138] |
| *TNFRSF6B* | ENSBTAG00000027407 | TNF receptor superfamily member 6b | [51] |
| *TNFSF10* | ENSBTAG00000018994 | TNF superfamily member 10 | [139] |
| *TNFSF13B* | ENSBTAG00000011563 | Tumor necrosis factor ligand superfamily member 13B-like | [139] |
| *TNIP1* | ENSBTAG00000012671 | TNFAIP3 interacting protein 1 | [51] |
| *TNIP2* | ENSBTAG00000015126 | TNFAIP3 interacting protein 2 | [203] |
| *TNNC1* | ENSBTAG00000045757 | Troponin C1, slow skeletal and cardiac type | [203] |
| *TNNC2* | ENSBTAG00000046725 | Troponin C2, fast skeletal type | [203] |
| *TNNI3K* | ENSBTAG00000000965 | TNNI3 interacting kinase | [203] |
| *TNPO3* | ENSBTAG00000004992 | Transportin 3 | [140] |
| *TNRC6A* | ENSBTAG00000017999 | Trinucleotide repeat containing adaptor 6A | [203] |
| *TNRC6B* | ENSBTAG00000021076 | Trinucleotide repeat containing adaptor 6B | [203] |
| *TOE1* | ENSBTAG00000011243 | Target of EGR1, exonuclease | [140] |
| *TOM1L1* | ENSBTAG00000007013 | Target of myb1 like 1 membrane trafficking protein | [203] |
| *TOP1* | ENSBTAG00000007960 | DNA topoisomerase I | [140] |
| *TOR1AIP2* | ENSBTAG00000035230 | Torsin 1A interacting protein 2 | [139] |
| *TOR3A* | ENSBTAG00000014006 | Torsin family 3 member A | [139] |
| *TOR4A* | ENSBTAG00000023787 | Torsin family 4 member A | [203] |
| *TP53* | ENSBTAG00000001069 | Tumor protein p53 | [140] |
| *TP53INP2* | ENSBTAG00000005504 | Tumor protein p53 inducible nuclear protein 2 | [139] |
| *TPI1* | ENSBTAG00000019782 | Triosephosphate isomerase 1 | [138] |
| *TPM2* | ENSBTAG00000011424 | Tropomyosin 2 | [204] |
| *TPM3* | ENSBTAG00000033217 | Tropomyosin 3 | [138] |
| *TPMT* | ENSBTAG00000019300 | Thiopurine S-methyltransferase | [138] |
| *TPPP3* | ENSBTAG00000019822 | Tubulin polymerization promoting protein family member 3 | [138] |
| *TPRG1* | ENSBTAG00000032538 | Tumor protein p63 regulated 1 | [203] |
| *TRADD* | ENSBTAG00000012642 | TNFRSF1A associated via death domain | [140] |
| *TRAF3IP3* | ENSBTAG00000002846 | TRAF3 interacting protein 3 | [203] |
| *TREM1* | ENSBTAG00000017593 | Triggering receptor expressed on myeloid cells 1 | [51] |
| *TREM2* | ENSBTAG00000007275 | Triggering receptor expressed on myeloid cells 2 | [205] |
| *TREML2* | ENSBTAG00000015707 | Triggering receptor expressed on myeloid cells like 2 | [51] |
| *TRIB1* | ENSBTAG00000023179 | Tribbles pseudokinase 1 | [138] |
| *TRIB3* | ENSBTAG00000017007 | Tribbles pseudokinase 3 | [204] |
| *TRIM13* | ENSBTAG00000008173 | Tripartite motif containing 13 | [203] |
| *TRIM21* | ENSBTAG00000013900 | Tripartite motif containing 21 | [140] |
| *TRIM38* | ENSBTAG00000018523 | Tripartite motif containing 38 | [138] |
| *TRIM5* | ENSBTAG00000052606 | Tripartite motif containing 5 | [139] |
| *TRIM63* | ENSBTAG00000005085 | Tripartite motif containing 63 | [203] |
| *TRIP11* | ENSBTAG00000026953 | Thyroid hormone receptor interactor 11 | [139] |
| *TRIP12* | ENSBTAG00000021653 | Thyroid hormone receptor interactor 12 | [140] |
| *TRMT13* | ENSBTAG00000015781 | tRNA methyltransferase 13 homolog | [203] |
| *TRMT61A* | ENSBTAG00000031327 | TRNA methyltransferase 61A | [203] |
| *TRPC1* | ENSBTAG00000002232 | Transient receptor potential cation channel subfamily C member 1 | [139] |
| *TRPV2* | ENSBTAG00000003014 | Transient receptor potential cation channel subfamily V member 2 | [203] |
| *TSC22D3* | ENSBTAG00000045877 | TSC22 domain family member 3 | [204] |
| *TSHZ2* | ENSBTAG00000007917 | Teashirt zinc finger homeobox 2 | [140] |
| *TSN* | ENSBTAG00000006059 | Translin | [203] |
| *TSPAN2* | ENSBTAG00000015961 | Tetraspanin 2 | [139] |
| *TSPO* | ENSBTAG00000018073 | Translocator protein | [51] |
| *TSPYL1* | ENSBTAG00000010885 | TSPY like 1 | [140] |
| *TTC12* | ENSBTAG00000010008 | Tetratricopeptide repeat domain 12 | [203] |
| *TTC14* | ENSBTAG00000001968 | Tetratricopeptide repeat domain 14 | [203] |
| *TTC4* | ENSBTAG00000015931 | Tetratricopeptide repeat domain 4 | [140] |
| *TTC6* | ENSBTAG00000015095 | Tetratricopeptide repeat domain 6 | [203] |
| *TTC9B* | ENSBTAG00000046666 | Tetratricopeptide repeat domain 9B | [204] |
| *TTC9C* | ENSBTAG00000000773 | Tetratricopeptide repeat domain 9C | [138] |
| *TTLL1* | ENSBTAG00000012030 | Tubulin tyrosine ligase like 1 | [138] |
| *TTPAL* | ENSBTAG00000018873 | Alpha tocopherol transfer protein like | [138] |
| *TUBA1A* | ENSBTAG00000001489 | Tubulin, alpha 1a | [138] |
| *TUBA1B* | ENSBTAG00000012244 | Tubulin, alpha 1b | [138] |
| *TUBA1C* | ENSBTAG00000039992 | Tubulin alpha 1c | [138] |
| *TUBB2A* | ENSBTAG00000048485 | Tubulin beta 2A class IIa | [140] |
| *TUBB4A* | ENSBTAG00000021013 | Tubulin beta 4A class IVa | [140] |
| *TUBGCP3* | ENSBTAG00000044184 | Tubulin gamma complex associated protein 3 | [203] |
| *TULP2* | ENSBTAG00000002368 | TUB like protein 2 | [203] |
| *TUT7* | ENSBTAG00000001737 | Terminal uridylyl transferase 7 | [203] |
| *TWF2* | ENSBTAG00000054131 | Twinfilin actin binding protein 2 | [51] |
| *TXN* | ENSBTAG00000002953 | Thioredoxin | [138] |
| *TXNDC17* | ENSBTAG00000054776 | Thioredoxin domain containing 17 | [138] |
| *TYMS* | ENSBTAG00000007003 | Thymidylate synthetase | [139] |
| *TYROBP* | ENSBTAG00000007338 | Transmembrane immune signaling adaptor TYROBP | [51] |
| *TYRP1* | ENSBTAG00000020985 | Tyrosinase related protein 1 | [203] |
| *U2AF1* | ENSBTAG00000011645 | U2 small nuclear RNA auxiliary factor 1 | [140] |
| *UBA7* | ENSBTAG00000012335 | Ubiquitin like modifier activating enzyme 7 | [139] |
| *UBAC2* | ENSBTAG00000005682 | UBA domain containing 2 | [140] |
| *UBE2L6* | ENSBTAG00000012989 | Ubiquitin conjugating enzyme E2 L6 | [139] |
| *UBE4B* | ENSBTAG00000010401 | Ubiquitination factor E4B | [203] |
| *UCHL1* | ENSBTAG00000005078 | Ubiquitin C-terminal hydrolase L1 | [140] |
| *UCP2* | ENSBTAG00000003692 | Uncoupling protein 2 | [138] |
| *UGDH* | ENSBTAG00000014521 | UDP-glucose 6-dehydrogenase | [51] |
| *UGT2B10* | ENSBTAG00000039991 | UDP glucuronosyltransferase 2 family, polypeptide B10 | [203] |
| *UHRF1* | ENSBTAG00000002224 | Ubiquitin like with PHD and ring finger domains 1 | [139] |
| *UHRF2* | ENSBTAG00000020815 | Ubiquitin like with PHD and ring finger domains 2 | [140] |
| *UMPS* | ENSBTAG00000013727 | Uridine monophosphate synthetase | [140] |
| *UNC119B* | ENSBTAG00000007483 | Unc-119 lipid binding chaperone B | [203] |
| *UNC5B* | ENSBTAG00000014270 | Unc-5 netrin receptor B | [203] |
| *UNC93A* | ENSBTAG00000014498 | Unc-93 homolog A | [139] |
| *UOX* | ENSBTAG00000024255 | Urate oxidase | [203] |
| *UPF2* | ENSBTAG00000000288 | UPF2 regulator of nonsense mediated mRNA decay | [139] |
| *UPK3B* | ENSBTAG00000021430 | Uroplakin 3B | [203] |
| *UPP1* | ENSBTAG00000008428 | Uridine phosphorylase 1 | [51] |
| *UROS* | ENSBTAG00000005791 | Uroporphyrinogen III synthase | [203] |
| *USP1* | ENSBTAG00000020451 | Ubiquitin specific peptidase 1 | [138] |
| *USP11* | ENSBTAG00000016772 | Ubiquitin specific peptidase 11 | [203] |
| *USP15* | ENSBTAG00000010428 | Ubiquitin specific peptidase 15 | [139] |
| *USP18* | ENSBTAG00000016661 | Ubiquitin specific peptidase 18 | [139] |
| *USP2* | ENSBTAG00000009749 | Ubiquitin specific peptidase 2 | [203] |
| *USP24* | ENSBTAG00000016990 | Ubiquitin specific peptidase 24 | [203] |
| *USP25* | ENSBTAG00000019314 | Ubiquitin specific peptidase 25 | [138] |
| *USP36* | ENSBTAG00000021505 | Ubiquitin specific peptidase 36 | [203] |
| *USP38* | ENSBTAG00000001356 | Ubiquitin specific peptidase 38 | [203] |
| *USP49* | ENSBTAG00000008981 | Ubiquitin specific peptidase 49 | [203] |
| *USP8* | ENSBTAG00000011916 | Ubiquitin specific peptidase 8 | [139] |
| *UTP14A* | ENSBTAG00000013671 | Bos taurus UTP14, U3 small nucleolar ribonucleoprotein, homolog A-like (LOC505600), mRNA | [204] |
| *UTP18* | ENSBTAG00000020629 | UTP18 small subunit processome component | [140] |
| *UTP6* | ENSBTAG00000015612 | UTP6 small subunit processome component | [140] |
| *UVSSA* | ENSBTAG00000012577 | UV stimulated scaffold protein A | [203] |
| *VAMP8* | ENSBTAG00000023997 | Vesicle associated membrane protein 8 | [138] |
| *VAPA* | ENSBTAG00000017279 | VAMP associated protein A | [140] |
| *VAPB* | ENSBTAG00000017424 | VAMP associated protein B and C | [140] |
| *VASH1* | ENSBTAG00000008735 | Vasohibin 1 | [203] |
| *VAT1L* | ENSBTAG00000018146 | Vesicle amine transport 1 like | [203] |
| *VAV1* | ENSBTAG00000039160 | Vav guanine nucleotide exchange factor 1 | [51] |
| *VCAM1* | ENSBTAG00000007773 | Vascular cell adhesion molecule 1 | [139] |
| *VCAN* | ENSBTAG00000014906 | Versican | [139] |
| *VCPIP1* | ENSBTAG00000006695 | Valosin containing protein interacting protein 1 | [140] |
| *VEGFA* | ENSBTAG00000005339 | Vascular endothelial growth factor A | [139] |
| *VEGFB* | ENSBTAG00000047567 | Vascular endothelial growth factor B | [140] |
| *VEGFC* | ENSBTAG00000014737 | Vascular endothelial growth factor C | [139] |
| *VIL1* | ENSBTAG00000018543 | Villin 1 | [203] |
| *VILL* | ENSBTAG00000012963 | Villin like | [203] |
| *VIM* | ENSBTAG00000018463 | Vimentin | [204] |
| *VIPAS39* | ENSBTAG00000020475 | VPS33B interacting protein, apical-basolateral polarity regulator, spe-39 homolog | [203] |
| *VLDLR* | ENSBTAG00000018517 | Very low density lipoprotein receptor | [52] |
| *VMP1* | ENSBTAG00000011623 | Vacuole membrane protein 1 | [203] |
| *VPS13C* | ENSBTAG00000038920 | Vacuolar protein sorting 13 homolog C | [139] |
| *VPS13D* | ENSBTAG00000016080 | Vacuolar protein sorting 13 homolog D | [203] |
| *VRTN* | ENSBTAG00000005774 | Vertebrae development associated | [203] |
| *VSTM1* | ENSBTAG00000002596 | V-set and transmembrane domain containing 1 | [51] |
| *VSX2* | ENSBTAG00000014632 | Visual system homeobox 2 | [203] |
| *VTI1B* | ENSBTAG00000001948 | Vesicle transport through interaction with t-SNAREs 1B | [140] |
| *VWA1* | ENSBTAG00000021294 | Von Willebrand factor A domain containing 1 | [203] |
| *WARS1* | ENSBTAG00000004679 | Tryptophanyl-tRNA synthetase 1 | [139] |
| *WDFY2* | ENSBTAG00000008053 | WD repeat and FYVE domain containing 2 | [203] |
| *WDR12* | ENSBTAG00000015424 | WD repeat domain 12 | [138] |
| *WDR19* | ENSBTAG00000014512 | WD repeat domain 19 | [203] |
| *WDR33* | ENSBTAG00000005987 | WD repeat domain 33 | [203] |
| *WDR64* | ENSBTAG00000005200 | WD repeat domain 64 | [203] |
| *WDR86* | ENSBTAG00000006232 | WD repeat domain 86 | [203] |
| *WDSUB1* | ENSBTAG00000004184 | WD repeat, sterile alpha motif and U-box domain containing 1 | [140] |
| *WIPF1* | ENSBTAG00000001585 | WAS/WASL interacting protein family member 1 | [204] |
| *WIPI1* | ENSBTAG00000039556 | WD repeat domain, phosphoinositide interacting 1 | [204] |
| *WRN* | ENSBTAG00000021592 | WRN RecQ like helicase | [203] |
| *WSB1* | ENSBTAG00000005008 | WD repeat and SOCS box containing 1 | [140] |
| *WTAP* | ENSBTAG00000007974 | WT1 associated protein | [51] |
| *XAF1* | ENSBTAG00000011343 | XIAP associated factor 1 | [139] |
| *XDH* | ENSBTAG00000012519 | Xanthine dehydrogenase | [139] |
| *XPC* | ENSBTAG00000007362 | XPC complex subunit, DNA damage recognition and repair factor | [203] |
| *XRCC2* | ENSBTAG00000030897 | X-ray repair cross complementing 2 | [138] |
| *XRN2* | ENSBTAG00000011304 | 5,-3, exoribonuclease 2 | [140] |
| *YBX3* | ENSBTAG00000009663 | Y-box binding protein 3 | [203] |
| *YIF1B* | ENSBTAG00000027711 | Yip1 interacting factor homolog B, membrane trafficking protein | [140] |
| *YKT6* | ENSBTAG00000000274 | YKT6 v-SNARE homolog | [138] |
| *YME1L1* | ENSBTAG00000016445 | YME1 like 1 ATPase | [139] |
| *YPEL5* | ENSBTAG00000023744 | Yippee like 5 | [139] |
| *ZBTB1* | ENSBTAG00000040551 | Zinc finger and BTB domain containing 1 | [139] |
| *ZBTB11* | ENSBTAG00000013460 | Zinc finger and BTB domain containing 11 | [203] |
| *ZBTB7B* | ENSBTAG00000021512 | Zinc finger and BTB domain containing 7B | [138] |
| *ZBTB8B* | ENSBTAG00000046044 | Zinc finger and BTB domain containing 8B | [203] |
| *ZC3H12A* | ENSBTAG00000011316 | Zinc finger CCCH-type containing 12A | [51] |
| *ZC3H14* | ENSBTAG00000030453 | Zinc finger CCCH-type containing 14 | [203] |
| *ZC3H15* | ENSBTAG00000021762 | Zinc finger CCCH-type containing 15 | [139] |
| *ZC3H7A* | ENSBTAG00000014826 | Zinc finger CCCH-type containing 7A | [140] |
| *ZC3H8* | ENSBTAG00000015965 | Zinc finger CCCH-type containing 8 | [139] |
| *ZC4H2* | ENSBTAG00000003779 | Zinc finger C4H2-type containing | [138] |
| *ZCCHC17* | ENSBTAG00000016818 | Zinc finger CCHC-type containing 17 | [204] |
| *ZDHHC14* | ENSBTAG00000018464 | Zinc finger DHHC-type palmitoyltransferase 14 | [203] |
| *ZDHHC22* | ENSBTAG00000014384 | Zinc finger DHHC-type palmitoyltransferase 22 | [203] |
| *ZFAT* | ENSBTAG00000017731 | Zinc finger and AT-hook domain containing | [203] |
| *ZFC3H1* | ENSBTAG00000012267 | Zinc finger C3H1-type containing | [203] |
| *ZFP36* | ENSBTAG00000008573 | ZFP36 ring finger protein | [138] |
| *ZFP62* | ENSBTAG00000039711 | ZFP62 zinc finger protein | [203] |
| *ZFR* | ENSBTAG00000008414 | Zinc finger RNA binding protein | [140] |
| *ZFX* | ENSBTAG00000007730 | Zinc finger protein X-linked | [140] |
| *ZFYVE1* | ENSBTAG00000011041 | Zinc finger FYVE-type containing 1 | [203] |
| *ZFYVE26* | ENSBTAG00000014334 | Zinc finger FYVE-type containing 26 | [203] |
| *ZMIZ2* | ENSBTAG00000011997 | Zinc finger MIZ-type containing 2 | [139] |
| *ZMYND15* | ENSBTAG00000018316 | Zinc finger MYND-type containing 15 | [139] |
| *ZNF146* | ENSBTAG00000031687 | Zinc finger protein 146 | [139] |
| *ZNF160* | ENSBTAG00000054322 | Zinc finger protein 665 | [139] |
| *ZNF180* | ENSBTAG00000006805 | Zinc finger protein 180 | [203] |
| *ZNF181* | ENSBTAG00000009889 | Zinc finger protein 181 | [139] |
| *ZNF184* | ENSBTAG00000011498 | Zinc finger protein 184 | [203] |
| *ZNF227* | ENSBTAG00000027431 | Zinc finger protein 227 | [203] |
| *ZNF292* | ENSBTAG00000046612 | Zinc finger protein 292 | [139] |
| *ZNF295* | ENSBTAG00000030836 | Zinc finger and BTB domain containing 21 | [203] |
| *ZNF354A* | ENSBTAG00000006428 | Zinc finger protein 354A | [139] |
| *ZNF354B* | ENSBTAG00000017123 | Zinc finger protein 93 | [203] |
| *ZNF37A* | ENSBTAG00000051862 | Zinc finger protein 37A | [203] |
| *ZNF382* | ENSBTAG00000008084 | Zinc finger protein 382 | [203] |
| *ZNF385B* | ENSBTAG00000003120 | Zinc finger protein 385B | [203] |
| *ZNF389* | ENSBTAG00000051500 | Zinc finger protein 389 | [203] |
| *ZNF410* | ENSBTAG00000020327 | Zinc finger protein 410 | [203] |
| *ZNF462* | ENSBTAG00000019187 | Zinc finger protein 462 | [203] |
| *ZNF518A* | ENSBTAG00000021357 | Zinc finger protein 518A | [139] |
| *ZNF527* | ENSBTAG00000039075 | Zinc finger protein 527 | [203] |
| *ZNF555* | ENSBTAG00000008397 | Zinc finger protein 555 | [203] |
| *ZNF567* | ENSBTAG00000002398 | Zinc finger protein 567 | [139] |
| *ZNF638* | ENSBTAG00000009242 | Zinc finger protein 638 | [203] |
| *ZNF654* | ENSBTAG00000023675 | Zinc finger protein 654 | [139] |
| *ZNF672* | ENSBTAG00000046984 | Zinc finger protein 672 | [203] |
| *ZNF674* | ENSBTAG00000002956 | Zinc finger protein 674 | [203] |
| *ZNF689* | ENSBTAG00000018094 | Zinc finger protein 689 | [203] |
| *ZNF710* | ENSBTAG00000019174 | Zinc finger protein 710 | [203] |
| *ZNF740* | ENSBTAG00000046481 | Zinc finger protein 740 | [203] |
| *ZNF75D* | ENSBTAG00000047939 | Zinc finger protein 75D | [140] |
| *ZNF770* | ENSBTAG00000040206 | Zinc finger protein 770 | [203] |
| *ZNF784* | ENSBTAG00000016299 | Zinc finger protein 784 | [203] |
| *ZNFX1* | ENSBTAG00000020166 | Zinc finger NFX1-type containing 1 | [139] |
| *ZNHIT3* | ENSBTAG00000012672 | Zinc finger HIT-type containing 3 | [140] |
| *ZRANB1* | ENSBTAG00000003395 | Zinc finger RANBP2-type containing 1 | [203] |
| *ZRANB3* | ENSBTAG00000026842 | Zinc finger RANBP2-type containing 3 | [203] |
| *ZSWIM4* | ENSBTAG00000002527 | Zinc finger SWIM-type containing 4 | [203] |
| *ZUFSP* | ENSBTAG00000003878 | Zinc finger containing ubiquitin peptidase 1 | [139] |
| *ZWINT* | ENSBTAG00000002655 | ZW10 interacting kinetochore protein | [139] |

**References**

1. Atabai K, Fernandez R, Huang X, Ueki I, Kline A, Li Y, et al. Mfge8 is critical for mammary gland remodeling during involution. Mol Biology Cell. 2005;16(12):5528–37.

2. Liu S, Umezu-Goto M, Murph M, Lu Y, Liu W, Zhang F, et al. Expression of autotaxin and lysophosphatidic acid receptors increases mammary tumorigenesis, invasion, and metastases. Cancer Cell. 2009;15(6):539–50.

3. Nagaoka K, Aoki F, Hayashi M, Muroi Y, Sakurai T, Itoh K, et al. L-amino acid oxidase plays a crucial role in host defense in the mammary glands. FASEB J. 2009;23(8):2514–20.

4. Wang X, Huang J, Zhao L, Wang C, Ju Z, Li Q, et al. The exon 29 c.3535A>T in the alpha-2-macroglobulin gene causing aberrant splice variants is associated with mastitis in dairy cattle. Immunogenetics. 2012;64(11):807–16.

5. Wang XG, Huang JM, Feng MY, Ju ZH, Wang CF, Yang GW, et al. Regulatory mutations in the A2M gene are involved in the mastitis susceptibility in dairy cows. Anim Genet. 2014;45(1):28–37.

6. Kirsanova E, Boysen P, Johansen GM, Heringstad B, Lewandowska-Sabat A, Olsaker I. Expression analysis of candidate genes for chronic subclinical mastitis in Norwegian Red cattle. J Dairy Sci. 2020;103(10):9142–9.

7. Nayeri S, Schenkel F, Fleming A, Kroezen V, Sargolzaei M, Baes C, et al. Genome-wide association analysis for β-hydroxybutyrate concentration in milk in Holstein dairy cattle. BMC Genet. 2019;20(1):58.

8. Costa A, Schwarzenbacher H, Mészáros G, Fuerst-Waltl B, Fuerst C, Sölkner J, et al. On the genomic regions associated with milk lactose in Fleckvieh cattle. J Dairy Sci. 2019;102(11):10088–99.

9. Tiezzi F, Parker-Gaddis KL, Cole JB, Clay JS, Maltecca C. A genome-wide association study for clinical mastitis in first parity US Holstein cows using single-step approach and genomic matrix re-weighting procedure. PLoS One. 2015;10(2):e0114919.

10. Szyda J, Mielczarek M, Frąszczak M, Minozzi G, Williams JL, Wojdak-Maksymiec K. The genetic background of clinical mastitis in Holstein-Friesian cattle. Animal. 2019;13(10):2156–63.

11. Meredith BK, Berry DP, Kearney F, Finlay EK, Fahey AG, Bradley DG, et al. A genome-wide association study for somatic cell score using the Illumina high-density bovine beadchip identifies several novel QTL potentially related to mastitis susceptibility. Front Genet. 2013;4:229.

12. Wang X, Ma P, Liu J, Zhang Q, Zhang Y, Ding X, et al. Genome-wide association study in Chinese Holstein cows reveal two candidate genes for somatic cell score as an indicator for mastitis susceptibility. BMC Genet. 2015;16:111.

13. Siebert L, Staton ME, Headrick S, Lewis M, Gillespie B, Young C, et al. Genome-wide association study identifies loci associated with milk leukocyte phenotypes following experimental challenge with Streptococcus uberis. Immunogenetics. 2018;70(9):553–62.

14. Welderufael BG, Løvendahl P, de Koning DJ, Janss LLG, Fikse WF. Genome-wide association study for susceptibility to and recoverability from mastitis in Danish Holstein cows. Front Genet. 2018;9:141.

15. Liu YX, Xu CH, Gao TY, Sun Y. Polymorphisms of the ATP1A1 gene associated with mastitis in dairy cattle. Genet Mol Res. 2012;11(1):651–60.

16. Hou Q, Huang J, Ju Z, Li Q, Li L, Wang C, et al. Identification of splice variants, targeted microRNAs and functional single nucleotide polymorphisms of the BOLA-DQA2 gene in dairy cattle. DNA Cell Biol. 2012;31(5):739–44.

17. Kelm SC, Detilleux JC, Freeman AE, Kehrli ME, Dietz AB, Fox LK, et al. Genetic association between parameters of inmate immunity and measures of mastitis in periparturient Holstein cattle. J Dairy Sci. 1997;80(8):1767–75.

18. Yoshida T, Mukoyama H, Furuta H, Kondo Y, Takeshima SN, Aida Y, et al. Association of BoLA-DRB3 alleles identified by a sequence-based typing method with mastitis pathogens in Japanese Holstein cows. Anim Sci J. 2009;80(5):498–509.

19. Yoshida T, Furuta H, Kondo Y, Mukoyama H. Association of BoLA-DRB3 alleles with mastitis resistance and susceptibility in Japanese Holstein cows. Anim Sci J. 2012;83(5):359–66.

20. Baltian LR, Ripoli MV, Sanfilippo S, Takeshima SN, Aida Y, Giovambattista G. Association between BoLA-DRB3 and somatic cell count in Holstein cattle from Argentina. Mol Biol Rep. 2012;39(7):7215–20.

21. Pokorska J, Kułaj D, Dusza M, Ochrem A, Makulska J. The influence of BoLA-DRB3 alleles on incidence of clinical mastitis, cystic ovary disease and milk traits in Holstein Friesian cattle. Mol Biol Rep. 2018;45(5):917–23.

22. Rupp R, Hernandez A, Mallard BA. Association of bovine leukocyte antigen (BoLA) DRB3.2 with immune response, mastitis, and production and type traits in Canadian Holsteins. J Dairy Sci. 2007;90(2):1029–38.

23. Kulberg S, Heringstad B, Guttersrud OA, Olsaker I. Study on the association of BoLA-DRB3.2 alleles with clinical mastitis in Norwegian Red cows. J Anim Breed Genet. 2007;124(4):201–7.

24. Prajapati BM, Gupta JP, Pandey DP, Parmar GA, Chaudhari JD. Molecular markers for resistance against infectious diseases of economic importance. Vet World. 2017;10(1):112–20.

25. Sharif S, Mallard BA, Wilkie BN, Sargeant JM, Scott HM, Dekkers JC, et al. Associations of the bovine major histocompatibility complex DRB3 (BoLA-DRB3) alleles with occurrence of disease and milk somatic cell score in Canadian dairy cattle. Anim Genet. 1998;29(3):185–93.

26. Sharif S, Mallard BA, Wilkie BN, Sargeant JM, Scott HM, Dekkers JC, et al. Associations of the bovine major histocompatibility complex DRB3 (BoLA-DRB3) with production traits in Canadian dairy cattle. Anim Genet. 1999;30(2):157–60.

27. Sharif S, Mallard BA, Sargeant JM. Presence of glutamine at position 74 of pocket 4 in the BoLA-DR antigen binding groove is associated with occurrence of clinical mastitis caused by Staphylococcus species. Vet Immunol Immunopathol. 2000;76(3-4):231–8.

28. Ogorevc J, Kunej T, Razpet A, Dovc P. Database of cattle candidate genes and genetic markers for milk production and mastitis. Anim Genet. 2009;40(6):832–51.

29. Derakhshani H, Plaizier JC, De Buck J, Barkema HW, Khafipour E. Association of bovine major histocompatibility complex (BoLA) gene polymorphism with colostrum and milk microbiota of dairy cows during the first week of lactation. Microbiome. 2018;6(1):203.

30. Oprzadek J, Sender G, Pawlik A, Lukaszewicz M. Locus BoLA-DRB3 is just an ordinary site of the polygene when explaining genetic variance of somatic cell count and milk yield. J Dairy Res. 2015;82(4):449–52.

31. Yuan Z, Li J, Zhang L, Gao X, Gao HJ, Xu S. Investigation on BRCA1 SNPs and its effects on mastitis in Chinese commercial cattle. Gene. 2012;505(1):190–4.

32. Magotra A, Gupta ID, Ahmad T, Alex R. Polymorphism in DNA repair gene BRCA1 associated with clinical mastitis and production traits in indigenous dairy cattle. Res Vet Sci. 2020;133:194–201.

33. El-Halawany N, Abd-El-Monsif SA, Al-Tohamy Ahmed FM, Hegazy L, Abdel-Shafy H, Abdel-Latif MA, et al. Complement component 3: characterization and association with mastitis resistance in Egyptian water buffalo and cattle. J Genet. 2017;96(1):65–73.

34. Banos G, Bramis G, Bush SJ, Clark EL, McCulloch MEB, Smith J, et al. The genomic architecture of mastitis resistance in dairy sheep. BMC Genom. 2017;18(1):624.

35. Mitterhuemer S, Petzl W, Krebs S, Mehne D, Klanner A, Wolf E, et al. Escherichia coli infection induces distinct local and systemic transcriptome responses in the mammary gland. BMC Genom. 2010;11:138.

36. Sahana G, Guldbrandtsen B, Thomsen B, Lund MS. Confirmation and fine-mapping of clinical mastitis and somatic cell score QTL in Nordic Holstein cattle. Anim Genet. 2013;44(6):620–6.

37. Olsen HG, Knutsen TM, Lewandowska-Sabat AM, Grove H, Nome T, Svendsen M, et al. Fine mapping of a QTL on bovine chromosome 6 using imputed full sequence data suggests a key role for the group-specific component (GC) gene in clinical mastitis and milk production. Genet Sel Evol. 2016;48(1):79.

38. Yuan ZR, Li J, Liu L, Zhang LP, Zhang LM, Chen C, et al. Single nucleotide polymorphism of CACNA2D1 gene and its association with milk somatic cell score in cattle. Mol Biol Rep. 2011;38(8):5179–83.

39. Silva AA, Silva DA, Silva FF, Costa CN, Silva HT, Lopes PS, et al. GWAS and gene networks for milk-related traits from test-day multiple lactations in Portuguese Holstein cattle. J Appl Genet. 2020;61(3):465–76.

40. Magotra A, Gupta ID, Verma A, Alex R, Mr V, Ahmad T. Candidate SNP of CACNA2D1 gene associated with clinical mastitis and production traits in Sahiwal (Bos taurus indicus) and Karan Fries (Bos taurus taurus × Bos taurus indicus). Anim Biotechnol. 2019;30(1):75–81.

41. Deb R, Singh U, Kumar S, Kumar A, Singh R, Sengar G, et al. Genotypic to expression profiling of bovine calcium channel, voltage-dependent, alpha-2/delta subunit 1 gene, and their association with bovine mastitis among Frieswal (HFX Sahiwal) crossbred cattle of Indian origin. Anim Biotechnol. 2014;25(2):128–38.

42. Bagheri M, Zahmatkesh A. Dominance effects estimation of TLR4 and CACNA2D1 genes for health and production traits using logistic regression. J Genet. 2017;96(6):1027–31.

43. Kadri NK, Guldbrandtsen B, Lund MS, Sahana G. Genetic dissection of milk yield traits and mastitis resistance quantitative trait loci on chromosome 20 in dairy cattle. J Dairy Sci. 2015;98(12):9015–25.

44. Yang L, Guo R, Ju Z, Wang X, Jiang Q, Liu Y, et al. Production of an aberrant splice variant of CCL5 is not caused by genetic mutation in the mammary glands of mastitis‑infected Holstein cows. Mol Med Rep. 2019;19(5):4159–66.

45. Kirsanova E, Heringstad B, Lewandowska-Sabat A, Olsaker I. Identification of candidate genes affecting chronic subclinical mastitis in Norwegian Red cattle: combining genome-wide association study, topologically associated domains and pathway enrichment analysis. Animal Genet. 2020;51(1):22–31.

46. Leyva-Baca I, Schenkel F, Sharma BS, Jansen GB, Karrow NA. Identification of single nucleotide polymorphisms in the bovine CCL2, IL8, CCR2 and IL8RA genes and their association with health and production in Canadian Holsteins. Anim Genet. 2007;38(3):198–202.

47. Huang JM, Wang XG, Jiang Q, Sun Y, Yang CH, Ju ZH, et al. Identification of CD14 transcript in blood polymorphonuclear neutrophil leukocytes and functional variation in Holsteins. Genet Mol Res. 2016;15(2): gmr.15027932.

48. Selvan AS, Gupta ID, Verma A, Chaudhari MV, Magotra A. Molecular characterization and combined genotype association study of bovine cluster of differentiation 14 gene with clinical mastitis in crossbred dairy cattle. Vet World. 2016;9(7):680–4.

49. Wang Y, Zarlenga DS, Paape MJ, Dahl GE. Recombinant bovine soluble CD14 sensitizes the mammary gland to lipopolysaccharide. Vet Immunol Immunopathol. 2002;86(1):115–24.

50. Li J, Wang Q, Chen F, Wang H, Chen J, Wang Z, et al. SNPs of CD14 change the mastitis morbidity of Chinese Holstein. Mol Med Rep. 2017;16(6):9102–10.

51. Buitenhuis B, Røntved CM, Edwards SM, Ingvartsen KL, Sørensen P. In depth analysis of genes and pathways of the mammary gland involved in the pathogenesis of bovine Escherichia coli-mastitis. BMC Genom. 2011;12:130.

52. Younis S, Javed Q, Blumenberg M. Meta-analysis of transcriptional responses to mastitis-causing Escherichia coli. PLoS One. 2016;11(3):e0148562.

53. He Y, Chu Q, Ma P, Wang Y, Zhang Q, Sun D, et al. Association of bovine CD4 and STAT5b single nucleotide polymorphisms with somatic cell scores and milk production traits in Chinese Holsteins. J Dairy Res. 2011;78(2):242–9.

54. Wang X, Zhong J, Gao Y, Ju Z, Huang J. A SNP in intron 8 of CD46 causes a novel transcript associated with mastitis in Holsteins. BMC Genom. 2014;15:630.

55. Wang Z, Huang J, Zhong J, Wang G. Molecular cloning, promoter analysis, SNP detection of Clusterin gene and their associations with mastitis in Chinese Holstein cows. Mol Biol Rep. 2012;39(3):2439–45.

56. Sodeland M, Grove H, Kent M, Taylor S, Svendsen M, Hayes BJ, et al. Molecular characterization of a long range haplotype affecting protein yield and mastitis susceptibility in Norwegian Red cattle. BMC Genet. 2011;12:70.

57. Sodeland M, Kent MP, Olsen HG, Opsal MA, Svendsen M, Sehested E, et al. Quantitative trait loci for clinical mastitis on chromosomes 2, 6, 14 and 20 in Norwegian Red cattle. Anim Genet. 2011;42(5):457–65.

58. Nani JP, Raschia MA, Carignano H, Poli MA, Calvinho LF, Amadio AF. Single nucleotide polymorphisms in candidate genes and their relation with somatic cell scores in Argentinean dairy cattle. J Appl Genet. 2015;56(4):505–13.

59. Sharifi S, Pakdel A, Ebrahimi M, Reecy JM, Fazeli Farsani S, Ebrahimie E. Integration of machine learning and meta-analysis identifies the transcriptomic bio-signature of mastitis disease in cattle. PLoS One. 2018;13(2):e0191227.

60. Gorji AE, Roudbari Z, Sadeghi B, Javadmanesh A, Sadkowski T. Transcriptomic analysis on the promoter regions discover gene networks involving mastitis in cattle. Microb Pathog. 2019;137:103801.

61. Dai H, Wei G, Wang Y, Ma N, Chang G, Shen X. Sodium butyrate promotes lipopolysaccharide-induced innate immune responses by enhancing mitogen-activated protein kinase activation and histone acetylation in bovine mammary epithelial cells. J Dairy Sci. 2020;103(12):11636–52.

62. Rainard P, Cunha P, Bougarn S, Fromageau A, Rossignol C, Gilbert FB, et al. T helper 17-associated cytokines are produced during antigen-specific inflammation in the mammary gland. PLoS One. 2013;8(5):e63471.

63. Russell CD, Widdison S, Leigh JA, Coffey TJ. Identification of single nucleotide polymorphisms in the bovine Toll-like receptor 1 gene and association with health traits in cattle. Vet Res. 2012;43:17.

64. Barber MR, Yang TJ. Chemotactic activities in nonmastitic and mastitic mammary secretions: presence of interleukin-8 in mastitic but not nonmastitic secretions. Clin Diag Lab Immunol. 1998;5(1):82–6.

65. Nagahata H, Moriyama A, Sawada C, Asai Y, Kokubu C, Gondaira S, et al. Innate immune response of mammary gland induced by intramammary infusion of Bifidobacterium breve in lactating dairy cows. J Vet Med Sci. 2020;82(12):1742–9.

66. Chen R, Yang Z, Ji D, Mao Y, Chen Y, Li Y, et al. Polymorphisms of the IL8 gene correlate with milking traits, SCS and mRNA level in Chinese Holstein. Mol Biol Rep. 2011;38(6):4083–8.

67. Chen R, Wang Z, Yang Z, Zhu X, Ji D, Mao Y. Association of IL8 -105G/A with mastitis somatic cell score in Chinese Holstein dairy cows. Anim Biotechnol. 2015;26(2):143–7.

68. Purba FY, Suzuki N, Isobe N. Association of endometritis and ovarian follicular cyst with mastitis in dairy cows. J Vet Med Sci. 2021;83(2):338–43.

69. Hughes K, Watson CJ. The mammary microenvironment in mastitis in humans, dairy ruminants, rabbits and rodents: A one health focus. J Mammary Gland Biol Neoplasia. 2018;23(1-2):27–41.

70. Stojkovic B, Mullen MP, Donofrio G, McLoughlin RM, Meade KG. Interleukin 8 haplotypes drive divergent responses in uterine endometrial cells and are associated with somatic cell score in Holstein-Friesian cattle. Vet Immunol Immunopathol. 2017;184:18–28.

71. Deng Z, Shahid M, Zhang L, Gao J, Gu X, Zhang S, et al. An investigation of the innate immune response in bovine mammary epithelial cells challenged by Prototheca zopfii. Mycopathologia. 2016;181(11-12):823–32.

72. Zaatout N. An overview on mastitis-associated Escherichia coli: Pathogenicity, host immunity and the use of alternative therapies. Microbiol Res. 2022;256:126960.

73. Bohl LP, Isaac P, Breser ML, Orellano MS, Correa SG, Tolosa de Talamoni NG, et al. Interaction between bovine mammary epithelial cells and planktonic or biofilm Staphylococcus aureus: The bacterial lifestyle determines its internalization ability and the pathogen recognition. Microb Pathog. 2021;152:104604.

74. El-Deeb W, Fayez M, Alhumam N, Elsohaby I, Quadri SA, Mkrtchyan H. The effect of staphylococcal mastitis including resistant strains on serum procalcitonin, neopterin, acute phase response and stress biomarkers in Holstein dairy cows. PeerJ. 2021;9:e11511.

75. Mount JA, Karrow NA, Caswell JL, Boermans HJ, Leslie KE. Assessment of bovine mammary chemokine gene expression in response to lipopolysaccharide, lipotechoic acid + peptidoglycan, and CpG oligodeoxynucleotide 2135. Can J Vet Res. 2009;73(1):49–57.

76. Fonseca I, Silva PV, Lange CC, Guimarães MFM, Weller MMDCA, Sousa KRS, et al. Expression profile of genes associated with mastitis in dairy cattle. Genet Mol Biol. 2009;32(4):776–81.

77. Tao W, Mallard B. Differentially expressed genes associated with Staphylococcus aureus mastitis of Canadian Holstein cows. Vet Immunol Immunopathol. 2007;120(3-4):201–11.

78. Moyes KM, Drackley JK, Morin DE, Rodriguez-Zas SL, Everts RE, Lewin HA, et al. Mammary gene expression profiles during an intramammary challenge reveal potential mechanisms linking negative energy balance with impaired immune response. Physiol Genomics. 2010;41(2):161–70.

79. Niedziela DA, Murphy MP, Grant J, Keane OM, Leonard FC. Clinical presentation and immune characteristics in first-lactation Holstein-Friesian cows following intramammary infection with genotypically distinct Staphylococcus aureus strains. J Dairy Sci. 2020;103(9):8453–66.

80. Stevens MG, Peelman LJ, De Spiegeleer B, Pezeshki A, Van De Walle GR, Duchateau L, et al. Differential gene expression of the toll-like receptor-4 cascade and neutrophil function in early- and mid-lactating dairy cows. J Dairy Sci. 2011;94(3):1277–88.

81. Fu Y, Zhou E, Liu Z, Li F, Liang D, Liu B, et al. Staphylococcus aureus and Escherichia coli elicit different innate immune responses from bovine mammary epithelial cells. Vet Immunol Immunopathol. 2013;155(4):245–52.

82. Tsuchida S, Yamad Y, Fukui E, Kawada T, Omi T, Tsuchida A, et al. Distribution of single nucleotide polymorphisms in the CXCR1 gene and association with calf diseases in Japanese Black cattle. J Vet Med Sci. 2010;72(12):1609–14.

83. Galvão KN, Pighetti GM, Cheong SH, Nydam DV, Gilbert RO. Association between interleukin-8 receptor-α (CXCR1) polymorphism and disease incidence, production, reproduction, and survival in Holstein cows. J Dairy Sci. 2011;94(4):2083–91.

84. Verbeke J, Piepers S, Peelman L, Van Poucke M, De Vliegher S. Pathogen-group specific association between CXCR1 polymorphisms and subclinical mastitis in dairy heifers. J Dairy Res. 2012;79(3):341–51.

85. Verbeke J, Van Poucke M, Peelman L, Piepers S, De Vliegher S. Associations between CXCR1 polymorphisms and pathogen-specific incidence rate of clinical mastitis, test-day somatic cell count, and test-day milk yield. J Dairy Sci. 2014;97(12):7927–39.

86. Verbeke J, Van Poucke M, Peelman L, De Vliegher S. Differential expression of CXCR1 and commonly used reference genes in bovine milk somatic cells following experimental intramammary challenge. BMC Genet. 2015;16:40.

87. Verbeke J, Boulougouris X, Rogiers C, Burvenich C, Peelman L, De Spiegeleer B, et al. Reactive oxygen species generation by bovine blood neutrophils with different CXCR1 (IL8RA) genotype following Interleukin-8 incubation. BMC Vet Res. 2015;11:104.

88. Verbeke J, Piccart K, Piepers S, Van Poucke M, Peelman L, De Visscher A, et al. Somatic cell count and milk neutrophil viability of dairy heifers with specific CXCR1 genotypes following experimental intramammary infection with Staphylococcus chromogenes originating from milk. Vet J. 2015;204(3):322–6.

89. Zhou L, Wang HM, Ju ZH, Zhang Y, Huang JM, Qi C, et al. Association of novel single nucleotide polymorphisms of the CXCR1 gene with the milk performance traits of Chinese native cattle. Genet Mol Res. 2013;12(3):2725–39.

90. Bagheri M, Moradi-Sharhrbabak M, Miraie-Ashtiani R, Safdari-Shahroudi M, Abdollahi-Arpanahi R. Case-control approach application for finding a relationship between candidate genes and clinical mastitis in Holstein dairy cattle. J Appl Genet. 2016;57(1):107–12.

91. Pokorska J, Dusza M, Kułaj D, Żukowski K, Makulska J. Single nucleotide polymorphisms in the CXCR1 gene and its association with clinical mastitis incidence in Polish Holstein-Friesian cows. Genet Mol Res. 2016;15(2): gmr.15027247.

92. Pawlik A, Sender G, Kapera M, Korwin-Kossakowska A. Association between interleukin 8 receptor α gene (CXCR1) and mastitis in dairy cattle. Cent-Europ J Immunol. 2015;40(2):153–8.

93. Rambeaud M, Pighetti GM. Differential calcium signaling in dairy cows with specific CXCR1 genotypes potentially related to interleukin-8 receptor functionality. Immunogenetics. 2007;59(1):53–8.

94. Moretti R, Soglia D, Chessa S, Sartore S, Finocchiaro R, Rasero R, et al. Identification of SNPs associated with somatic cell score in candidate genes in Italian Holstein Friesian bulls. Animals (Basel). 2021;11(2):366.

95. Fontanesi L, Calò DG, Galimberti G, Negrini R, Marino R, Nardone A, et al. A candidate gene association study for nine economically important traits in Italian Holstein cattle. Anim Genet. 2014;45(4):576–80.

96. Beecher C, Daly M, Childs S, Berry DP, Magee DA, McCarthy TV, et al. Polymorphisms in bovine immune genes and their associations with somatic cell count and milk production in dairy cattle. BMC Genet. 2010;11:99.

97. Chen R, Yang Z, Ji D, Mao Y, Chen Y, Zhang Y, et al. SNPs of CXCR1 gene and its associations with somatic cell score in Chinese Holstein cattle. Anim Biotechnol. 2011;22(3):133–42.

98. Leyva-Baca I, Schenkel F, Martin J, Karrow NA. Polymorphisms in the 5' upstream region of the CXCR1 chemokine receptor gene, and their association with somatic cell score in Holstein cattle in Canada. J Dairy Sci. 2008;91(1):407–17.

99. Siebert L, Headrick S, Lewis M, Gillespie B, Young C, Wojakiewicz L, et al. Genetic variation in CXCR1 haplotypes linked to severity of Streptococcus uberis infection in an experimental challenge model. Vet Immunol Immunopathol. 2017;190:45–52.

100. Mao YJ, Zhu XR, Li R, Chen D, Xin SY, Zhu YH, et al. Methylation analysis of CXCR1 in mammary gland tissue of cows with mastitis induced by Staphylococcus aureus. Genet Mol Res. 2015;14(4):12606–15.

101. Alhussien M, Manjari P, Mohammed S, Sheikh AA, Reddi S, Dixit S, et al. Incidence of mastitis and activity of milk neutrophils in Tharparkar cows reared under semi-arid conditions. Trop Anim Health Prod. 2016;48(6):1291–5.

102. Alhussien MN, Dang AK. Pathogen-dependent modulation of milk neutrophils competence, plasma inflammatory cytokines and milk quality during intramammary infection of Sahiwal (Bos indicus) cows. Microb Pathog. 2018;121:131–8.

103. Youngerman SM, Saxton AM, Oliver SP, Pighetti GM. Association of CXCR2 polymorphisms with subclinical and clinical mastitis in dairy cattle. J Dairy Sci. 2004;87(8):2442–8.

104. Rambeaud M, Clift R, Pighetti GM. Association of a bovine CXCR2 gene polymorphism with neutrophil survival and killing ability. Vet Immunol Immunopathol. 2006;111(3-4):23–8.

105. El Nahas SM, El Kasas AH, Abou Mossallem AA, Abdelhamid MI, Warda M. A study on IL8RB gene polymorphism as a potential immuno-compromised adherent in exaggeration of parenteral and mammo-crine oxidative stress during mastitis in buffalo. J Adv Res. 2017;8(6):617–25.

106. Naderi S, Bohlouli M, Yin T, König S. Genomic breeding values, SNP effects and gene identification for disease traits in cow training sets. Anim Genet. 2018;49(3):178–92.

107. Wu X, Lund MS, Sahana G, Guldbrandtsen B, Sun D, Zhang Q, et al. Association analysis for udder health based on SNP-panel and sequence data in Danish Holsteins. Genet Sel Evol. 2015;47:50.

108. Abdel-Shafy H, Bortfeldt RH, Reissmann M, Brockmann GA. Validating genome-wide associated signals for clinical mastitis in German Holstein cattle. Anim Genet. 2018;49(1):82–5.

109. Brodowska P, Zwierzchowski L, Marczak S, Jarmuż W, Bagnicka E. Associations between bovine β-defensin 4 genotypes and production traits of Polish Holstein-Friesian dairy cattle. Animals (Basel). 2019;9(10):723.

110. Khan MZ, Wang D, Liu L, Usman T, Wen H, Zhang R, et al. Significant genetic effects of Significant genetic effects of JAK2 and DGAT1 mutations on milk fat content and mastitis resistance in Holsteins. J Dairy Res. 2019;86(4):388–93.

111. Bouwman AC, Bovenhuis H, Visker MH, van Arendonk JA. Genome-wide association of milk fatty acids in Dutch dairy cattle. BMC Genet. 2011;12:43.

112. Cai Z, Dusza M, Guldbrandtsen B, Lund MS, Sahana G. Distinguishing pleiotropy from linked QTL between milk production traits and mastitis resistance in Nordic Holstein cattle. Genet Sel Evol. 2020;52(1):19.

113. Li L, Tang W, Zhao M, Gong B, Cao M, Li J. Study on the regulation mechanism of lipopolysaccharide on oxidative stress and lipid metabolism of bovine mammary epithelial cells. Physiol Res. 2021;70(5):777–85.

114. Li M, Gao Q, Wang M, Liang Y, Sun Y, Chen Z, et al. Polymorphisms in fatty acid desaturase 2 gene are associated with milk production traits in Chinese Holstein cows. Animals (Basel). 2020;10(4):671.

115. Wang X, Maltecca C, Tal-Stein R, Lipkin E, Khatib H. Association of bovine fibroblast growth factor 2 (FGF2) gene with milk fat and productive life: an example of the ability of the candidate pathway strategy to identify quantitative trait genes. J Dairy Sci. 2008;91(6):2475–80.

116. Jardim JG, Guldbrandtsen B, Lund MS, Sahana G. Association analysis for udder index and milking speed with imputed whole-genome sequence variants in Nordic Holstein cattle. J Dairy Sci. 2018;101(3):2199–212.

117. Sahana G, Guldbrandtsen B, Thomsen B, Holm LE, Panitz F, Brøndum RF, et al. Genome-wide association study using high-density single nucleotide polymorphism arrays and whole-genome sequences for clinical mastitis traits in dairy cattle. J Dairy Sci. 2014;97(11):7258–75.

118. Suravajhala P, Benso A. Prioritizing single-nucleotide polymorphisms and variants associated with clinical mastitis. Adv Appl Bioinform Chem. 2017;10:57–64.

119. Freebern E, Santos DJA, Fang L, Jiang J, Parker Gaddis KL, Liu GE, et al. GWAS and fine-mapping of livability and six disease traits in Holstein cattle. BMC Genom. 2020;21(1):41.

120. Li L, Huang J, Zhang X, Ju Z, Qi C, Zhang Y, et al. One SNP in the 3'-UTR of HMGB1 gene affects the binding of target bta-miR-223 and is involved in mastitis in dairy cattle. Immunogenetics. 2012;64(11):817–24.

121. Cheng WJ, Li QL, Wang CF, Wang HM, Li JB, Sun YM, et al. Genetic polymorphism of HSP70-1 gene and its correlation with resistance to mastitis in Chinese Holstein. Yi Chuan. 2009;31(2):169–74.

122. Huang P, Lu C, Li J, Xu J, Liu Z, Wang Q, et al. Mutations in HSP70-2 gene change the susceptibility to clinical mastitis in Chinese Holstein. Gene. 2015;559(1):62–72.

123. Jiang L, Sørensen P, Thomsen B, Edwards SM, Skarman A, Røntved CM, et al. Gene prioritization for livestock diseases by data integration. Physiol Genom. 2012;44(5):305–17.

124. Verschoor CP, Pant SD, Schenkel FS, Sharma BS, Karrow NA. SNPs in the bovine IL-10 receptor are associated with somatic cell score in Canadian dairy bulls. Mamm Genome. 2009;20(7):447–54.

125. Littlejohn MD, Turner SA, Walker CG, Berry SD, Tiplady K, Sherlock RG, et al. Identification of an immune modulation locus utilising a bovine mammary gland infection challenge model. J Dairy Res. 2018;85(2):185–92.

126. Usman T, Wang Y, Liu C, He Y, Wang X, Dong Y, et al. Novel SNPs in. J Anim Sci Biotechnol. 2017;8:5.

127. Usman T, Yu Y, Liu C, Wang X, Zhang Q, Wang Y. Genetic effects of single nucleotide polymorphisms in JAK2 and STAT5A genes on susceptibility of Chinese Holsteins to mastitis. Mol Biol Rep. 2014;41(12):8293–301.

128. Usman T, Wang Y, Liu C, Wang X, Zhang Y, Yu Y. Association study of single nucleotide polymorphisms in JAK2 and STAT5B genes and their differential mRNA expression with mastitis susceptibility in Chinese Holstein cattle. Anim Genet. 2015;46(4):371–80.

129. Marete A, Sahana G, Fritz S, Lefebvre R, Barbat A, Lund MS, et al. Genome-wide association study for milking speed in French Holstein cows. J Dairy Sci. 2018;101(7):6205–19.

130. Huang J, Wang H, Wang C, Li J, Li Q, Hou M, et al. Single nucleotide polymorphisms, haplotypes and combined genotypes of lactoferrin gene and their associations with mastitis in Chinese Holstein cattle. Mol Biol Rep. 2010;37(1):477–83.

131. Carvajal AM, Huircan P, Lepori A. Single nucleotide polymorphisms in immunity-related genes and their association with mastitis in Chilean dairy cattle. Genet Mol Res. 2013;12(3):2702–11.

132. Wojdak-Maksymiec K, Szyda J, Strabel T. Parity-dependent association between TNF-α and LTF gene polymorphisms and clinical mastitis in dairy cattle. BMC Vet Res. 2013;9:114.

133. Chopra A, Gupta ID, Verma A, Chakravarty AK, Vohra V. Lactoferrin gene promoter variants and their association with clinical and subclinical mastitis in indigenous and crossbred cattle. Pol J Vet Sci. 2015;18(3):465–71.

134. Dinesh K, Verma A, Das Gupta I, Thakur YP, Verma N, Arya A. Identification of polymorphism in exons 7 and 12 of lactoferrin gene and its association with incidence of clinical mastitis in Murrah buffalo. Trop Anim Health Prod. 2015;47(4):643–7.

135. Soyeurt H, Bastin C, Colinet FG, Arnould VM, Berry DP, Wall E, et al. Mid-infrared prediction of lactoferrin content in bovine milk: potential indicator of mastitis. Animal. 2012;6(11):1830–8.

136. Cheng Z, Buggiotti L, Salavati M, Marchitelli C, Palma-Vera S, Wylie A, et al. Global transcriptomic profiles of circulating leucocytes in early lactation cows with clinical or subclinical mastitis. Mol Biol Rep. 2021;48(5):4611–23.

137. Huang JM, Wang ZY, Ju ZH, Wang CF, Li QL, Sun T, et al. Two splice variants of the bovine lactoferrin gene identified in Staphylococcus aureus isolated from mastitis in dairy cattle. Genet Mol Res. 2011;10(4):3199–203.

138. Genini S, Badaoui B, Sclep G, Bishop SC, Waddington D, Pinard van der Laan MH, et al. Strengthening insights into host responses to mastitis infection in ruminants by combining heterogeneous microarray data sources. BMC Genom. 2011;12(1):225.

139. Brand B, Hartmann A, Repsilber D, Griesbeck-Zilch B, Wellnitz O, Kühn C, et al. Comparative expression profiling of E. coli and S. aureus inoculated primary mammary gland cells sampled from cows with different genetic predispositions for somatic cell score. Genet Sel Evol. 2011;43:24.

140. Gilbert FB, Cunha P, Jensen K, Glass EJ, Foucras G, Robert-Granié C, et al. Differential response of bovine mammary epithelial cells to Staphylococcus aureus or Escherichia coli agonists of the innate immune system. Vet Res. 2013;44(1):40.

141. Tanamati F, Stafuzza NB, Gimenez DFJ, Stella AAS, Santos DJA, Ferro MIT, et al. Differential expression of immune response genes associated with subclinical mastitis in dairy buffaloes. Animal. 2019;13(8):1651–7.

142. Yang G, Yue Y, Li D, Duan C, Qiu X, Zou Y, et al. Antibacterial and immunomodulatory effects of Pheromonicin-NM on Escherichia coli-challenged bovine mammary epithelial cells. Int Immunopharmacol. 2020;84:106569.

143. Wu J, Li L, Sun Y, Huang S, Tang J, Yu P, et al. Altered molecular expression of the TLR4/NF-κB signaling pathway in mammary tissue of Chinese Holstein cattle with mastitis. PloS one. 2015;10(2):e0118458.

144. Bhattarai D, Chen X, Ur Rehman Z, Hao X, Ullah F, Dad R, et al. Association of MAP4K4 gene single nucleotide polymorphism with mastitis and milk traits in Chinese Holstein cattle. J Dairy Res. 2017;84(1):76–9.

145. Liu J, Ju Z, Li Q, Huang J, Li R, Li J, et al. Mannose-binding lectin 1 haplotypes influence serum MBL-A concentration, complement activity, and milk production traits in Chinese Holstein cattle. Immunogenetics. 2011;63(11):727–42.

146. Wang C, Liu M, Li Q, Ju Z, Huang J, Li J, et al. Three novel single-nucleotide polymorphisms of MBL1 gene in Chinese native cattle and their associations with milk performance traits. Vet Immunol Immunopathol. 2011;139(2-4):229–36.

147. Yuan Z, Li J, Gao X, Xu S. SNPs identification and its correlation analysis with milk somatic cell score in bovine MBL1 gene. Mol Biol Rep. 2013;40(1):7–12.

148. Fraser RS, Lumsden JS, Lillie BN. Identification of polymorphisms in the bovine collagenous lectins and their association with infectious diseases in cattle. Immunogenetics. 2018;70(8):533–46.

149. Kamaldeep K, Magotra A, Pander BL, Dalal DS, Malik BS, Garg AR, et al. Evaluation of candidate genotype of immune gene MBL1 associated with udder health and performance traits in dairy cattle and buffalo of India. Trop Anim Health Prod. 2021;53(4):429.

150. Wang X, Ju Z, Huang J, Hou M, Zhou L, Qi C, et al. The relationship between the variants of the bovine MBL2 gene and milk production traits, mastitis, serum MBL-C levels and complement activity. Vet Immunol Immunopathol. 2012;148(3-4):311–9.

151. Lei QR, Yang X, Miao CM, Wang JC, Yang Y. Relationship between granulomatous lobular mastitis and methylene tetrahydrofolate reductase gene polymorphism. World J Clin Cases. 2020;8(18):4017–21.

152. Chen N, Wang F, Yu N, Gao Y, Huang J, Dang R, et al. Polymorphisms in MX2 Gene Are Related with SCS in Chinese Dairy Cows. Anim Biotechnol. 2018;29(2):81–9.

153. Zhang Z, Wang X, Li R, Ju Z, Qi C, Zhang Y, et al. Genetic mutations potentially cause two novel NCF1 splice variants up-regulated in the mammary gland, blood and neutrophil of cows infected by Escherichia coli. Microbiol Res. 2015;174:24–32.

154. Ju Z, Wang C, Wang X, Yang C, Sun Y, Jiang Q, et al. Role of an SNP in alternative splicing of bovine NCF4 and mastitis susceptibility. PLoS One. 2015;10(11):e0143705.

155. Ju Z, Wang C, Wang X, Yang C, Zhang Y, Sun Y, et al. The effect of the SNP g.18475 A>G in the 3′UTR of NCF4 on mastitis susceptibility in dairy cattle. Cell Stress and Chaperones. 2018;23(3):385–91.

156. Abdel-Shafy H, Bortfeldt RH, Reissmann M, Brockmann GA. Short communication: validation of somatic cell score-associated loci identified in a genome-wide association study in German Holstein cattle. J Dairy Sci. 2014;97(4):2481–6.

157. Wang HL, Li ZX, Wang LJ, He H, Yang J, Chen L, et al. Polymorphism in PGLYRP-1 gene by PCR-RFLP and its association with somatic cell score in Chinese Holstein. Res Vet Sci. 2013;95(2):508–14.

158. Wang HL, Li ZX, Chen L, Yang J, Wang LJ, He H, et al. Polymorphism in PGLYRP-2 gene by PCR-RFLP and its association with somatic cell score and percentage of fat in Chinese Holstein. Genet Mol Res. 2013;12(4):6743–51.

159. Yang F, Chen F, Li L, Yan L, Badri T, Lv C, et al. Three novel players: PTK2B, SYK, and TNFRSF21 were identified to be involved in the regulation of bovine mastitis susceptibility via GWAS and post-transcriptional analysis. Front Immunol. 2019;10:1579.

160. Martin P, Palhière I, Maroteau C, Clément V, David I, Klopp GT, et al. Genome-wide association mapping for type and mammary health traits in French dairy goats identifies a pleiotropic region on chromosome 19 in the Saanen breed. J Dairy Sci. 2018;101(6):5214–26.

161. Kurz JP, Yang Z, Weiss RB, Wilson DJ, Rood KA, Liu GE, et al. A genome-wide association study for mastitis resistance in phenotypically well-characterized Holstein dairy cattle using a selective genotyping approach. Immunogenetics. 2019;71(1):35–47.

162. Oget C, Allain C, Portes D, Foucras G, Stella A, Astruc JM, et al. A validation study of loci associated with mastitis resistance in two French dairy sheep breeds. Genet Sel Evol. 2019;51(1):5.

163. Rupp R, Senin P, Sarry J, Allain C, Tasca C, Ligat L, et al. A point mutation in suppressor of cytokine signalling 2 (Socs2) increases the susceptibility to inflammation of the mammary gland while associated with higher body weight and size and higher milk production in a sheep model. PLoS genetics. 2015;11(12):e1005629.

164. Bakhtiarizadeh MR, Mirzaei S, Norouzi M, Sheybani N, Vafaei Sadi MS. Identification of gene modules and hub genes involved in mastitis development using a systems biology approach. Front Genet. 2020;11:722.

165. Arun SJ, Thomson PC, Sheehy PA, Khatkar MS, Raadsma HW, Williamson P. Targeted analysis reveals an important role of JAK-STAT-SOCS genes for milk production traits in Australian Dairy cattle. Front Genet. 2015;6:342.

166. Piechotta M, Holzhausen L, Araujo MG, Heppelmann M, Sipka A, Pfarrer C, et al. Antepartal insulin-like growth factor concentrations indicating differences in the metabolic adaptive capacity of dairy cows. J Vet Sci. 2014;15(3):343–52.

167. Riley LG, Gardiner-Garden M, Thomson PC, Wynn PC, Williamson P, Raadsma HW, et al. The influence of extracellular matrix and prolactin on global gene expression profiles of primary bovine mammary epithelial cells in vitro. Anim Genet. 2010;41(1):55–63.

168. Jiang Q, Zhao H, Li R, Zhang Y, Liu Y, Wang J, et al. In silico genome-wide miRNA-QTL-SNPs analyses identify a functional SNP associated with mastitis in Holsteins. BMC Genet. 2019;20(1):46.

169. Alain K, Karrow NA, Thibault C, St-Pierre J, Lessard M, Bissonnette N. Osteopontin: an early innate immune marker of Escherichia coli mastitis harbors genetic polymorphisms with possible links with resistance to mastitis. BMC Genom. 2009;10:444.

170. Dario C, Selvaggi M. Study on the STAT5A/AvaI polymorphism in Jersey cows and association with milk production traits. Mol Biol Rep. 2011;38(8):5387–92.

171. Selvaggi M, Albarella S, Dario C, Peretti V, Ciotola F. Association of STAT5A gene variants with milk production traits in Agerolese cattle. Biochem Genet. 2017;55(2):158–67.

172. Kiyici JM, Akyüz B, Kaliber M, Arslan K, Aksel EG, Cinar MU. Association of GH, STAT5A, MYF5 gene polymorphisms with milk somatic cell count, EC and pH levels of Holstein dairy cattle. Anim Biotechnol. 2022;33(3):401–7.

173. Oikonomou G, Michailidis G, Kougioumtzis A, Avdi M, Banos G. Effect of polymorphisms at the STAT5A and FGF2 gene loci on reproduction, milk yield and lameness of Holstein cows. Res Vet Sci. 2011;91(2):235–9.

174. Raven L-A, Cocks BG, Kemper KE, Chamberlain AJ, Vander Jagt CJ, Goddard ME, et al. Targeted imputation of sequence variants and gene expression profiling identifies twelve candidate genes associated with lactation volume, composition and calving interval in dairy cattle. Mammalian Genome. 2016;27(1):81–97.

175. Cecchinato A, Chessa S, Ribeca C, Cipolat-Gotet C, Bobbo T, Casellas J, et al. Genetic variation and effects of candidate-gene polymorphisms on coagulation properties, curd firmness modeling and acidity in milk from Brown Swiss cows. Animal. 2015;9(7):1104–12.

176. Goldammer T, Zerbe H, Molenaar A, Schuberth HJ, Brunner RM, Kata SR, et al. Mastitis increases mammary mRNA abundance of beta-defensin 5, toll-like-receptor 2 (TLR2), and TLR4 but not TLR9 in cattle. Clin Diagn Lab Immunol. 2004;11(1):174–85.

177. Kannaki TR, Shanmugam M, Verma PC. Toll-like receptors and their role in animal reproduction. Anim Reprod Sci. 2011;125(1–4):1–12.

178. Ruiz-Rodriguez CT, Brandt JR, Oliverio R, Ishida Y, Guedj N, Garrett EF, et al. Polymorphisms of the Toll-like receptor 2 of goats (Capra hircus) may be associated with somatic cell count in milk. Anim Biotechnol. 2017;28(2):112–9.

179. Elmaghraby MM, El-Nahas AF, Fathala MM, Sahwan FM, Tag El-Dien MA. Association of toll-like receptors 2 and 6 polymorphism with clinical mastitis and production traits in Holstein cattle. Iran J Vet Res. 2018;19(3):202–7.

180. Bai J, Lin J, Li W, Liu M. Association of toll-like receptor 2 polymorphisms with somatic cell score in Xinjiang Brown cattle. Anim Sci J. 2012;83(1):23–30.

181. Opsal MA, Lien S, Brenna-Hansen S, Olsen HG, Våge DI. Association analysis of the constructed linkage maps covering TLR2 and TLR4 with clinical mastitis in Norwegian Red cattle. J Anim Breed Genet. 2008;125(2):110–8.

182. An R, Gao M, Meng Y, Tong X, Chen J, Wang J. Infective mastitis due to bovine-associated Streptococcus dysgalactiae contributes to clinical persistent presentation in a murine mastitis model. Vet Med Sci. 2021;7(5):1600–10.

183. Zhang LP, Gan QF, Ma TH, Li HD, Wang XP, Li JY, et al. Toll-like receptor 2 gene polymorphism and its relationship with SCS in dairy cattle. Anim Biotechnol. 2009;20(3):87–95.

184. Farhat K, Sauter KS, Brcic M, Frey J, Ulmer AJ, Jungi TW. The response of HEK293 cells transfected with bovine TLR2 to established pathogen-associated molecular patterns and to bacteria causing mastitis in cattle. Vet Immunol Immunopathol. 2008;125(3-4):326–36.

185. Ma JL, Zhu YH, Zhang L, Zhuge ZY, Liu PQ, Yan XD, et al. Serum concentration and mRNA expression in milk somatic cells of toll-like receptor 2, toll-like receptor 4, and cytokines in dairy cows following intramammary inoculation with Escherichia coli. J Dairy Sci. 2011;94(12):5903–12.

186. Ogorevc J, Simčič M, Zorc M, Škrjanc M, Dovč P. Polymorphism (rs650082970) is associated with somatic cell count in goat milk. PeerJ. 2019;7:e7340.

187. Swain DK, Kushwah MS, Kaur M, Patbandha TK, Mohanty AK, Dang AK. Formation of NET, phagocytic activity, surface architecture, apoptosis and expression of toll like receptors 2 and 4 (TLR2 and TLR4) in neutrophils of mastitic cows. Vet Res Commun. 2014;38(3):209–19.

188. Sharma BS, Leyva I, Schenkel F, Karrow NA. Association of toll-like receptor 4 polymorphisms with somatic cell score and lactation persistency in Holstein bulls. J Dairy Sci. 2006;89(9):3626–35.

189. de Mesquita AQ, E Rezende CS, de Mesquita AJ, Jardim EA, Kipnis AP. Association of TLR4 polymorphisms with subclinical mastitis in Brazilian holsteins. Braz J Microbiol. 2012;43(2):692–7.

190. Panigrahi M, Kumar H, Nayak SS, Rajawat D, Parida S, Bhushan B, et al. Molecular characterization of CRBR2 fragment of TLR4 gene in association with mastitis in Vrindavani cattle. Microb Pathog. 2022;165:105483.

191. Catalani E, Amadori M, Vitali A, Lacetera N. Short communication: Lymphoproliferative response to lipopolysaccharide and incidence of infections in periparturient dairy cows. J Dairy Sci. 2013;96(11):7077–81.

192. Chen H, Liu C, Xiang M, Yu J, Xia Y, Hu X, et al. Contribution of the mutation rs8193069 in TLR4 to mastitis resistance and performance in Holstein cows in southern China. Vet Med Sci. 2022, 8(1):357–66.

193. Wang XP, Luoreng ZM, Gao SX, Guo DS, Li JY, Gao X, et al. Haplotype analysis of TLR4 gene and its effects on milk somatic cell score in Chinese commercial cattle. Mol Biol Rep. 2014;41(4):2345–51.

194. Blum SE, Heller ED, Jacoby S, Krifucks O, Leitner G. Comparison of the immune responses associated with experimental bovine mastitis caused by different strains of Escherichia coli. J Dairy Res. 2017;84(2):190–7.

195. Kandasamy S, Kerr DE. Genomic analysis of between-cow variation in dermal fibroblast response to lipopolysaccharide. J Dairy Sci. 2012;95(7):3852–64.

196. Akhtar M, Guo S, Guo Y, Zahoor A, Shaukat A, Chen Y, et al. Upregulated-gene expression of pro-inflammatory cytokines (TNF-α, IL-1β and IL-6) via TLRs following NF-κB and MAPKs in bovine mastitis. Acta Tropica. 2020;207:105458.

197. Panigrahi M, Sharma A, Bhushan B. Molecular characterization and expression profile of partial TLR4 gene in association to mastitis in crossbred cattle. Anim Biotechnol. 2014;25(3):188–99.

198. Ibeagha-Awemu EM, Lee JW, Ibeagha AE, Bannerman DD, Paape MJ, Zhao X. Bacterial lipopolysaccharide induces increased expression of toll-like receptor (TLR) 4 and downstream TLR signaling molecules in bovine mammary epithelial cells. Vet Res. 2008;39(2):11.

199. Korkmaz FT, Elsasser TH, Kerr DE. Variation in fibroblast expression of toll-like receptor 4 and lipopolysaccharide-induced cytokine production between animals predicts control of bacterial growth but not severity of Escherichia coli mastitis. J Dairy Sci. 2018;101(11):10098–115.

200. Sorg D, Danowski K, Korenkova V, Rusnakova V, Küffner R, Zimmer R, et al. Microfluidic high-throughput RT-qPCR measurements of the immune response of primary bovine mammary epithelial cells cultured from milk to mastitis pathogens. Animal. 2013;7(5):799–805.

201. Xu AJ, Liu XL, Guo JZ, Xia Z. Polymorphism of bovine TNF-a gene and its association with mastitis in Chinese Holstein cows. Yi Chuan. 2010;32(9):929–34.

202. Ranjan S, Bhushan B, Panigrahi M, Kumar A, Deb R, Kumar P, et al. Association and expression analysis of single nucleotide polymorphisms of partial tumor necrosis factor alpha gene with mastitis in crossbred cattle. Anim Biotechnol. 2015;26(2):98–104.

203. Sun Y, Li L, Li C, Wang G, Xing G. Gene microarray integrated with iTRAQ-based proteomics for the discovery of NLRP3 in LPS-induced inflammatory response of bovine mammary epithelial cells. J Dairy Res. 2019;86(4):416–24.

204. Jensen K, Günther J, Talbot R, Petzl W, Zerbe H, Schuberth H-J, et al. Escherichia coli- and Staphylococcus aureus-induced mastitis differentially modulate transcriptional responses in neighbouring uninfected bovine mammary gland quarters. BMC Genom. 2013;14(1):36.

205. Miles AM, Huson HJ. Time- and population-dependent genetic patterns underlie bovine milk somatic cell count. J Dairy Sci. 2020;103(9):8292–304.
